# Comparative clinical effectiveness and safety of tobacco cessation pharmacotherapies and electronic cigarettes: a systematic review and network meta‐analysis of randomized controlled trials

**DOI:** 10.1111/add.15675

**Published:** 2021-10-11

**Authors:** Kyla H. Thomas, Michael N. Dalili, José A. López‐López, Edna Keeney, David M. Phillippo, Marcus R. Munafò, Matt Stevenson, Deborah M. Caldwell, Nicky J. Welton

**Affiliations:** ^1^ Population Health Sciences, Bristol Medical School University of Bristol UK; ^2^ Department of Basic Psychology and Methodology, Faculty of Psychology University of Murcia Murcia Spain; ^3^ Statistical and Health Economic Modelling, Population Health Sciences, Bristol Medical School University of Bristol Bristol UK; ^4^ School of Psychological Science University of Bristol Bristol UK; ^5^ MRC Integrative Epidemiology Unit at the University of Bristol Bristol UK; ^6^ Health Economics and Decision Science, School of Health and Related Research University of Sheffield Sheffield UK

**Keywords:** adverse events, bupropion, effectiveness, electronic cigarettes, network meta‐analysis, nicotine replacement therapy, safety, smoking, varenicline

## Abstract

**Aim:**

To determine how varenicline, bupropion, nicotine replacement therapy (NRT) and electronic cigarettes compare with respect to their clinical effectiveness and safety.

**Method:**

Systematic reviews and Bayesian network meta‐analyses of randomized controlled trials, in any setting, of varenicline, bupropion, NRT and e‐cigarettes (in high, standard and low doses, alone or in combination) in adult smokers and smokeless tobacco users with follow‐up duration of 24 weeks or greater (effectiveness) or any duration (safety). Nine databases were searched until 19 February 2019. Primary outcomes were sustained tobacco abstinence and serious adverse events (SAEs). We estimated odds ratios (ORs) and treatment rankings and conducted meta‐regression to explore covariates.

**Results:**

We identified 363 trials for effectiveness and 355 for safety. Most monotherapies and combination therapies were more effective than placebo at helping participants to achieve sustained abstinence; the most effective of these, estimated with some imprecision, were varenicline standard [OR = 2.83, 95% credible interval (CrI) = 2.34–3.39] and varenicline standard + NRT standard (OR = 5.75, 95% CrI = 2.27–14.88). Estimates were higher in smokers receiving counselling than in those without and in studies with higher baseline nicotine dependence scores than in those with lower scores. Varenicline standard + NRT standard showed a high probability of being ranked best or second‐best. For safety, only bupropion at standard dose increased the odds of experiencing SAEs compared with placebo (OR = 1.27, 95% CrI = 1.04–1.58), and we found no evidence of effect modification.

**Conclusions:**

Most tobacco cessation monotherapies and combination therapies are more effective than placebo at helping participants to achieve sustained abstinence, with varenicline appearing to be most effective based on current evidence. There does not appear to be strong evidence of associations between most tobacco cessation pharmacotherapies and adverse events; however, the data are limited and there is a need for improved reporting of safety data.

## INTRODUCTION

Cigarette smoking is a leading cause of premature mortality and morbidity in the United Kingdom and world‐wide [1, 2], and represents a substantial economic burden. In 2012, the global amount of health‐care expenditure due to smoking‐attributable diseases totalled US$422 billion, while the global total economic cost of smoking (from health expenditures and productivity losses together) totalled US$1436 billion [3]. In the United Kingdom, three pharmacotherapies, varenicline, bupropion and nicotine replacement therapy (NRT), are licensed by the Medicines and Healthcare products Regulatory Agency (MHRA) and recommended by the National Institute for Health and Care Excellence (NICE) for smoking cessation [4]. Although currently marketed electronic cigarettes (e‐cigarettes) are not licensed as tobacco cessation medicines, guidance by NICE and Public Health England advise that they can be considered for smokers who have been unable to quit using other medicines and estimate that their use is 95% safer than smoking conventional cigarettes [4, 5, 6]. However, in the United States, e‐cigarettes are not currently approved by the US Food and Drug Administration (FDA) as a quit smoking aid, and to date no electronic nicotine delivery systems (ENDS) products have been authorized by the FDA [7]. Additionally, ENDS have been banned in more than 30 countries [8].

It is essential that there is a clear understanding of the comparative effectiveness of tobacco cessation pharmacotherapies and e‐cigarettes. However, there is a lack of clinical trials that compare tobacco cessation pharmacotherapies against each other or in combination; most trials estimate the effectiveness of these medicines as monotherapies against placebo. Additionally, given the popularity of e‐cigarette use in the United Kingdom (approximately 3.2 million adult users in 2018) [9], it is important to review their effectiveness compared with licensed tobacco cessation medicines.

Concerns have been raised previously regarding the safety of tobacco cessation medicines, in particular varenicline, bupropion and e‐cigarettes. In July 2009, the FDA placed a Black Box warning around a possible association with serious neuropsychiatric events (i.e. depression, suicidal ideation and behaviour) on varenicline's product labelling [10]. This warning was removed in December 2016 [11], mainly due to the findings of the Evaluating Adverse Events in a Global Smoking Cessation Study (EAGLES) randomized controlled trial (RCT) [12]. However, concerns about the validity of the EAGLES trial have since been raised, as the study was only statistically powered to detect a very large serious adverse effect; therefore, it would not have been able to detect a rare adverse effect such as suicide [13]. Findings from some studies suggested that the use of bupropion for smoking cessation was associated with a greater risk of experiencing seizures [14]. However, the most recent Cochrane Review of antidepressants for smoking cessation [15] found insufficient evidence to conclusively determine whether bupropion was associated with seizures as well as other serious adverse events. Compared to placebo, findings from previous reviews have suggested an increased risk of lower risk cardiovascular disease events associated with the use of NRT [16], an increased risk of nausea, insomnia, abnormal dreams, headache and serious adverse events associated with the use of varenicline [17] and an increased incidence of psychiatric adverse events such as anxiety and insomnia associated with the use of bupropion [15]. Safety concerns concerning e‐cigarettes have been related to the risks of variable manufacturing standards for the devices, risks associated with flavouring components, the possibility of harmful constituents in e‐cigarettes and a lack of evidence regarding the long‐term health impact of e‐cigarettes [18, 19, 20]. The 2019 US outbreak of e‐cigarette or vaping product use‐associated lung injury resulted in approximately 3000 hospitalizations and 68 confirmed deaths. Vitamin E acetate in illicit tetrahydrocannabinol (THC)‐containing products has been strongly implicated in this outbreak [21].

Network meta‐analysis (NMA) is a method that enables comparison of any pair of interventions by pooling direct (head‐to‐head) and indirect evidence from RCTs that form a network of intervention comparisons. NMA delivers the relative effect estimates needed to inform policy and practice even if there is no direct evidence. The most recent review of efficacy was an NMA conducted by the Irish Health Information and Quality Authority (HIQA) [22], which updated data from previous Cochrane Reviews [23] until August 2016. However, since this date a number of new studies have reported, including studies of e‐cigarettes. Reviews of safety have mainly focused upon comparing the safety of tobacco cessation medicines as monotherapies with placebo [24, 25, 26, 27, 28, 29, 30, 31], although comparisons with other active interventions are likely to be of greater clinical relevance to patients, prescribers and regulatory agencies. As more trials report on the use of combinations of tobacco cessation medicines, it is important for reviews to include combined therapies. Additionally, previous safety reviews of RCTs have excluded trials with fewer than 6 months of follow‐up, as they have focused upon including trials based on abstinence outcomes [17, 32]. Therefore, many important adverse events could have been missed.

We aimed to perform comprehensive systematic reviews and NMAs [33] of the effectiveness and safety of varenicline, bupropion, NRT and e‐cigarettes as monotherapies and combination therapies in relation to each other, placebo, waiting‐list, usual care or no drug treatment to enable patients, prescribers and regulators to make informed decisions regarding treatment choices.

## METHODS

The protocol for this study is registered with the Prospective Register of Systematic Reviews (PROSPERO) (CRD42016041302), and has been published [34]. There were some protocol deviations. The inclusion of electronic cigarettes and specification of covariates were decided following the submission of our PROSPERO record and protocol manuscript for peer review. The findings of our analyses of safety data from observational studies with control groups [35] and our cost‐effectiveness analyses [36] are reported elsewhere. We were unable to include and analyse craving and withdrawal data, as these were rarely reported among the included studies and were measured using a variety of measures and scales, so evidence synthesis was impossible. We made a pragmatic decision to only analyse biochemically verified data, as this is considered the recommended standard measure for cessation [37] and is commonly used in reviews. We felt that this decision would retain the most robust data and minimize bias and heterogeneity while keeping the project manageable. Trials that only collected self‐reported data are included in the study characteristics and risk of bias tables in the Supporting information Appendices of our Health Technology Assessment (HTA) report [35]. We had planned to analyse sustained abstinence data from multiple follow‐up times using survival synthesis methods for time to relapse. However, we made a pragmatic decision to analyse as a binary outcome at the 6‐month time‐point.

### Population

We included RCTs in any setting in adult smokers and smokeless tobacco users with a follow‐up duration of 24 weeks or greater (effectiveness) or any duration (safety). We excluded studies in non‐smoking or non‐smokeless tobacco‐using populations and pregnant and breastfeeding women.

Interventions

We included e‐cigarettes and the three UK‐licensed tobacco cessation medicines (varenicline, bupropion and NRT) as monotherapies or in combination. For NRT, combinations of different formulations given concurrently (for example, patch and gum) were also included. We also examined different dosages of treatments (see Table 1). Dosage categories were determined using the British National Formulary and the MHRA public assessment report for the ‘e‐Voke’, the first e‐cigarette to be licensed as a medicine but not currently marketed [38, 39].

**TABLE 1:** Interventions by formulation and dosage.

Treatment (formulation)	Low dose	Standard dose	High dose
Bupropion (oral extended‐release tablets)	< 150 mg bd	150 mg bd	> 150 mg bd
Varenicline (tablets)	< 1 mg bd	1 mg bd	> 1 mg bd
E‐cigarette (electronic inhaler, 5 cartridges/day)	10 mg		15 mg
Nicotine replacement therapy (NRT)			
NRT patch (16 hours)	< 15 mg	15 mg	> 15 mg
NRT patch (24 hours)	< 14 mg	14 mg	> 14 mg
NRT gum (15/day)		2 mg	4 mg
NRT nasal spray (2 sprays/hour, 64/day)		0.5 mg	
NRT mouth spray (4 sprays/hour, 64/day)		1 mg	
NRT lozenge (1 lozenge/1–2 hours, 15/day)	< 2 mg	2 mg	4 mg
NRT sublingual tablet (2 mg/tablet, 40/day)		1/hour	2/hour
NRT inhalator		10 mg (12/day)	15 mg (6/day)

NRT = nicotine replacement therapy; bd = twice daily.

NRT treatments were classified as an NRT combination where two or more NRT products were administered in combination in a single arm and NRT choice, where participants were allowed to select the NRT products they would use. The dosage for NRT combination was indicated based on the highest dose among assigned products, whereas dosage for NRT choice was only identified when a dose was reported for every offered product. Trial arms where patients could receive more than one intervention, but these were not defined (unlike specified combination interventions), were excluded.

Eligible comparators were: other active interventions, placebo (reference comparator for the NMA), no drug treatment, usual care and waiting‐list. ‘Placebo’ includes placebo tablet, NRT or electronic cigarette with non‐nicotine liquid. ‘No drug treatment’ was used when participants were not given any medicine or placebo. ‘Usual care’ was used as defined by the trial authors and did not include any cessation medicines or placebo. Studies of counselling for tobacco cessation were excluded unless one or more trial arms included a tobacco cessation medicine or electronic cigarette.

### Search strategy and data extraction

We searched MEDLINE, Embase, PsycINFO, Web of Science, clinicaltrials.gov and Cochrane Databases (Cochrane Database of Systematic Reviews, Database of Abstracts and Reviews of Effectiveness, Cochrane Central Register of Controlled Trials) and the Health Technology Assessment Database with no language restrictions until 19 February 2019. The search strategy is included in the Supporting information, Appendix, pp. 3–4. We also manually searched reference lists of previous reviews.

At least two reviewers screened abstracts and identified full text reports for inclusion using Covidence (covidence.org). Disagreements were resolved by reaching consensus among reviewers. Data were extracted by one reviewer onto electronic Microsoft Excel worksheets and checked by co‐reviewers. Study authors were contacted in the event of missing data or unclear information. We assessed risk of bias using the Cochrane risk of bias assessment tool for selection bias, performance bias, detection bias, attrition bias, reporting bias and other bias domains [40]. We created an ‘overall risk of bias’ as the highest rating across all domains except the ‘selection bias’ domain, because this was usually rated as ‘unclear risk’ due to few studies that identified study protocols.

### Outcomes

#### Effectiveness

Only biochemically verified events were included. The primary effectiveness outcome was sustained abstinence, defined as avoidance of all tobacco use since the quit day until the time the assessment was made, occasionally allowing for lapses. Secondary effectiveness outcomes included prolonged abstinence (measure of cessation which allows for a grace period following the quit date of up to 2 weeks), any abstinence (abstinence by any definition at 6 months follow‐up) and 7‐day point prevalence abstinence (PPA; measure of cessation based on behaviour at a particular point in time) [41]. With the exception of any abstinence, outcome data from the longest reported time‐point were used.

#### Safety

The primary safety composite outcome was serious adverse events (SAEs), defined as the number of participants experiencing events that resulted in death, were life‐threatening, required hospitalization or resulted in significant disability [42]. Secondary safety composite outcomes included major adverse cardiovascular events (MACEs), including cardiovascular death, non‐fatal myocardial infarction (excluding unstable angina), fatal and non‐fatal stroke [43], and major adverse neuropsychiatric events (MANEs), comprising suicide, attempted suicide, suicidal ideation, depression and seizures [26]. Adverse events were measured as the number of trial participants experiencing an adverse event.

### Data analysis

All outcomes were binary, extracted using the intention‐to‐treat principle where participants missing from analyses were assumed to be using tobacco (effectiveness) or not having experienced an adverse event or SAE (safety). Where there were no events in at least one but not all arms, we added 0.5 events to all cells in the 2 × 2 table for that trial [44].

Random‐effects NMAs were conducted within a Bayesian framework using OpenBUGS (version 3.2.3). Studies comparing pharmacological treatment plus counselling versus counselling alone were included and analysed as pharmacological treatment compared with no drug treatment, under the assumption of no interaction when pharmacological treatment and counselling are used together. Studies comparing pharmacological treatment plus counselling versus usual care were analysed as pharmacological treatment compared with no drug treatment and the impact of the addition of counselling was estimated using meta‐regression. We ran a sensitivity analysis to exclude such studies.

Heterogeneity was assessed by examining the between‐study standard deviation (SD) (τ) and 95% credible intervals (CrIs). We fitted a standard (full interaction) NMA model as well as fixed and random class NMA models for each outcome. Model fit was measured by the posterior mean residual deviance and models compared using the deviance information criterion (DIC). Differences of three or more were considered meaningful. The consistency assumption was assessed by comparing model fit, DIC and variance parameters for a model which relaxes consistency (unrelated mean effects model) with the standard NMA model [45]. We also compared direct and indirect estimates where both were available. Results are presented as posterior median odds ratios (OR) and 95% CrIs. Although we report 95% CrIs we consider ‘statistical significance levels’ to be a continuum [46], so the further the lower credible limit is above 1 the stronger the evidence of effect, and the width of credible intervals indicate levels of precision. We used vague normal priors for all treatment effect parameters and uniform (0.5) priors for all standard deviation parameters. Full details are reported in Thomas *et al*. [35] We also report the probability that each intervention class is ranked best, second best, and so on, across outcomes using rank‐o‐grams [33].

### Meta‐regression

We performed meta‐regression [47] to explore the influence of several pre‐specified covariates: counselling, industry sponsorship, treatment duration, baseline nicotine dependence score, comorbidities, willingness to quit, smokeless tobacco, smoking level and publication year. We performed sensitivity analyses excluding studies at high risk of bias for the primary outcomes (see Supporting information, Appendix, p. 5). As an alternative to grading of recommendations, assessment, development and evaluations (GRADE) [48], a threshold analysis was performed for the primary outcomes to assess the credibility of the results [49] and robustness of treatment rankings to potential biases or uncertainty in the evidence [48, 50]. The method estimates thresholds which indicate how much the evidence could change (for any reason, such as bias or random error) before the treatment rankings or recommendations change. By comparing the thresholds with judgements of the plausible magnitude of potential biases and estimates of uncertainty (confidence intervals) we can identify comparisons where conclusions are robust and comparisons where conclusions are sensitive to plausible biases or uncertainty in the evidence. We used threshold analysis, as it makes explicit the links between the sources of evidence, their quality and the treatment rankings by accounting for the influence of evidence on the rankings, and is therefore more directly applicable to treatment rankings and recommendations, whereas tools such as GRADE only consider the quality of evidence [48, 49].

### Ethical approval

Ethical approval for this evidence synthesis was not required.

## RESULTS

**FIGURE 1:**
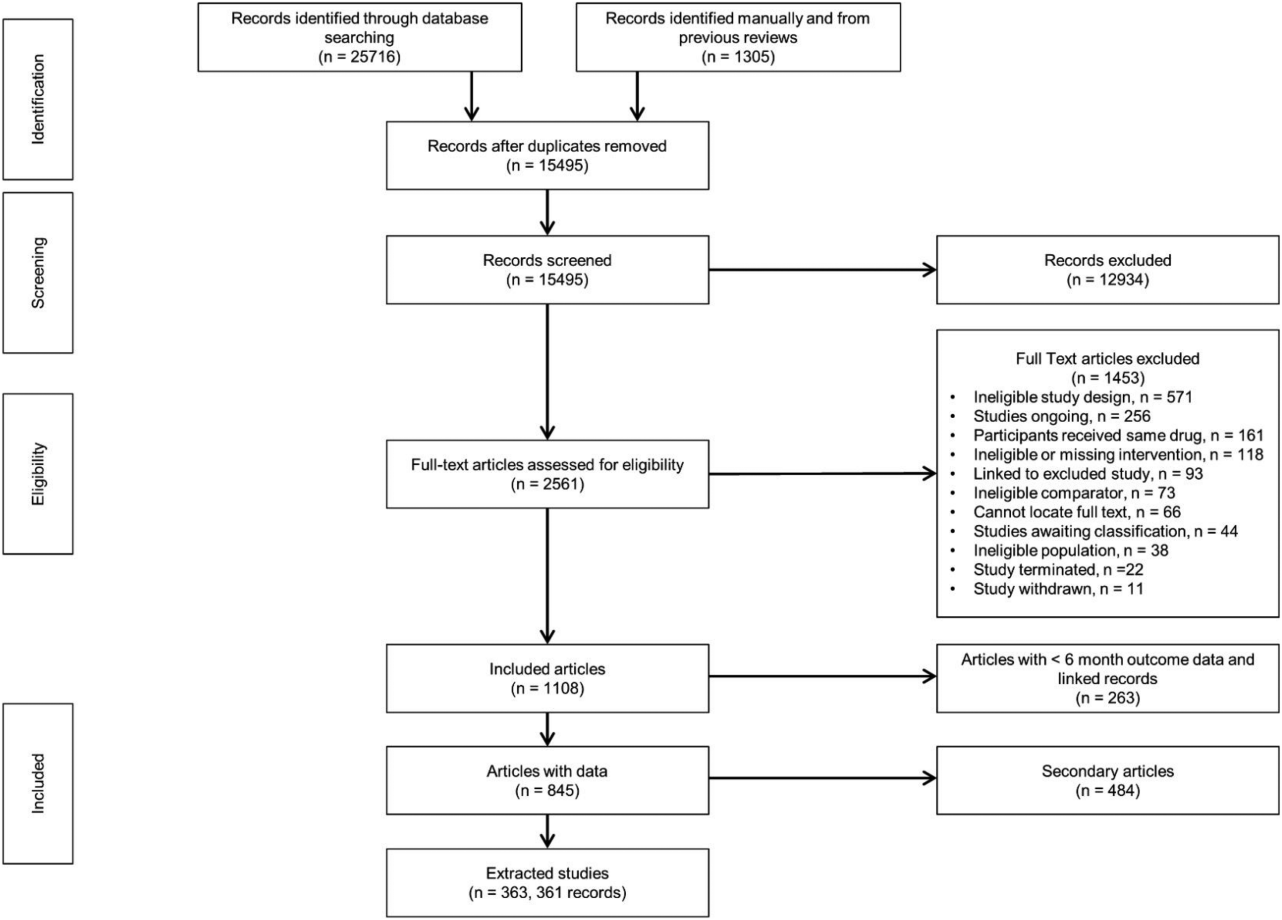
Preferred Reporting Items for Systematic Reviews and Meta‐Analyses (PRISMA) flow diagram for effectiveness study records

Full results are reported in Thomas *et al*. [35]. We screened 15 495 records and reviewed 2561 full text articles (Figure 1). The EAGLES study [12] was treated as two separate studies for our analyses, Anthenelli 2016A from the non‐psychiatric cohort and Anthenelli 2016B from the psychiatric cohort.

### Effectiveness

We included 363 trials from 361 articles with a total of 201 045 participants (Supporting information, Appendix, pp. 6–21).

Trials were conducted across six continents with 208 US trials, 29 UK trials and 27 multi‐centre international trials. The studies ranged in duration from 6 months to 14.5 years, with duration of drug treatment from 2 weeks to 2 years. Trial participants included a mix of ethnicities, with a mean age ranging from 27 to 62 years. The overall risk of bias for included studies was rated as 40% high, 47% unclear and 13% low. A risk of bias assessment summary figure is available in the Supporting information, Figure S1.

For all outcomes, model fit indices favoured fixed class NMA models, and there was no evidence of inconsistency for this model (Supporting information, Table S1). There was moderate heterogeneity for all efficacy outcomes (Supporting information, Tables S2–S9). Of the included trials, 252 trials contributed to at least one NMA for effectiveness outcomes. Fifty‐one trials were not included in analyses because they did not report any biochemically verified outcomes.

#### Sustained abstinence

**FIGURE 2:**
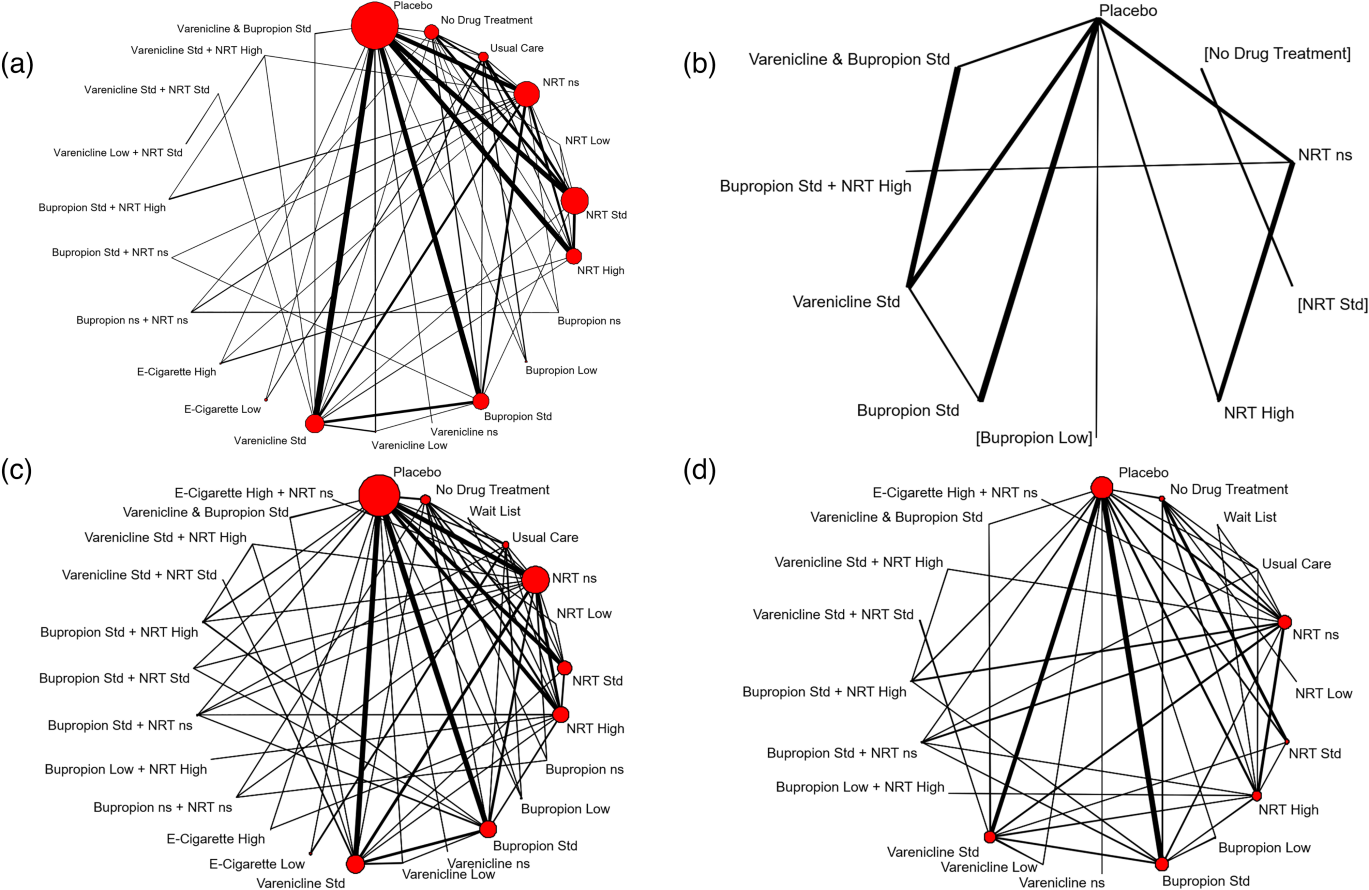
Network meta‐analysis of eligible comparisons for sustained abstinence (a), prolonged abstinence (b), any abstinence (c) and 7‐day point prevalence abstinence (PPA) (d). Thicker edges in network figures represent comparisons with a higher number of randomized patients, while interventions with a larger number of randomized patients have larger circles. Interventions were excluded if they were disconnected from the main network

A total of 171 studies (90 443 participants) reported on sustained abstinence at a follow‐up of at least 24 weeks, of which 161 (86 884 participants) compared two or more of the intervention classes of interest (Figure 2a).

**FIGURE 3:**
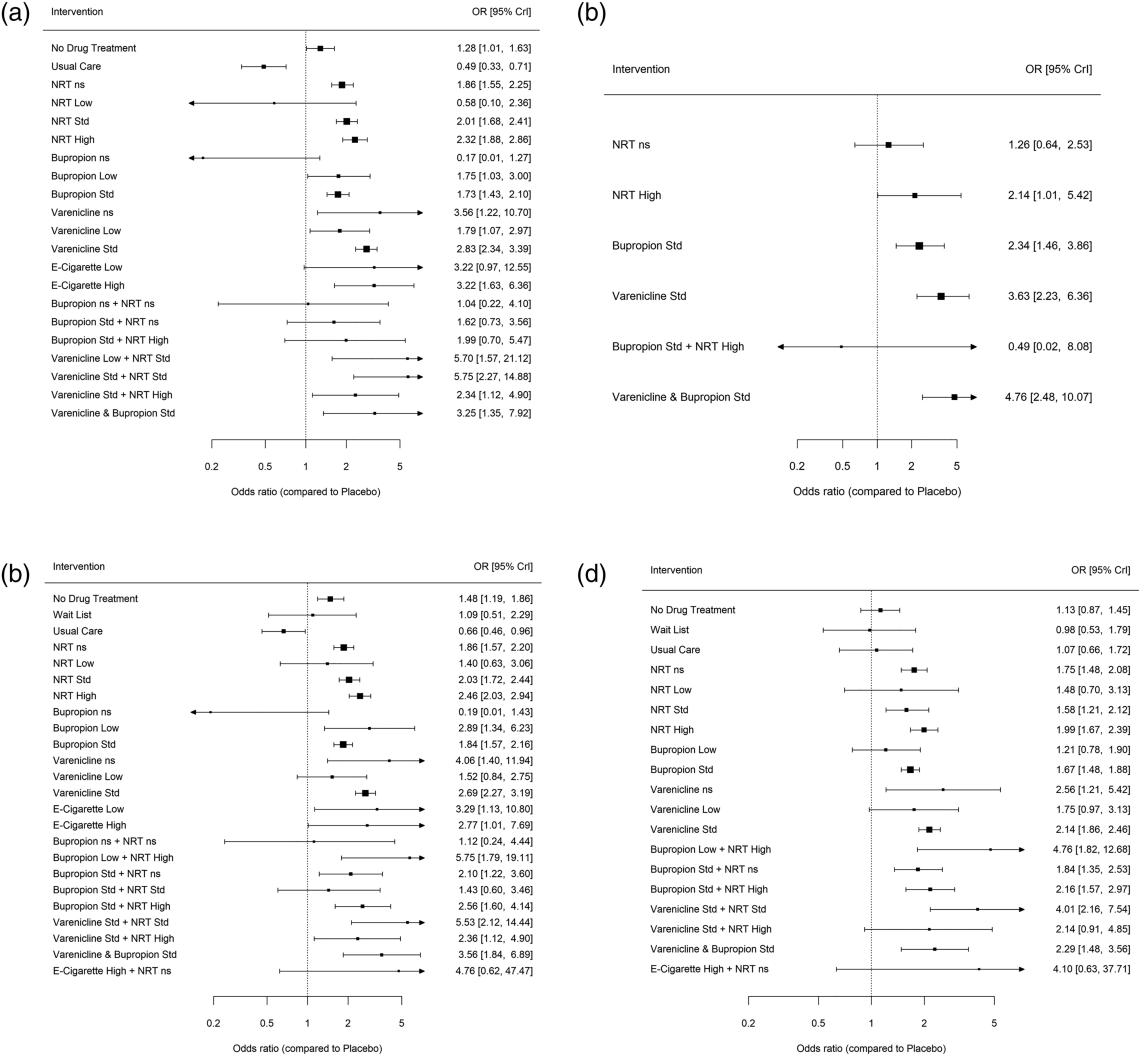
Forest plot with results of the fixed class network meta‐analysis (NMA) model for sustained abstinence (a), prolonged abstinence (b), any abstinence (c) and 7‐day point prevalence abstinence (PPA) (d)

ORs for sustained abstinence are reported in Figure 3a (see also Supporting information, Table S2). Most interventions were more effective than placebo, although some estimates were extremely imprecise. For interventions estimated with some precision there was evidence that smokers receiving varenicline standard (OR = 2.83, 95% CrI = 2.34–3.39), NRT high (OR = 2.32, 95% CrI = 1.88–2.86), NRT standard (OR = 2.01, 95% CrI = 1.68–2.41), varenicline low (OR = 1.79, 95% CrI = 1.07–2.97), bupropion low (OR = 1.75, 95% CrI = 1.03–3.00) and bupropion standard (OR = 1.73, 95% CrI = 1.43–2.10) were more likely to quit relative to placebo. There was evidence that varenicline standard + NRT standard (OR = 5.75, 95% CrI = 2.27–14.88), varenicline low + NRT standard (OR = 5.70, 95% CrI = 1.57–21.12), varenicline standard + bupropion standard (OR = 3.25, 95% CrI = 1.35–7.92), e‐cigarette high (OR = 3.22, 95% CrI = 1.63–6.36) and varenicline standard + NRT high (OR = 2.34, 95% CrI = 1.12–4.90) were effective relative to placebo, but these estimates are extremely imprecise. There was an indication that e‐cigarette low may be more effective than placebo (OR = 3.22, 95% CrI = 0.97–12.55); however, this estimate is extremely imprecise, and the CrIs also incorporate the possibility of no effect.

Most effect estimates from pairwise comparisons between interventions for sustained abstinence were informed by indirect evidence only, and results were consistent when both direct and indirect evidence were available (Supporting information, Table S3). There was evidence that smokers randomized to varenicline standard + NRT standard were more likely to achieve sustained abstinence than those receiving NRT standard (OR = 2.87, 95% CrI = 1.11–7.49) or bupropion standard (OR = 3.34, 95% CrI = 1.28–8.65). Results also suggest higher odds of abstinence for varenicline standard versus NRT standard (OR = 1.40, 95% CrI = 1.10–1.78) and bupropion standard (OR = 1.63, 95% CrI = 1.27–2.07).

##### Meta‐regression results

There was evidence of effect modification as a function of counselling, with interventions that included counselling being associated with higher ORs for achieving sustained abstinence (ratio of ORs = 2.36, 95% CrI = 1.57–3.56). We also found a higher OR of sustained abstinence among participants with higher average baseline nicotine dependence scores (ORs ratio = 1.26, 95% CrI = 1.02–1.54). We found no evidence of effect modification for any other covariates. Excluding studies at high risk of bias yielded similar findings, although with wider intervals for most effect estimates, particularly for e‐cigarettes and treatment combinations. The threshold analysis (Supporting information, Appendix, pp. 27–28) shows that our finding that varenicline standard + NRT standard has the highest estimated odds ratio (OR = 5.75, 95% CrI = 2.27–14.88) is robust, but sensitive to the level of imprecision and potential biases in five studies (three rated high or unclear risk of bias), which could lead to either varenicline + bupropion standard , e‐cigarette low or e‐cigarette high being ranked first for sustained abstinence. However, any possible biases in low/unclear risk of bias‐rated studies would have to alter the OR by at least a factor of 1.6 to change the first‐place ranking.

#### Prolonged, any abstinence and 7‐day PPA

Similar results to those for sustained abstinence were obtained for the other abstinence outcomes (Supporting information, Appendix, pp. 29–35). Relative to placebo, smokers treated with standard doses of varenicline (OR = 3.63, 95% CrI = 2.23–6.36) and bupropion (OR = 2.34, 95% CrI = 1.46–3.86) as monotherapies and in combination (OR = 4.76, 95% CrI = 2.48–10.10) were more likely to achieve prolonged abstinence (Figure 3b). There were no available data for combined varenicline and NRT at standard doses for this outcome.

For the any abstinence outcome (Figure 3c), standard varenicline was more effective than standard doses of NRT (OR = 1.32, 95% CrI = 1.05–1.65) and bupropion (OR = 1.46, 95% CrI = 1.18–1.81). Combined varenicline and NRT at standard doses was also more effective than standard doses of NRT (OR = 2.70, 95% CrI = 1.02 to 7.13) and bupropion (OR = 2.99, 95% CrI = 1.13–7.88). These findings were also observed for the 7‐day point prevalence abstinence outcome (Figure 3d).

**FIGURE 4:**
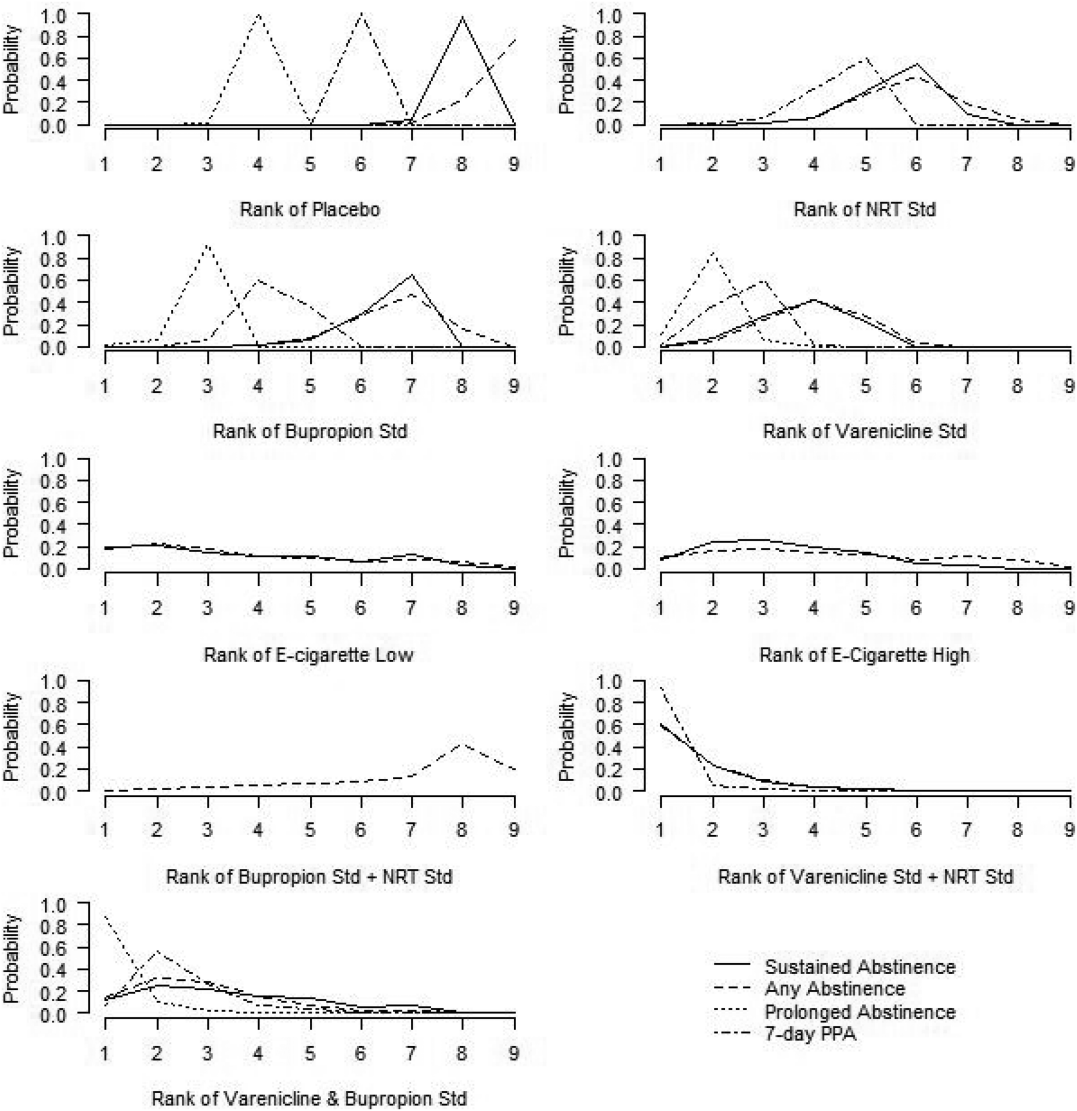
Rank‐o‐gram of intervention classes (at standard doses with the exception of e‐cigarettes) across effectiveness outcomes. All nine intervention classes contributed to the ranking for any abstinence, whereas eight intervention classes were included for sustained abstinence [bupropion standard + nicotine replacement therapy (NRT) had no data], six for 7‐day point prevalence abstinence (PPA) (e‐cigarette low, e‐cigarette high and bupropion standard + NRT standard had no data) and four for prolonged abstinence (no data for NRT standard, e‐cigarette low, e‐cigarette high, bupropion standard + NRT standard, varenicline standard + NRT standard)

Varenicline standard + NRT standard showed a high probability to be ranked best or second‐best intervention for three outcomes (although there was no information available for this drug combination on prolonged abstinence) (Figure 4). Varenicline standard + bupropion standard yielded the highest probability to be ranked as the best intervention for prolonged abstinence, although there was higher uncertainty concerning its ranking for the other outcomes. Varenicline standard showed the highest probabilities to be ranked second‐ to fourth‐best for the different outcomes, whereas e‐cigarettes presented a more uncertain ranking profile. Placebo was ranked as the least effective option for all outcomes. The findings for the standard doses also held in the rank‐o‐grams across all doses (Supporting information, Figure S3).

As an indication of absolute effects, sustained abstinence probabilities are given for a UK population by applying the odds ratios from the NMA to the probability of 1‐year continuous cessation based on NRT standard taken from Taylor *et al*. [51] (Supporting information, Table S10).

### Safety

**FIGURE 5:**
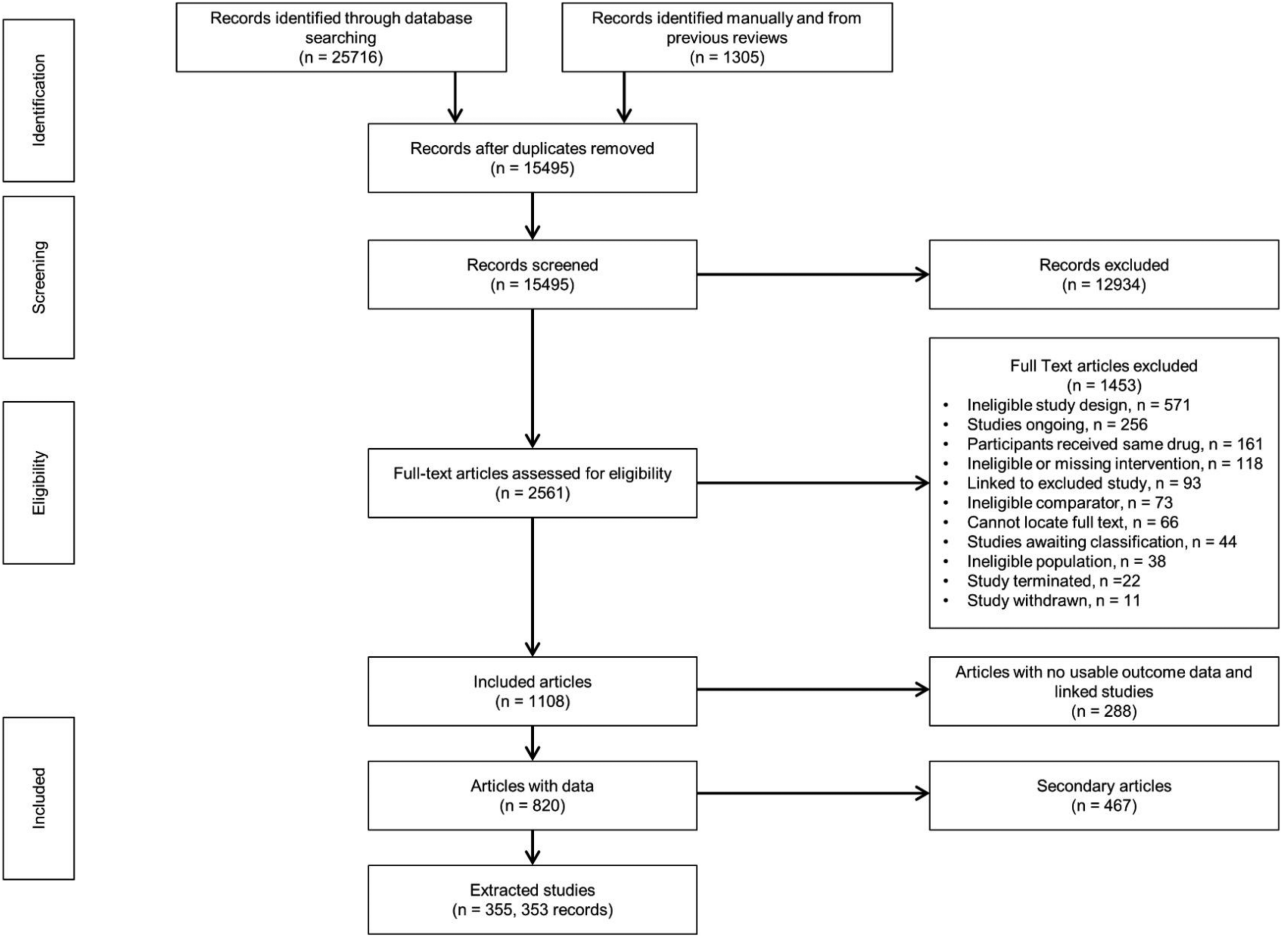
Preferred Reporting Items for Systematic Reviews and Meta‐Analyses (PRISMA) flow diagram for safety study records

We included 355 trials from 353 articles with a total of 159 101 participants (Supporting information, Appendix, pp. 39–51) (Figure 5).

Trials were conducted across six continents, with 211 US trials, 34 UK trials and 31 multi‐centre international trials. Trial duration ranged from 1 day/single session to 14.5 years, and duration of drug treatment ranged from half a day to 2 years.

Trial participants included a mix of ethnicities, with a mean age ranging from 28.4 to 62.8 years. The overall risk of bias for included trials was rated as 33% high, 51% unclear and 16% low. A risk of bias summary figure is available in the Supporting information, Figure S4.

For all outcomes, model fit indices favoured fixed‐class NMA models, and there was no evidence of inconsistency for this model (Supporting information, Table S11). There was very little heterogeneity for SAE, but moderate heterogeneity for other safety outcomes (Supporting information, Tables S12–S17). Of the included trials, 149 trials contributed to at least one NMA for safety outcomes.

#### SAEs

**FIGURE 6:**
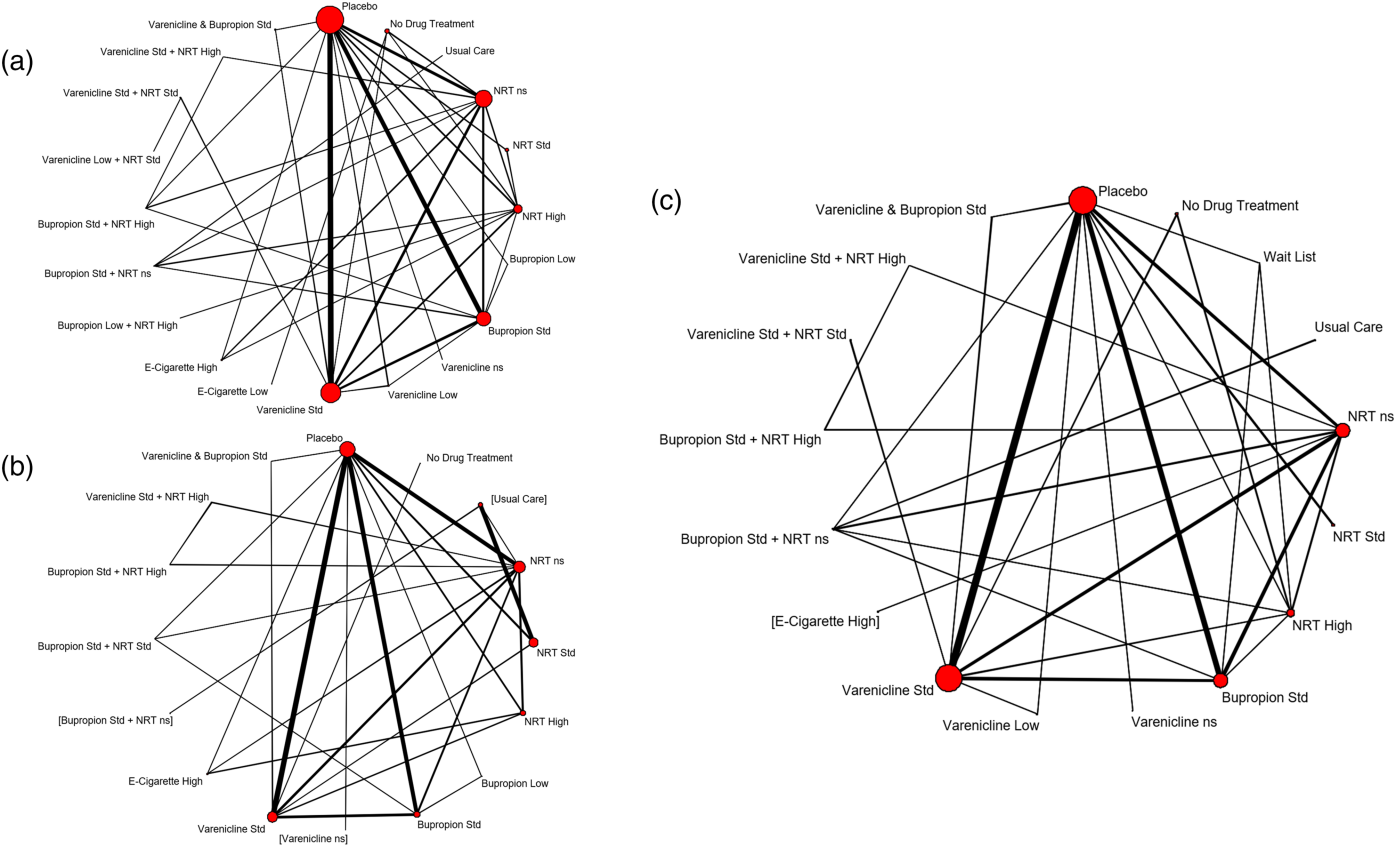
Network meta‐analysis of eligible comparisons for serious adverse events (a), major adverse cardiovascular events (b) and major adverse neuropsychiatric events (c). Thicker edges in network figures represent comparisons with a higher number of randomized patients, while interventions with a larger number of randomized patients have larger circles

A total of 111 trials reported on SAEs with a total of 63 927 patients, of which 101 (58 318 patients) compared two or more of the treatment classes of interest (Figure 6a). We excluded one study [52] from all analyses due to small event numbers causing computational problems.

**FIGURE 7:**
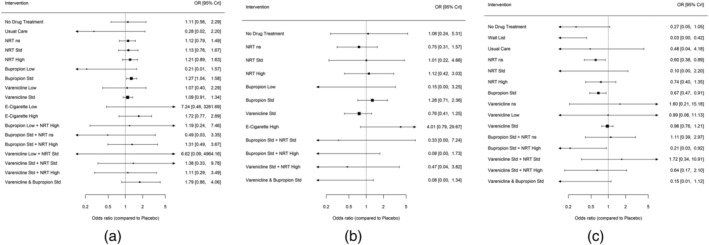
Forest plot with results of the fixed‐class network meta‐analysis (NMA) model for serious adverse events (a), major adverse cardiovascular events (b) and major adverse neuropsychiatric events (c)

Figure 7a (and Supporting information, Table S12) displays the class‐level NMA results for each intervention relative to placebo. There was evidence that bupropion standard (OR = 1.27, 95% CrI = 1.04–1.58) increased the odds of SAEs compared to placebo. The 95% CrIs for all other treatments compared with placebo crossed 1 (no effect).

Most effect estimates for comparisons between active interventions were informed by indirect evidence only (Supporting information, Table S13). As a consequence of this, and also due to the small event rates reported, effects were imprecisely estimated and all 95% CrIs contained 1 (no effect).

##### Meta‐regression results

There was no evidence of effect modification for any factors explored. Excluding trials at high risk of bias yielded similar results, although with wider intervals for most effect estimates [35]. The threshold analyses show that the best‐ and worst‐ranked treatments are sensitive to uncertainty and potential biases in the data (Supporting information, Appendix, pp. 56–57), indicating that we cannot draw robust conclusions from these data.

#### MACEs

A total of 49 trials (38 329 patients) reported MACEs, with 44 trials (36 231 patients) including at least one relevant comparison (Figure 6b). We discarded three trials [52, 53, 54] from all analyses due to small numbers causing computational problems.

Due to the small numbers of events reported across trials, all effect estimates show very wide intervals and hence it was not possible to reliably estimate differences in comparison to placebo (Figure 7b, Supporting information, Table S14) or between pairs of interventions (Supporting information, Table S15).

#### MANEs

MANEs were reported in 75 trials (42 088 patients), with 73 trials (41 483 patients) including at least one relevant comparison. Placebo, NRT not specified, bupropion standard and varenicline standard were the best‐represented interventions (Figure 6c). We excluded two trials [55, 56] from all analyses due to small numbers causing computational problems.

The class‐level NMA results for MANEs for each intervention class relative to placebo are presented in Figure 7c (and Supporting information, Table S16) and show wide intervals around the effect estimates due to small numbers. There was evidence that smokers randomized to waiting‐list (OR = 0.03, 95% CrI = 0.00–0.44), bupropion standard + NRT high (OR = 0.21, 95% CrI = 0.03–0.92), NRT not specified (OR = 0.60, 95% CrI = 0.36–0.89) and bupropion standard (OR = 0.67, 95% CrI = 0.47–0.91) were less likely to report MANEs compared to placebo.

Although most effect estimates were imprecisely estimated due to small numbers, there was evidence of an increased odds of MANEs for smokers randomized to varenicline standard compared to those allocated to bupropion standard (OR = 1.43, 95% CrI = 1.02–2.09) (Supporting information, Table S17).

**FIGURE 8:**
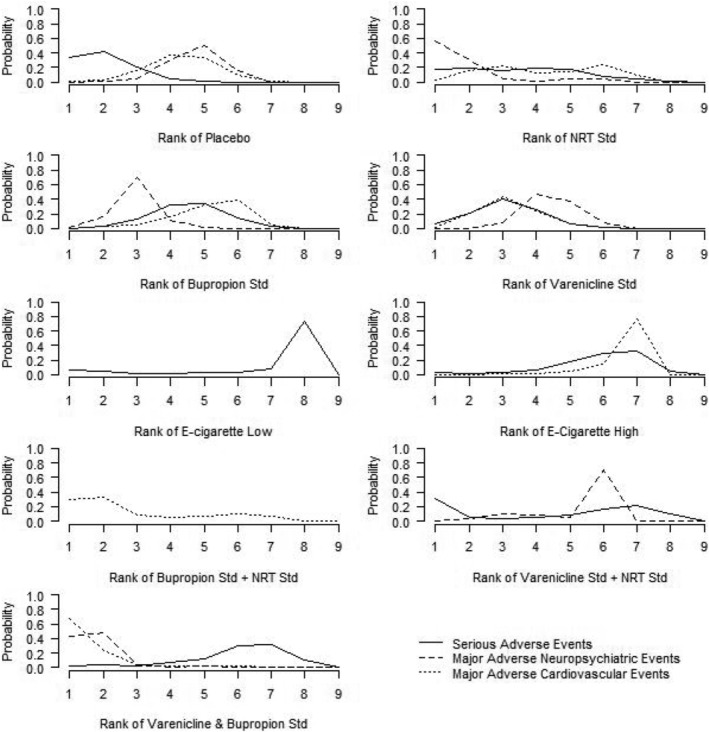
Rank‐o‐gram of interventions across safety outcomes. Eight intervention classes contributed to the ranking for serious adverse events [bupropion standard + nicotine standard replacement therapy (NRT) had no data], whereas six intervention classes were included for major adverse neuropsychiatric events (e‐cigarette low, e‐cigarette high and bupropion standard + NRT standard had no data) and seven for major adverse cardiovascular events (e‐cigarette low and varenicline standard + NRT standard had no data)

Placebo was most likely to be ranked best or second‐best out of nine interventions for SAEs, but ranked in the middle for MACEs and MANEs (Figure 8). NRT standard was also most likely to be ranked among the best two interventions to reduce the odds of SAEs, with uncertain rankings for the other adverse outcomes. Note, however, that all these rankings are based on imprecise effect estimates and may not be robust.

As an indication of absolute effects, the average proportion of patients with an event in the placebo arm across trials for safety outcomes are given in the Supporting information, Table S18.

## DISCUSSION

To our knowledge, this is the largest NMA to examine the effectiveness and safety of tobacco cessation pharmacotherapies and e‐cigarettes, and the first NMA with respect to SAE and MANEs.

### Principal findings

#### Effectiveness

Most monotherapies and combination therapies were more effective than placebo at helping participants to achieve sustained abstinence. Compared to placebo, the most effective therapy that was estimated with some precision was varenicline standard. Varenicline standard + NRT standard, varenicline low + NRT standard, e‐cigarette high and e‐cigarette low show potential to be effective; however, the estimates are extremely imprecise. Smokers randomized to a combination of varenicline and NRT at standard doses were also more likely to achieve sustained abstinence than participants receiving standard NRT or bupropion as monotherapies. Standard doses of varenicline were more effective than standard doses of NRT or bupropion monotherapies. There was evidence that interventions delivered with counselling were more effective than the same interventions delivered without counselling, and effects were greater in studies on participants with higher baseline nicotine dependence scores.

Similar results to those for sustained abstinence were obtained for the other abstinence outcomes. Among almost all outcomes, combined varenicline and NRT at standard doses had the highest probability of being ranked as the best or second‐best, e‐cigarette rankings were uncertain and placebo consistently ranked last.

#### Safety

While the use of bupropion standard may increase the odds of SAEs compared to placebo, we did not find strong evidence of any other negative associations between tobacco cessation medicines and SAEs, MACEs or MANEs relative to placebo. In pairwise comparisons between interventions there was evidence of an increased odds of MANEs for smokers randomized to varenicline standard compared to those using bupropion standard. When ranking the interventions among primary and secondary safety outcomes, placebo and NRT standard were most likely to be ranked among the best interventions for reducing the odds of experiencing SAEs, but were ranked lower for MACEs and MANEs. The safety profile of e‐cigarettes was uncertain.

### Strengths and weaknesses

One of the most significant strengths of this study is the inclusion of combinations of tobacco cessation pharmacotherapies, whereas previous analyses examined only monotherapies or combination NRT. This is also the first NMA to compare medicines stratified by dosage, which allowed more specific identification of the impact of dose across different outcomes and avoided heterogeneity. We were also able to include recent large trials, such as the e‐cigarette trial by Hajek *et al*. [56]. A further strength is the methodology employed, conducting NMAs for multiple cessation outcomes in addition to using the most rigorous definition of abstinence (biochemically verified sustained abstinence). The size of the study also allowed investigation of several important covariates as potential effect modifiers. For safety, our decision to include RCTs of any duration ensured that we maximized the use of available data.

There are several important limitations of this study. Our searches used to retrieve publications are more than 2 years old, so our study may not include more recent findings, especially with respect to e‐cigarettes, where several trials were ongoing and some have since been published. However, this study remains the largest network meta‐analysis of tobacco cessation medicines to date. Despite the large number of studies included data limitations remained, such that comparisons between active interventions were almost exclusively informed by indirect evidence, resulting in imprecisely estimated effects and wide confidence intervals which included the null value. While stratifying by dose was a strength of our study, this has contributed to some imprecision in the results. Additionally, in some instances extreme results were obtained based on the findings of a single or very few trials, which may be particularly problematic when attempting to draw conclusions regarding the safety of e‐cigarettes. We used the longest follow‐up time reported, which varied between studies and could have introduced heterogeneity. A small number of studies were cluster‐randomized; however, intracluster correlations were not available and we were unable to adjust for clustering, which would give slightly less precise estimates. A large proportion of studies were rated as being at high or uncertain risk of bias, as many studies were at risk of selective reporting or did not adequately report random sequence generation and allocation concealment. Although we endeavoured to obtain unpublished data and contacted study authors for additional material, we are aware that data may still be missing from our analyses. Despite extensive efforts we were unable to obtain safety data for industry‐funded trials from pharmaceutical companies, and our findings are limited to those events reported in published articles. Safety outcomes included rare events, which limited the ability of analyses to draw firm conclusions. Additionally, we excluded pregnant or breastfeeding women from this study, as not all the included interventions are licensed for use in this population. However, we acknowledge that this is an important and understudied population with a critical need of support to stop using tobacco [57]. We made an assumption that the effect of counselling is additive when given together with a pharmacotherapy, which is a potential limitation of our findings. It may be that there is a synergistic (or even antagonistic) effect of counselling when used together with pharmacotherapies. We explored this in a sensitivity analysis and found that there was some evidence to support a synergistic effect [35]. Future research to explore this potential synergistic effect of smoking cessation medicines being used together with counselling would be of value. Finally, we acknowledge the decision to only analyse biochemically verified cessation data as a study limitation, as this ultimately decreased the number of studies and the amount of data included in our analyses, and we recognize that a lack of biochemical verification should not be used as an indicator of study quality. The use of biochemical verification is impractical for several study designs, has drawbacks and self‐reported cessation is often considered adequate in the absence of special circumstances [37].

### Comparison to other studies

Our findings are largely comparable to those of previous NMAs [22, 23]. We found evidence that nearly all identified doses of tobacco cessation medicines increased the probability of sustained abstinence compared to placebo. For combined bupropion + NRT, although the HIQA report [22] found evidence that this treatment improved the likelihood of cessation (from the quit date or PPA) compared to placebo (control), this was only observed in our analyses for ‘any abstinence’ and 7‐day PPA outcomes, and not for sustained or prolonged outcomes. Similar to our findings, previous NMAs also found evidence that monotherapy with varenicline increased the chance of cessation compared to bupropion and to NRT, while finding inconclusive evidence of a difference in likelihood of quitting between bupropion and NRT [22, 23]. Findings were also consistent for combined varenicline + NRT, which showed improved probability of quitting compared to bupropion and NRT monotherapies. Although the HIQA report showed that combined varenicline and bupropion was more effective than bupropion or NRT delivered as monotherapies, we did not [22]. However, we stratified our analyses by dose, while theirs did not. The results of ranking of tobacco cessation treatments were similar throughout NMAs [22, 23]. Our results were also comparable to those of the latest relevant Cochrane Systematic Reviews: we similarly found very imprecise evidence that e‐cigarettes led to higher quit rates than placebo and NRT [58], that varenicline was more effective for achieving sustained abstinence than placebo (at low and standard doses), bupropion and NRT [17], that bupropion standard increased sustained abstinence compared to placebo [15] and that various forms of NRT were more effective than placebo at standard and high doses [32].

Two previous NMAs [22, 23] only narratively summarized safety data from previous systematic reviews without further analysis. Unlike some previous reviews [22, 58] we included all SAEs, regardless of whether or not study authors attributed them to intervention use, resulting in the inclusion of an additional study of electronic cigarettes [59] for our SAE outcome analyses. The findings of our NMA of MACEs mirrors that of Mills and colleagues [16], who also found no evidence of any tobacco cessation treatment increasing the likelihood of experiencing a MACE compared to placebo or each other. As pairwise comparisons between active interventions were almost entirely based on indirect evidence only and because MACEs were uncommon, it was extremely difficult to effectively compare treatments to each other. Compared to a recent Cochrane Review [17], we did not find strong evidence that varenicline increased the chance of experiencing SAEs relative to placebo, but we found evidence of an increased odds of MANEs for smokers randomized to varenicline standard compared to bupropion standard. In contrast to a recent review [15], we found evidence that bupropion standard increased the odds of serious adverse events compared to placebo. However, we stratified analyses by dose while the review did not, and it included no pharmacotherapy controls in addition to placebo as comparators.

### CONCLUSIONS AND FUTURE RESEARCH

Regardless of the aforementioned limitations, this study strengthens the evidence base for the use of varenicline and NRT monotherapies as first‐line choices for tobacco cessation, in line with current NICE recommendations [4], and should provide some reassurance to patients, clinicians and policymakers regarding the safety of most of these treatments. While bupropion was effective, it was associated with increased odds of experiencing a SAE. Although e‐cigarettes showed promise as cessation tools, more research is needed on their long‐term effectiveness and safety, preferably in studies with active interventions as comparators. Our findings also suggest an important role for the use of combination tobacco cessation therapies (for example, varenicline and NRT), which may offer smokers a better chance of successfully quitting tobacco over short and long time‐periods. Although combination NRT is commonly prescribed, the other drug combinations are sometimes not licensed. While not the focus of this paper, we found that combining counselling and pharmacological treatments increased cessation rates compared to pharmacological treatment alone. Further research to explore the effectiveness of combination pharmacological treatment and counselling or psychological interventions is likely to be of value. Researchers should ensure comprehensive reporting of safety data in trials to ensure completeness of reporting within studies as well as improve the consistency of reporting across studies.

## PROSPERO REGISTRATION

PROSPERO number CRD42016041302.

## DECLARATION OF INTERESTS

K.T. reports grants and other financial support from the National Institute for Health Research (NIHR) for this study. N.W. and M.S. report grants from the National Institute for Health Research during the conduct of the study and Honoraria from the Association of British Pharmaceutical Industry outside the submitted work. D.P. reports grants from the Medical Research Council during the conduct of the study, personal fees from the Association of the British Pharmaceutical Industry and personal fees from UCB outside the submitted work. E.K. reports personal fees from Novartis Pharma and Pfizer outside the submitted work. M.M. reports grants from Pfizer outside the submitted work. J.L.L., E.K. and M.D. were funded by the National Institute for Health Research for the project. The funder of the study had no role in the study design, data collection, data analysis, data interpretation or writing of the report. The corresponding author had full access to all the data in the study and had final responsibility for the decision to submit for publication.

## AUTHOR CONTRIBUTIONS

**Kyla Thomas:** Conceptualization; data curation; formal analysis; funding acquisition; investigation; methodology; project administration; resources; software; supervision; validation; visualization. **Michael Dalili:** Data curation; formal analysis; investigation; methodology; project administration; validation; visualization. **José López‐López:** Data curation; formal analysis; investigation; methodology; software; validation; visualization. **Edna Keeney:** Data curation; formal analysis; investigation; methodology; software; validation; visualization. **David Phillippo:** Formal analysis; investigation; methodology; validation; visualization. **Marcus Munafo:** Conceptualization; funding acquisition; methodology. **Matt Stevenson:** Formal analysis; funding acquisition; investigation; methodology; software; validation. **DM Caldwell:** Conceptualization; data curation; formal analysis; funding acquisition; investigation; methodology. **Nicky Welton:** Conceptualization; data curation; formal analysis; funding acquisition; investigation; methodology; project administration; software; supervision; validation; visualization.

## Supporting information


**Table S1** Comparison of different NMA models for effectiveness outcomes
**Table S2** Results for sustained abstinence: comparisons with placebo
**Table S3** Results for sustained abstinence: pair‐wise comparisons of interventions
**Figure S1** Risk of bias summary
**Figure S2** Threshold analysis results for sustained abstinence, sorted by size of threshold (smallest to largest).
**Table S4** Results for prolonged abstinence: comparisons with placebo
**Table S5** Results for prolonged abstinence: pair‐wise comparisons of interventions
**Table S6** Results for any abstinence: comparisons with placebo
**Table S7** Results for any abstinence: pair‐wise comparisons of interventions
**Table S8** Results for PPA: comparisons with placebo
**Table S9** Results for PPA: pair‐wise comparisons of interventions
**Figure S3** Rank‐o‐gram of intervention classes at all doses across effectiveness outcomes.
**Table S10** Absolute probability of 1‐year continuous cessation based on NRT Std taken from Taylor *et al* (2017), and odds ratios estimated from the NMA
**Figure S4** Risk of bias summary figure
**Table S11** Comparison of different NMA models for safety outcomes
**Table S12** Results for serious adverse events: comparisons with placebo
**Table S13** Results for serious adverse events: pair‐wise comparisons of interventions
**Figure S5** Threshold analysis results for serious adverse events (first‐ranked treatment), sorted by size of threshold (smallest to largest)
**Figure S6** Threshold analysis results for serious adverse events (last‐ranked treatment), sorted by size of threshold (smallest to largest)
**Table S14** Results for major adverse cardiovascular events: comparisons with placebo
**Table S15** Results for major adverse cardiovascular events: pair‐wise comparisons of interventions
**Table S16** Results for major adverse neuropsychiatric events: comparisons with placebo
**Table S17** Results for major adverse neuropsychiatric events: pair‐wise comparisons of interventions
**Table S18** Average proportion of patients with an event in the placebo arm across trials for each safety outcome.
