# Supplementary material for: Comparative clinical effectiveness and safety of tobacco cessation pharmacotherapies and electronic cigarettes: a systematic review and network meta‐analysis of randomized controlled trials
Source: Addiction. 2021 Oct 11;117(4):861–76. doi: 10.1111/add.15675 (PMC9293179; doi:10.1111/add.15675)
Supplement: Supplementary file 1 — Table S1 Comparison of different NMA models for effectiveness outcomes Table S2 Results for sustained abstinence: comparisons with placebo Table S3 Results for sustained abstinence: pair‐wise comparisons of interventions Figure S1 Risk of bias summary Figure S2 Threshold analysis results for sustained abstinence, sorted by size of threshold (smallest to largest). Table S4 Results for prolonged abstinence: comparisons with placebo Table S5 Results for prolonged abstinence: pair‐wise comparisons of interventions Table S6 Results for any abstinence: comparisons with placebo Table S7 Results for any abstinence: pair‐wise comparisons of interventions Table S8 Results for PPA: comparisons with placebo Table S9 Results for PPA: pair‐wise comparisons of interventions Figure S3 Rank‐o‐gram of intervention classes at all doses across effectiveness outcomes. Table S10 Absolute probability of 1‐year continuous cessation based on NRT Std taken from Taylor et al (2017), and odds ratios estimated from the NMA Figure S4 Risk of bias summary figure Table S11 Comparison of different NMA models for safety outcomes Table S12 Results for serious adverse events: comparisons with placebo Table S13 Results for serious adverse events: pair‐wise comparisons of interventions Figure S5 Threshold analysis results for serious adverse events (first‐ranked treatment), sorted by size of threshold (smallest to largest) Figure S6 Threshold analysis results for serious adverse events (last‐ranked treatment), sorted by size of threshold (smallest to largest) Table S14 Results for major adverse cardiovascular events: comparisons with placebo Table S15 Results for major adverse cardiovascular events: pair‐wise comparisons of interventions Table S16 Results for major adverse neuropsychiatric events: comparisons with placebo Table S17 Results for major adverse neuropsychiatric events: pair‐wise comparisons of interventions Table S18 Average proportion of patients with an event in the placebo arm across tri [file ADD-117-861-s001.docx]

**Comparative clinical effectiveness and safety of tobacco cessation pharmacotherapies and electronic cigarettes: a systematic review and network meta-analysis of randomised controlled trials**

Kyla H Thomas, Michael N Dalili, José A López-López, Edna Keeney, David M Phillippo, Marcus R Munafò, Matt Stevenson, Deborah M Caldwell, Nicky J Welton

**Supplementary Appendix**

EFFECTIVENESS

# Search strategy

Medline Search

Search Strategy:

--------------------------------------------------------------------------------

1 Smoking/ (134671)

2 Tobacco Smoking/ (397)

3 Tobacco/ (29151)

4 Nicotine/ (24376)

5 Tobacco Products/ (3005)

6 Smoking Cessation/ (26370)

7 "Tobacco Use Cessation"/ (1045)

8 "Tobacco Use Disorder"/ (10555)

9 (smoking or smoker*).ti,ab,kf. (231897)

10 (tobacco* or cigar* or cigarette* or nicotine).ti,ab,kf. (164203)

11 or/1-10 (353307)

12 Bupropion/ (2887)

13 Varenicline/ (1147)

14 Nicotinic Agonists/ (6990)

15 (NRT or nicotine replacement).ti,ab,kf. (3901)

16 bupropion.ti,ab,kf. (4038)

17 (amfebutamone or quomen or wellbutrin or zyban or zyntabac).ti,ab,kf. (201)

18 varenicline.ti,ab,kf. (1578)

19 (champix or tabex or chantix).ti,ab,kf. (125)

20 (nicotine adj2 (patch* or gum* or nasal spray* or lozenge* or tablet* or sublingual* or inhal* or replacement or chewing or polac* or transdermal* or product*)).ti,ab,kf. (5818)

21 (nicorette or niquitin or nicotinell).ti,ab,kf. (110)

22 (nicotinic adj3 agonist*).ti,ab,kf. (2237)

23 (benzazepine* adj2 derivative*).ti,ab,kf. (84)

24 nicotinic receptor partial agonist*.ti,ab,kf. (58)

25 or/12-24 (17839)

26 Electronic Nicotine Delivery Systems/ (2174)

27 Vaping/ (237)

28 (electr* adj2 (cig* or nicotine or device*)).ti,ab,kf. (17861)

29 (ecig* or e-cig*).ti,ab,kf. (2982)

30 (nicotine adj4 (electr* or ENDS or aerosol*)).ti,ab,kf. (1023)

31 (vape or vaper or vapers or vaping or vapor or vapour).ti,ab,kf. (42208)

32 or/26-31 (60589)

33 ((smoking or tobacco) adj5 (cessation or ceas* or quit* or stop* or giv* or prevent* or abstain* or abstin* or control*)).ti,kf. (18876)

34 randomized controlled trial.pt. (475058)

35 controlled clinical trial.pt. (92883)

36 pragmatic clinical trial.pt. (951)

37 clinical trial.pt. (514264)

38 clinical trial/ or clinical trial, phase i/ or clinical trial, phase ii/ or clinical trial, phase iii/ or clinical trial, phase iv/ or controlled clinical trial/ (575942)

39 Random Allocation/ (97344)

40 randomized controlled trial/ (475058)

41 pragmatic clinical trial/ (951)

42 Double-Blind Method/ (149187)

43 Single-Blind Method/ (26163)

44 Placebos/ (34201)

45 ((clin* or randomi?ed) adj5 trial*).ti,ab,kf. (586919)

46 ((singl* or doubl* or trebl* or tripl*) adj5 (blind* or mask*)).ti,ab,kf. (163772)

47 placebo*.ti,ab,kf. (202178)

48 control groups/ (1604)

49 randomi?ation.ti,ab,kf. (32653)

50 randomly.ab. (304261)

51 (random* adj3 (administ* or allocat* or assign* or class* or control* or determine* or divide* or distribut* or expose* or fashion or number* or place* or recruit* or subsitut* or treat*)).ab. (429094)

52 drug therapy.fs. (2077773)

53 trial.ti,ab,kf. (534702)

54 groups.ab. (1874309)

55 (control* adj3 (trial* or study or studies)).ab,ti. (470250)

56 ((singl* or doubl* or tripl* or trebl*) adj3 (blind* or mask* or dummy*)).mp. (222854)

57 (quasi adj (experimental or random$)).ti,ab. (15129)

58 ((waitlist* or wait* list* or treatment as usual or TAU) adj3 (control or group)).ab. (5264)

59 or/34-58 (4771652)

60 11 and (25 or 32) and 59 (6074)

61 (animals not (humans and animals)).sh. (4506319)

62 60 not 61 (5269)

63 (2017* or 2018*).yr,dp,dt,ep,ez. (2712640)

64 62 and 63 (740)

65 (2018* or 2019*).yr,dp,dt,ep,ez. (1556937)

66 62 and 65 (438)

# Definition of covariates

We performed meta-regression ^1^ to explore the influence of several covariates as potential effect modifiers for primary effectiveness and safety outcomes, namely:

- Dependence: we combined average scores several scales at the arm level with a hierarchy of preferred measurements. Specifically, we used scores using the Fagerström Test for Nicotine Dependence (FTND) ^2^ in preference to the Fagerström Tolerance Questionnaire (FTQ), ^3^ with Heaviness of Smoking Index (HSI) ^4^ as a third alternative. Average scores from each arm were standardised to make numbers comparable across different scales.
- Funding source: industry vs. non-industry sponsorship.
- Counselling: interventions that included counselling vs. those not including it.
- Type of placebo: for placebo arms, we examined the influence of including a drug placebo (alone or combined with NRT placebo) in comparison to NRT only placebo arms.
- Duration of treatment in each arm (in weeks).
- Studies including samples with all (or most) participants having one or more current psychiatric condition (for example depression, schizophrenia, bipolar disorder, substance misuse) vs. other studies.
- Studies including samples in which all (or most) participants had one or more of the 17 comorbidities specified by the Charlson Comorbidity Index ^5^ vs. other studies.
- Studies in which patients were not required to make a quit attempt vs. other studies.
- Studies focused on smokeless tobacco consumers vs. other studies.
- Studies where patients were heavy smokers vs. other studies. We defined samples of heavy smokers as those in which the average of smoked cigarettes was above 20 per day.
- Publication year

Furthermore, we performed sensitivity analyses excluding different subsets of studies assessed at high risk of bias on any domain.

1. Berkey CS, Hoaglin DC, Mosteller F, Colditz GA. A random-effects regression model for meta-analysis. Stat Med. 1995;14(4):395-411.

2. Heatherton TF, Kozlowski LT, Frecker RC, Fagerstrom KO. The Fagerstrom Test for Nicotine Dependence: a revision of the Fagerstrom Tolerance Questionnaire. British journal of addiction. 1991;86(9):1119-27.

3. Fagerstrom KO. Measuring degree of physical dependence to tobacco smoking with reference to individualization of treatment. Addictive behaviors. 1978;3(3-4):235-41.

4. Heatherton TF, Kozlowski LT, Frecker RC, Rickert W, Robinson J. Measuring the heaviness of smoking: using self-reported time to the first cigarette of the day and number of cigarettes smoked per day. British journal of addiction. 1989;84(7):791-9.

5. Deyo RA, Cherkin DC, Ciol MA. Adapting a clinical comorbidity index for use with ICD-9-CM administrative databases. Journal of clinical epidemiology. 1992;45(6):613-9.

# Reference list of included studies

1. Abdullah AS, Hedley AJ, Chan SS, Lam TH. A randomized controlled trial of two different lengths of nicotine replacement therapy for smoking cessation. Biomed Res Int. 2013;2013(961751):961751.

2. Abelin T, Buehler A, Muller P, Vesanen K, Imhof PR. Controlled trial of transdermal nicotine patch in tobacco withdrawal. Lancet. 1989;1(8628):7-10.

3. Ahluwalia JS, McNagny SE, Clark WS. Smoking cessation among inner-city African Americans using the nicotine transdermal patch. J Gen Intern Med. 1998;13(1):1-8.

4. Ahluwalia JS, Harris KJ, Catley D, Okuyemi KS, Mayo MS. Sustained-release bupropion for smoking cessation in African Americans: a randomized controlled trial. JAMA. 2002;288(4):468-74.

5. Ahluwalia JS, Okuyemi K, Nollen N, Choi WS, Kaur H, Pulvers K, et al. The effects of nicotine gum and counseling among African American light smokers: a 2 x 2 factorial design. Addiction. 2006;101(6):883-91.

6. Anderson CM, Cummins SE, Kohatsu ND, Gamst AC, Zhu SH. Incentives and Patches for Medicaid Smokers: An RCT. Am J Prev Med. 2018;55(6 Suppl 2):S138-S47.

7. Andrews JO, Mueller M, Dooley M, Newman SD, Magwood GS, Tingen MS. Effect of a smoking cessation intervention for women in subsidized neighborhoods: A randomized controlled trial. Prev Med. 2016;90:170-6.

8. Anthenelli RM, Morris C, Ramey TS, Dubrava SJ, Tsilkos K, Russ C, et al. Effects of varenicline on smoking cessation in adults with stably treated current or past major depression: a randomized trial. Ann Intern Med. 2013;159(6):390-400.

9. Anthenelli RM, Benowitz NL, West R, St Aubin L, McRae T, Lawrence D, et al. Neuropsychiatric safety and efficacy of varenicline, bupropion, and nicotine patch in smokers with and without psychiatric disorders (EAGLES): a double-blind, randomised, placebo-controlled clinical trial. Lancet. 2016;387(10037):2507-20.

10. Anthonisen NR, Connett JE, Kiley JP, Altose MD, Bailey WC, Buist AS, et al. Effects of smoking intervention and the use of an inhaled anticholinergic bronchodilator on the rate of decline of FEV1. The Lung Health Study. Jama. 1994;272(19):1497-505.

11. Areechon W, Punnotok J. Smoking Cessation through the Use of Nicotine Chewing Gum - a Double-Blind Trial in Thailand. Clinical Therapeutics. 1988;10(2):183-6.

12. Aryanpur M, Hosseini M, Masjedi MR, Mortaz E, Tabarsi P, Soori H, et al. A randomized controlled trial of smoking cessation methods in patients newly-diagnosed with pulmonary tuberculosis. BMC Infect Dis. 2016;16(369):369.

13. Aubin HJ, Lebargy F, Berlin I, Bidaut-Mazel C, Chemali-Hudry J, Lagrue G. Efficacy of bupropion and predictors of successful outcome in a sample of French smokers: a randomized placebo-controlled trial. Addiction. 2004;99(9):1206-18.

14. Aubin HJ, Bobak A, Britton JR, Oncken C, Billing CB, Jr., Gong J, et al. Varenicline versus transdermal nicotine patch for smoking cessation: results from a randomised open-label trial. Thorax. 2008;63(8):717-24.

15. Baker A, Richmond R, Haile M, Lewin TJ, Carr VJ, Taylor RL, et al. A randomized controlled trial of a smoking cessation intervention among people with a psychotic disorder. American Journal of Psychiatry. 2006;163(11):1934-42.

16. Baker TB, Piper ME, Stein JH, Smith SS, Bolt DM, Fraser DL, et al. Effects of Nicotine Patch vs Varenicline vs Combination Nicotine Replacement Therapy on Smoking Cessation at 26 Weeks: A Randomized Clinical Trial. JAMA. 2016;315(4):371-9.

17. Baldassarri SR, Bernstein SL, Chupp GL, Slade MD, Fucito LM, Toll BA. Electronic cigarettes for adults with tobacco dependence enrolled in a tobacco treatment program: A pilot study. Addict Behav. 2018;80:1-5.

18. Batra A, Klingler K, Landfeldt B, Friederich HM, Westin A, Danielsson T. Smoking reduction treatment with 4-mg nicotine gum: a double-blind, randomized, placebo-controlled study. Clin Pharmacol Ther. 2005;78(6):689-96.

19. Bernstein SL, D'Onofrio G, Rosner J, O'Malley S, Makuch R, Busch S, et al. Successful Tobacco Dependence Treatment in Low-Income Emergency Department Patients: A Randomized Trial. Ann Emerg Med. 2015;66(2):140-7.

20. Binnie VI, McHugh S, Jenkins W, Borland W, Macpherson LM. A randomised controlled trial of a smoking cessation intervention delivered by dental hygienists: a feasibility study. BMC Oral Health. 2007;7:5.

21. Bjurlin MA, Cohn MR, Kim DY, Freeman VL, Lombardo L, Hurley SD, et al. Brief smoking cessation intervention: a prospective trial in the urology setting. J Urol. 2013;189(5):1843-9.

22. Blondal T. Controlled trial of nicotine polacrilex gum with supportive measures. Arch Intern Med. 1989;149(8):1818-21.

23. Blondal T, Franzon M, Westin A. A double-blind randomized trial of nicotine nasal spray as an aid in smoking cessation. Eur Respir J. 1997;10(7):1585-90.

24. Blondal T, Gudmundsson LJ, Olafsdottir I, Gustavsson G, Westin A. Nicotine nasal spray with nicotine patch for smoking cessation: randomised trial with six year follow up. BMJ. 1999;318(7179):285-8.

25. Bohadana A, Nilsson F, Rasmussen T, Martinet Y. Nicotine inhaler and nicotine patch as a combination therapy for smoking cessation: a randomized, double-blind, placebo-controlled trial. Arch Intern Med. 2000;160(20):3128-34.

26. Bolliger CT, Zellweger JP, Danielsson T, van Biljon X, Robidou A, Westin A, et al. Smoking reduction with oral nicotine inhalers: double blind, randomised clinical trial of efficacy and safety. BMJ. 2000;321(7257):329-33.

27. Bolliger CT, van Biljon X, Axelsson A. A nicotine mouth spray for smoking cessation: a pilot study of preference, safety and efficacy. Respiration. 2007;74(2):196-201.

28. Bolliger CT, Issa JS, Posadas-Valay R, Safwat T, Abreu P, Correia EA, et al. Effects of varenicline in adult smokers: a multinational, 24-week, randomized, double-blind, placebo-controlled study. Clin Ther. 2011;33(4):465-77.

29. Bonevski B, Twyman L, Paul C, D'Este C, West R, Siahpush M, et al. Smoking cessation intervention delivered by social service organisations for a diverse population of Australian disadvantaged smokers: A pragmatic randomised controlled trial. Prev Med. 2018;112:38-44.

30. Boyle R, Severson H, Lichtenstein E, et al. Smokeless tobacco cessation with nicotine reduction: A placebo controlled trial [abstract]. Paper presented at: 121st Annual Meeting Location: San Francisco, CA. 1992.

31. Brown RA, Niaura R, Lloyd-Richardson EE, Strong DR, Kahler CW, Abrantes AM, et al. Bupropion and cognitive-behavioral treatment for depression in smoking cessation. Nicotine Tob Res. 2007;9(7):721-30.

32. Buchkremer G, Bents H, Horstmann M, Opitz K, Tölle R. Combination of behavioral smoking cessation with transdermal nicotine substitution. Addictive Behaviors. 1989;14(2):229-38.

33. Buchkremer G, Minneker E. Efficiency of multimodal smoking cessation therapy combining transdermal nicotine substitution with behavioral therapy. Methods and findings in experimental and clinical pharmacology. 1989;11(3):215-8.

34. Bullen C, Howe C, Lin RB, Grigg M, Laugesen M, McRobbie H, et al. Pre-cessation nicotine replacement therapy: pragmatic randomized trial. Addiction. 2010;105(8):1474-83.

35. Bullen C, Howe C, Laugesen M, McRobbie H, Parag V, Williman J, et al. Electronic cigarettes for smoking cessation: A randomised controlled trial. The Lancet. 2013;382(9905):1629-37.

36. Burns EK, Hood NE, Goforth E, Levinson AH. Randomised trial of two nicotine patch protocols distributed through a state quitline. Tob Control. 2016;25(2):218-23.

37. Caldwell BO, Adamson SJ, Crane J. Combination rapid-acting nicotine mouth spray and nicotine patch therapy in smoking cessation. Nicotine Tob Res. 2014;16(10):1356-64.

38. Caldwell BO, Crane J. Combination Nicotine Metered Dose Inhaler and Nicotine Patch for Smoking Cessation: A Randomized Controlled Trial. Nicotine Tob Res. 2016;18(10):1944-51.

39. Caldwell AL, Tingen MS, Nguyen JT, Andrews JO, Heath J, Waller JL, et al. Parental Smoking Cessation: Impacting Children's Tobacco Smoke Exposure in the Home. Pediatrics. 2018;141(Suppl 1):S96-S106.

40. Campbell IA. Comparison of 4 Methods of Smoking Withdrawal in Patients with Smoking Related Diseases. British Medical Journal. 1983;286(6365):595-7.

41. Campbell IA, Lyons E, Prescott RJ. Stopping Smoking - Do Nicotine Chewing-Gum and Postal Encouragement Add to Doctors Advice. Practitioner. 1987;231(1423):114-7.

42. Campbell IA, Prescott RJ, Tjeder-Burton SM. Smoking cessation in hospital patients given repeated advice plus nicotine or placebo chewing gum. Respir Med. 1991;85(2):155-7.

43. Campbell IA, Prescott RJ, Tjeder-Burton SM. Transdermal nicotine plus support in patients attending hospital with smoking-related diseases: a placebo-controlled study. Respir Med. 1996;90(1):47-51.

44. Caponnetto P, Campagna D, Cibella F, Morjaria JB, Caruso M, Russo C, et al. EffiCiency and Safety of an eLectronic cigAreTte (ECLAT) as tobacco cigarettes substitute: a prospective 12-month randomized control design study. PLoS One. 2013;8(6):e66317.

45. Carpenter MJ, Hughes JR, Gray KM, Wahlquist AE, Saladin ME, Alberg AJ. Nicotine therapy sampling to induce quit attempts among smokers unmotivated to quit: a randomized clinical trial. Arch Intern Med. 2011;171(21):1901-7.

46. Carson KV, Smith BJ, Brinn MP, Peters MJ, Fitridge R, Koblar SA, et al. Safety of varenicline tartrate and counseling versus counseling alone for smoking cessation: a randomized controlled trial for inpatients (STOP study). Nicotine Tob Res. 2014;16(11):1495-502.

47. Chan SS, Leung DY, Abdullah AS, Wong VT, Hedley AJ, Lam TH. A randomized controlled trial of a smoking reduction plus nicotine replacement therapy intervention for smokers not willing to quit smoking. Addiction. 2011;106(6):1155-63.

48. Chengappa KN, Perkins KA, Brar JS, Schlicht PJ, Turkin SR, Hetrick ML, et al. Varenicline for smoking cessation in bipolar disorder: a randomized, double-blind, placebo-controlled study. J Clin Psychiatry. 2014;75(7):765-72.

49. Cinciripini PM, Cinciripini LG, Wallfisch A, Haque W, Van Vunakis H. Behavior therapy and the transdermal nicotine patch: effects on cessation outcome, affect, and coping. J Consult Clin Psychol. 1996;64(2):314-23.

50. Cinciripini PM, Robinson JD, Karam-Hage M, Minnix JA, Lam C, Versace F, et al. Effects of varenicline and bupropion sustained-release use plus intensive smoking cessation counseling on prolonged abstinence from smoking and on depression, negative affect, and other symptoms of nicotine withdrawal. JAMA Psychiatry. 2013;70(5):522-33.

51. Cinciripini PM, Minnix JA, Green CE, Robinson JD, Engelmann JM, Versace F, et al. An RCT with the combination of varenicline and bupropion for smoking cessation: clinical implications for front line use. Addiction. 2018.

52. Clavel F, Benhamou S, Companyhuertas A, Flamant R. Helping People to Stop Smoking - Randomized Comparison of Groups Being Treated with Acupuncture and Nicotine Gum with Control-Group. British Medical Journal. 1985;291(6508):1538-9.

53. Clavelchapelon F, Paoletti C, Benhamou S. A Randomized 2x2 Factorial Design to Evaluate Different Smoking Cessation Methods. Revue D Epidemiologie Et De Sante Publique. 1992;40(3):187-90.

54. Collins BN, Wileyto EP, Patterson F, Rukstalis M, Audrain-McGovern J, Kaufmann V, et al. Gender differences in smoking cessation in a placebo-controlled trial of bupropion with behavioral counseling. Nicotine Tob Res. 2004;6(1):27-37.

55. Cooney NL, Litt MD, Cooney JL, Pilkey DT, Steinberg HR, Oncken CA. Concurrent brief versus intensive smoking intervention during alcohol dependence treatment. Psychol Addict Behav. 2007;21(4):570-5.

56. Cooney NL, Cooney JL, Perry BL, Carbone M, Cohen EH, Steinberg HR, et al. Smoking cessation during alcohol treatment: a randomized trial of combination nicotine patch plus nicotine gum. Addiction. 2009;104(9):1588-96.

57. Cooper TV, Klesges RC, Debon MW, Zbikowski SM, Johnson KC, Clemens LH. A placebo controlled randomized trial of the effects of phenylpropanolamine and nicotine gum on cessation rates and postcessation weight gain in women. Addict Behav. 2005;30(1):61-75.

58. Cooperman NA, Lu SE, Richter KP, Bernstein SL, Williams JM. Pilot Study of a Tailored Smoking Cessation Intervention for Individuals in Treatment for Opioid Dependence. Nicotine Tob Res. 2018;20(9):1152-6.

59. Covey LS, Glassman AH, Jiang H, Fried J, Masmela J, LoDuca C, et al. A randomized trial of bupropion and/or nicotine gum as maintenance treatment for preventing smoking relapse. Addiction. 2007;102(8):1292-302.

60. Cox LS, Nollen NL, Mayo MS, Choi WS, Faseru B, Benowitz NL, et al. Bupropion for smoking cessation in African American light smokers: a randomized controlled trial. J Natl Cancer Inst. 2012;104(4):290-8.

61. Croghan GA, Sloan JA, Croghan IT, Novotny P, Hurt RD, DeKrey WL, et al. Comparison of nicotine patch alone versus nicotine nasal spray alone versus a combination for treating smokers: a minimal intervention, randomized multicenter trial in a nonspecialized setting. Nicotine Tob Res. 2003;5(2):181-7.

62. Cropsey KL, Clark CB, Zhang X, Hendricks PS, Jardin BF, Lahti AC. Race and Medication Adherence Moderate Cessation Outcomes in Criminal Justice Smokers. Am J Prev Med. 2015;49(3):335-44.

63. Cummings KM, Hyland A, Carlin-Menter S, Mahoney MC, Willett J, Juster HR. Costs of giving out free nicotine patches through a telephone quit line. J Public Health Manag Pract. 2011;17(3):E16-23.

64. Cummins SE, Gamst AC, Brandstein K, Seymann GB, Klonoff-Cohen H, Kirby CA, et al. Helping Hospitalized Smokers: A Factorial RCT of Nicotine Patches and Counseling. Am J Prev Med. 2016;51(4):578-86.

65. Cunningham JA, Kushnir V, Selby P, Tyndale RF, Zawertailo L, Leatherdale ST. Effect of Mailing Nicotine Patches on Tobacco Cessation Among Adult Smokers: A Randomized Clinical Trial. JAMA Intern Med. 2016;176(2):184-90.

66. Dale LC, Hurt RD, Offord KP, Lawson GM, Croghan IT, Schroeder DR. High-dose nicotine patch therapy. Percentage of replacement and smoking cessation. JAMA. 1995;274(17):1353-8.

67. Dale LC, Ebbert JO, Schroeder DR, Croghan IT, Rasmussen DF, Trautman JA, et al. Bupropion for the treatment of nicotine dependence in spit tobacco users: a pilot study. Nicotine Tob Res. 2002;4(3):267-74.

68. Dale LC, Ebbert JO, Glover ED, Croghan IT, Schroeder DR, Severson HH, et al. Bupropion SR for the treatment of smokeless tobacco use. Drug Alcohol Depend. 2007;90(1):56-63.

69. Dalsgareth OJ, Hansen NC, Soes-Petersen U, Evald T, Hoegholm A, Barber J, et al. A multicenter, randomized, double-blind, placebo-controlled, 6-month trial of bupropion hydrochloride sustained-release tablets as an aid to smoking cessation in hospital employees. Nicotine Tob Res. 2004;6(1):55-61.

70. Daughton DM, Heatley SA, Prendergast JJ, Causey D, Knowles M, Rolf CN, et al. Effect of Transdermal Nicotine Delivery as an Adjunct to Low-Intervention Smoking Cessation Therapy - a Randomized, Placebo-Controlled, Double-Blind-Study. Archives of Internal Medicine. 1991;151(4):749-52.

71. Daughton D, Susman J, Sitorius M, Belenky S, Millatmal T, Nowak R, et al. Transdermal nicotine therapy and primary care. Importance of counseling, demographic, and participant selection factors on 1-year quit rates. The Nebraska Primary Practice Smoking Cessation Trial Group. Arch Fam Med. 1998;7(5):425-30.

72. Daughton DM, Fortmann SP, Glover ED, Hatsukami DK, Heatley SA, Lichtenstein E, et al. The smoking cessation efficacy of varying doses of nicotine patch delivery systems 4 to 5 years post-quit day. Prev Med. 1999;28(2):113-8.

73. Dautzenberg B, Ruff F, Vaucher M, Maillon P, Jacob N, Kienzler JL, et al. First demonstration of the good efficacy/safety ratio of Nicotinell® 1mg Lozenge (NL 1mg), a new form of nicotine substitution, by randomised clinical trial. European Respiratory Journal. 2001;18 Suppl 33:12s.

74. Davidson M, Epstein M, Burt R, Schaefer C, Whitworth G, McDonald A. Efficacy and safety of an over-the-counter transdermal nicotine patch as an aid for smoking cessation. Archives of Family Medicine. 1998;7(6):569-74.

75. de Dios MA, Anderson BJ, Stanton C, Audet DA, Stein M. Project Impact: a pharmacotherapy pilot trial investigating the abstinence and treatment adherence of Latino light smokers. J Subst Abuse Treat. 2012;43(3):322-30.

76. Dogar O, Zahid R, Mansoor S, Kanaan M, Ahluwalia JS, Jawad M, et al. Varenicline versus placebo for waterpipe smoking cessation: a double-blind randomized controlled trial. Addiction. 2018;113(12):2290-9.

77. Ebbert JO, Dale LC, Patten CA, Croghan IT, Schroeder DR, Moyer TP, et al. Effect of high-dose nicotine patch therapy on tobacco withdrawal symptoms among smokeless tobacco users. Nicotine Tob Res. 2007;9(1):43-52.

78. Ebbert JO, Severson HH, Croghan IT, Danaher BG, Schroeder DR. A randomized clinical trial of nicotine lozenge for smokeless tobacco use. Nicotine Tob Res. 2009;11(12):1415-23.

79. Ebbert JO, Severson HH, Croghan IT, Danaher BG, Schroeder DR. A pilot study of mailed nicotine lozenges with assisted self-help for the treatment of smokeless tobacco users. Addict Behav. 2010;35(5):522-5.

80. Ebbert JO, Croghan IT, Severson HH, Schroeder DR, Hays JT. A pilot study of the efficacy of varenicline for the treatment of smokeless tobacco users in Midwestern United States. Nicotine Tob Res. 2011;13(9):820-6.

81. Ebbert JO, Croghan IT, Schroeder DR, Hurt RD. A randomized phase II clinical trial of high-dose nicotine patch therapy for smokeless tobacco users. Nicotine Tob Res. 2013;15(12):2037-44.

82. Ebbert JO, Hatsukami DK, Croghan IT, Schroeder DR, Allen SS, Hays JT, et al. Combination varenicline and bupropion SR for tobacco-dependence treatment in cigarette smokers: a randomized trial. JAMA. 2014;311(2):155-63.

83. Ebbert JO, Hughes JR, West RJ, Rennard SI, Russ C, McRae TD, et al. Effect of varenicline on smoking cessation through smoking reduction: a randomized clinical trial. JAMA. 2015;313(7):687-94.

84. Ebbert J, Croghan I, Hurt R, Schroeder D, Hays J. Varenicline for smoking cessation in light smokers2017; 18(10):[2031-5 pp.]. Available from: http://onlinelibrary.wiley.com/o/cochrane/clcentral/articles/505/CN-01245505/frame.html.

85. Ehrsam RE, Buhler A, Muller P, Mauli D, Schumacher PM, Howald H, et al. [Weaning of young smokers using a transdermal nicotine patch]. Schweiz Rundsch Med Prax. 1991;80(7):145-50.

86. Eisenberg MJ, Grandi SM, Gervais A, O'Loughlin J, Paradis G, Rinfret S, et al. Bupropion for smoking cessation in patients hospitalized with acute myocardial infarction: a randomized, placebo-controlled trial. J Am Coll Cardiol. 2013;61(5):524-32.

87. Eisenberg MJ, Windle SB, Roy N, Old W, Grondin FR, Bata I, et al. Varenicline for Smoking Cessation in Hospitalized Patients With Acute Coronary Syndrome. Circulation. 2016;133(1):21-30.

88. Ellerbeck EF, Nollen N, Hutcheson TD, Phadnis M, Fitzgerald SA, Vacek J, et al. Effect of Long-term Nicotine Replacement Therapy vs Standard Smoking Cessation for Smokers With Chronic Lung Disease: A Randomized Clinical Trial. JAMA Netw Open. 2018;1(5):e181843.

89. Etter JF, Laszlo E, Zellweger JP, Perrot C, Perneger TV. Nicotine replacement to reduce cigarette consumption in smokers who are unwilling to quit: a randomized trial. J Clin Psychopharmacol. 2002;22(5):487-95.

90. Etter JF, Huguelet P, Perneger TV, Cornuz J. Nicotine Gum Treatment Before Smoking Cessation A Randomized Trial. Archives of Internal Medicine. 2009;169(11):1028-34.

91. Evins AE, Mays VK, Rigotti NA, Tisdale T, Cather C, Goff DC. A pilot trial of bupropion added to cognitive behavioral therapy for smoking cessation in schizophrenia. Nicotine Tob Res. 2001;3(4):397-403.

92. Evins AE, Cather C, Deckersbach T, Freudenreich O, Culhane MA, Olm-Shipman CM, et al. A double-blind placebo-controlled trial of bupropion sustained-release for smoking cessation in schizophrenia. J Clin Psychopharmacol. 2005;25(3):218-25.

93. Evins AE, Cather C, Culhane MA, Birnbaum A, Horowitz J, Hsieh E, et al. A 12-week double-blind, placebo-controlled study of bupropion sr added to high-dose dual nicotine replacement therapy for smoking cessation or reduction in schizophrenia. J Clin Psychopharmacol. 2007;27(4):380-6.

94. Evins AE, Cather C, Pratt SA, Pachas GN, Hoeppner SS, Goff DC, et al. Maintenance treatment with varenicline for smoking cessation in patients with schizophrenia and bipolar disorder: a randomized clinical trial. JAMA. 2014;311(2):145-54.

95. Fagerstrom KO. A comparison of psychological and pharmacological treatment in smoking cessation. J Behav Med. 1982;5(3):343-51.

96. Fagerstrom KO. Effects of nicotine chewing gum and follow-up appointments in physician-based smoking cessation. Prev Med. 1984;13(5):517-27.

97. Fagerstrom K, Gilljam H, Metcalfe M, Tonstad S, Messig M. Stopping smokeless tobacco with varenicline: randomised double blind placebo controlled trial. BMJ. 2010;341:c6549.

98. Fee WM, Stewart MJ. A controlled trial of nicotine chewing gum in a smoking withdrawal clinic. Practitioner. 1982;226(1363):148-51.

99. Fernandez Arias IG, Garcia-Vera MP, Sanz J. The more psychology, the better: The efficacy of smoking cessation treatment using intensive cognitive-behavioral therapy versus a combination of nicotine patches plus intensive or less intensive cognitive-behavioral therapy: First prize of the 20th "Rafael Burgaleta" Applied Psychology Awards 2013. Clinica y Salud. 2014;25(1):1-10.

100. Ferry LH, Robbins AS, Scariati PD, Masterson A, Abbey DE, Burchette RJ. Enhancement of smoking cessation using the antidepressant bupropion hydrochloride [abstract 2670]. Circulation. 1992;86(4 Suppl 1):I–671.

101. Ferry LH, Burchette RJ. Efficacy of Bupropion for Smoking Cessation in Non Depressed Smokers. Journal of Addictive Diseases. 1994;13(4):249-.

102. Fiore MC, Kenford SL, Jorenby DE, Wetter DW, Smith SS, Baker TB. Two studies of the clinical effectiveness of the nicotine patch with different counseling treatments. Chest. 1994;105(2):524-33.

103. Fiore MC, McCarthy DE, Jackson TC, Zehner ME, Jorenby DE, Mielke M, et al. Integrating smoking cessation treatment into primary care: an effectiveness study. Prev Med. 2004;38(4):412-20.

104. Fortmann SP, Killen JD. Nicotine Gum and Self-Help Behavioral Treatment for Smoking Relapse Prevention - Results from a Trial Using Population-Based Recruitment. Journal of Consulting and Clinical Psychology. 1995;63(3):460-8.

105. Fossati R, Apolone G, Negri E, Compagnoni A, La Vecchia C, Mangano S, et al. A double-blind, placebo-controlled, randomized trial of bupropion for smoking cessation in primary care. Arch Intern Med. 2007;167(16):1791-7.

106. Fouz-Roson N, Montemayor-Rubio T, Almadana-Pacheco V, Montserrat-Garcia S, Gomez-Bastero AP, Romero-Munoz C, et al. Effect of 0.5 mg versus 1 mg varenicline for smoking cessation: a randomized controlled trial. Addiction. 2017;112(9):1610-9.

107. Gallagher SM, Penn PE, Schindler E, Layne W. A comparison of smoking cessation treatments for persons with schizophrenia and other serious mental illnesses. J Psychoactive Drugs. 2007;39(4):487-97.

108. Gariti P, Lynch K, Alterman A, Kampman K, Xie H, Varillo K. Comparing smoking treatment programs for lighter smokers with and without a history of heavier smoking. J Subst Abuse Treat. 2009;37(3):247-55.

109. Garvey AJ, Kinnunen T, Nordstrom BL, Utman CH, Doherty K, Rosner B, et al. Effects of nicotine gum dose by level of nicotine dependence. Nicotine Tob Res. 2000;2(1):53-63.

110. George TP, Vessicchio JC, Termine A, Bregartner TA, Feingold A, Rounsaville BJ, et al. A placebo controlled trial of bupropion for smoking cessation in schizophrenia. Biol Psychiatry. 2002;52(1):53-61.

111. George TP, Vessicchio JC, Sacco KA, Weinberger AH, Dudas MM, Allen TM, et al. A placebo-controlled trial of bupropion combined with nicotine patch for smoking cessation in schizophrenia. Biol Psychiatry. 2008;63(11):1092-6.

112. Gifford EV, Kohlenberg BS, Hayes SC, Antonuccio DO, Piasecki MM, Rasmussen-Hall ML, et al. Acceptance-based treatment for smoking cessation. Behavior Therapy. 2004;35(4):689-705.

113. Gilbert JR, Wilson DM, Best JA, Taylor DW, Lindsay EA, Singer J, et al. Smoking cessation in primary care. A randomized controlled trial of nicotine-bearing chewing gum. J Fam Pract. 1989;28(1):49-55.

114. Ginsberg D, Hall SM, Rosinski M. Partner support, psychological treatment, and nicotine gum in smoking treatment: an incremental study. Int J Addict. 1992;27(5):503-14.

115. Glavas D, Rumboldt M, Rumboldt Z. Smoking cessation with nicotine replacement therapy among health care workers: Randomized double-blind study. Croatian Medical Journal. 2003;44(2):219-24.

116. Glavas D, Rumboldt Z. [Smoking cessation using the transdermal nicotine system]. Lijec Vjesn. 2003;125(1-2):8-12.

117. GlaxoSmithKline. Efficacy and Safety Study of Nicotine Mint Lozenge (2mg and 4mg) in Smoking Cessation. https://ClinicalTrials.gov/show/NCT00985985; 2009.

118. Glover ED, Glover PN, Franzon M, Sullivan CR, Cerullo CC, Howell RM, et al. A comparison of a nicotine sublingual tablet and placebo for smoking cessation. Nicotine Tob Res. 2002;4(4):441-50.

119. Goldstein MG, Niaura R, Follick MJ, Abrams DB. Effects of behavioral skills training and schedule of nicotine gum administration on smoking cessation. Am J Psychiatry. 1989;146(1):56-60.

120. Gonzales DH, Nides MA, Ferry LH, Kustra RP, Jamerson BD, Segall N, et al. Bupropion SR as an aid to smoking cessation in smokers treated previously with bupropion: a randomized placebo-controlled study. Clin Pharmacol Ther. 2001;69(6):438-44.

121. Gonzales D, Rennard SI, Nides M, Oncken C, Azoulay S, Billing CB, et al. Varenicline, an alpha4beta2 nicotinic acetylcholine receptor partial agonist, vs sustained-release bupropion and placebo for smoking cessation: a randomized controlled trial. JAMA. 2006;296(1):47-55.

122. Gonzales D, Hajek P, Pliamm L, Nackaerts K, Tseng LJ, McRae TD, et al. Retreatment with varenicline for smoking cessation in smokers who have previously taken varenicline: a randomized, placebo-controlled trial. Clin Pharmacol Ther. 2014;96(3):390-6.

123. Gorecka D, Bednarek M, Nowinski A, Puscinska E, Goljan-Geremek A, Zielinski J. [Effect of treatment for nicotine dependence in patients with COPD]. Pneumonol Alergol Pol. 2003;71(9-10):411-7.

124. Gourlay SG, Forbes A, Marriner T, Pethica D, McNeil JJ. Double blind trial of repeated treatment with transdermal nicotine for relapsed smokers. BMJ. 1995;311(7001):363-6.

125. Graham AL, Papandonatos GD, Cha S, Erar B, Amato MS, Cobb NK, et al. Improving Adherence to Smoking Cessation Treatment: Intervention Effects in a Web-Based Randomized Trial. Nicotine Tob Res. 2017;19(3):324-32.

126. Grant KM, Kelley SS, Smith LM, Agrawal S, Meyer JR, Romberger DJ. Bupropion and nicotine patch as smoking cessation aids in alcoholics. Alcohol. 2007;41(5):381-91.

127. Gross J, Johnson J, Sigler L, Stitzer ML. Dose effects of nicotine gum. Addict Behav. 1995;20(3):371-81.

128. Haas JS, Linder JA, Park ER, Gonzalez I, Rigotti NA, Klinger EV, et al. Proactive tobacco cessation outreach to smokers of low socioeconomic status: a randomized clinical trial. JAMA Intern Med. 2015;175(2):218-26.

129. Haggstram FM, Chatkin JM, Sussenbach-Vaz E, Cesari DH, Fam CF, Fritscher CC. A controlled trial of nortriptyline, sustained-release bupropion and placebo for smoking cessation: preliminary results. Pulm Pharmacol Ther. 2006;19(3):205-9.

130. Hajek P, Phillips-Waller A, Przulj D, Pesola F, Myers Smith K, Bisal N, et al. A Randomized Trial of E-Cigarettes versus Nicotine-Replacement Therapy. N Engl J Med. 2019;380(7):629-37.

131. Hall SM, Tunstall C, Rugg D, Jones RT, Benowitz N. Nicotine gum and behavioral treatment in smoking cessation. J Consult Clin Psychol. 1985;53(2):256-8.

132. Hall SM, Tunstall CD, Ginsberg D, Benowitz NL, Jones RT. Nicotine gum and behavioral treatment: a placebo controlled trial. J Consult Clin Psychol. 1987;55(4):603-5.

133. Hall SM, Munoz RF, Reus VI, Sees KL, Duncan C, Humfleet GL, et al. Mood management and nicotine gum in smoking treatment: a therapeutic contact and placebo-controlled study. J Consult Clin Psychol. 1996;64(5):1003-9.

134. Hall SM, Humfleet GL, Reus VI, Munoz RF, Hartz DT, Maude-Griffin R. Psychological intervention and antidepressant treatment in smoking cessation. Arch Gen Psychiatry. 2002;59(10):930-6.

135. Hall SM, Tsoh JY, Prochaska JJ, Eisendrath S, Rossi JS, Redding CA, et al. Treatment for cigarette smoking among depressed mental health outpatients: a randomized clinical trial. Am J Public Health. 2006;96(10):1808-14.

136. Hall SM, Humfleet GL, Munoz RF, Reus VI, Robbins JA, Prochaska JJ. Extended treatment of older cigarette smokers. Addiction. 2009;104(6):1043-52.

137. Hall SM, Humfleet GL, Munoz RF, Reus VI, Prochaska JJ, Robbins JA. Using extended cognitive behavioral treatment and medication to treat dependent smokers. Am J Public Health. 2011;101(12):2349-56.

138. Halpern SD, Harhay MO, Saulsgiver K, Brophy C, Troxel AB, Volpp KG. A Pragmatic Trial of E-Cigarettes, Incentives, and Drugs for Smoking Cessation. N Engl J Med. 2018;378(24):2302-10.

139. Hand S, Edwards S, Campbell IA, Cannings R. Controlled trial of three weeks nicotine replacement treatment in hospital patients also given advice and support. Thorax. 2002;57(8):715-8.

140. Hanioka T, Ojima M, Tanaka H, Naito M, Hamajima N, Matsuse R. Intensive smoking-cessation intervention in the dental setting. J Dent Res. 2010;89(1):66-70.

141. Harackiewicz JM, Blair LW, Sansone C, Epstein JA, Stuchell RN. Nicotine gum and self-help manuals in smoking cessation: an evaluation in a medical context. Addict Behav. 1988;13(4):319-30.

142. Hatsukami D, Jensen J, Allen S, Grillo M, Bliss R. Effects of behavioral and pharmacological treatment on smokeless tobacco users. J Consult Clin Psychol. 1996;64(1):153-61.

143. Hatsukami DK, Grillo M, Boyle R, Allen S, Jensen J, Bliss R, et al. Treatment of spit tobacco users with transdermal nicotine system and mint snuff. J Consult Clin Psychol. 2000;68(2):241-9.

144. Hatsukami DK, Rennard S, Patel MK, Kotlyar M, Malcolm R, Nides MA, et al. Effects of sustained-release bupropion among persons interested in reducing but not quitting smoking. Am J Med. 2004;116(3):151-7.

145. Hays JT, Croghan IT, Schroeder DR, Offord KP, Hurt RD, Wolter TD, et al. Over-the-counter nicotine patch therapy for smoking cessation: results from randomized, double-blind, placebo-controlled, and open label trials. Am J Public Health. 1999;89(11):1701-7.

146. Hays JT, Hurt RD, Rigotti NA, Niaura R, Gonzales D, Durcan MJ, et al. Sustained-release bupropion for pharmacologic relapse prevention after smoking cessation. a randomized, controlled trial. Ann Intern Med. 2001;135(6):423-33.

147. Hays JT, Hurt RD, Decker PA, Croghan IT, Offord KP, Patten CA. A randomized, controlled trial of bupropion sustained-release for preventing tobacco relapse in recovering alcoholics. Nicotine Tob Res. 2009;11(7):859-67.

148. Herrera N, Franco R, Herrera L, Partidas A, Rolando R, Fagerstrom KO. Nicotine gum, 2 and 4 mg, for nicotine dependence. A double-blind placebo-controlled trial within a behavior modification support program. Chest. 1995;108(2):447-51.

149. Hertzberg MA, Moore SD, Feldman ME, Beckham JC. A preliminary study of bupropion sustained-release for smoking cessation in patients with chronic posttraumatic stress disorder. J Clin Psychopharmacol. 2001;21(1):94-8.

150. Heydari G, Talischi F, Tafti SF, Masjedi MR. Quitting smoking with varenicline: parallel, randomised efficacy trial in Iran. Int J Tuberc Lung Dis. 2012;16(2):268-72.

151. Heydari G, Talischi F, Batmanghelidj E, Pajooh M, Boroomand A, Zamani M, et al. Dual addictions, parallel treatments: Nicotine replacement therapy for patients receiving methadone treatment in the Islamic Republic of Iran2013. Available from: http://onlinelibrary.wiley.com/o/cochrane/clcentral/articles/288/CN-00911288/frame.html.

152. Hilberink SR, Jacobs JE, Breteler MH, de Vries H, Grol RP. General practice counseling for patients with chronic obstructive pulmonary disease to quit smoking: impact after 1 year of two complex interventions. Patient Educ Couns. 2011;83(1):120-4.

153. Hilleman DE, Mohiuddin SM, Delcore MG. Comparison of fixed-dose transdermal nicotine, tapered-dose transdermal nicotine, and buspirone in smoking cessation. J Clin Pharmacol. 1994;34(3):222-4.

154. Hjalmarson AI. Effect of nicotine chewing gum in smoking cessation. A randomized, placebo-controlled, double-blind study. JAMA. 1984;252(20):2835-8.

155. Hjalmarson A, Franzon M, Westin A, Wiklund O. Effect of nicotine nasal spray on smoking cessation. A randomized, placebo-controlled, double-blind study. Arch Intern Med. 1994;154(22):2567-72.

156. Hjalmarson A, Nilsson F, Sjostrom L, Wiklund O. The nicotine inhaler in smoking cessation. Arch Intern Med. 1997;157(15):1721-8.

157. Hollis JF, McAfee TA, Fellows JL, Zbikowski SM, Stark M, Riedlinger K. The effectiveness and cost effectiveness of telephone counselling and the nicotine patch in a state tobacco quitline. Tob Control. 2007;16 Suppl 1(SUPPL. 1):i53-9.

158. Holt S, Timu-Parata C, Ryder-Lewis S, Weatherall M, Beasley R. Efficacy of bupropion in the indigenous Maori population in New Zealand. Thorax. 2005;60(2):120-3.

159. Dale Horst W, Klein MW, Williams D, Werder SF. Extended use of nicotine replacement therapy to maintain smoking cessation in persons with schizophrenia. Neuropsychiatr Dis Treat. 2005;1(4):349-55.

160. Howard-Pitney B, Killen JD, Fortmann SP. Quitting chew: results from a randomized trial using nicotine patches. Exp Clin Psychopharmacol. 1999;7(4):362-71.

161. Huber D. Combined and separate treatment effects of nicotine chewing gum and self-control method. Pharmacopsychiatry. 1988;21(6):461-2.

162. Hughes JR, Gust SW, Keenan RM, Fenwick JW, Healey ML. Nicotine vs placebo gum in general medical practice. JAMA. 1989;261(9):1300-5.

163. Hughes JR, Gust SW, Keenan RM, Fenwick JW. Effect of Dose on Nicotines Reinforcing, Withdrawal-Suppression and Self-Reported Effects. Journal of Pharmacology and Experimental Therapeutics. 1990;252(3):1175-83.

164. Hughes JR, Lesmes GR, Hatsukami DK, Richmond RL, Lichtenstein E, Jorenby DE, et al. Are higher doses of nicotine replacement more effective for smoking cessation? Nicotine Tob Res. 1999;1(2):169-74.

165. Hughes JR, Novy P, Hatsukami DK, Jensen J, Callas PW. Efficacy of nicotine patch in smokers with a history of alcoholism. Alcohol Clin Exp Res. 2003;27(6):946-54.

166. Hughes JR, Solomon LJ, Livingston AE, Callas PW, Peters EN. A randomized, controlled trial of NRT-aided gradual vs. abrupt cessation in smokers actively trying to quit. Drug Alcohol Depend. 2010;111(1-2):105-13.

167. Hughes JR, Rennard SI, Fingar JR, Talbot SK, Callas PW, Fagerstrom KO. Efficacy of varenicline to prompt quit attempts in smokers not currently trying to quit: a randomized placebo-controlled trial. Nicotine Tob Res. 2011;13(10):955-64.

168. Hurt RD, Lauger GG, Offord KP, Kottke TE, Dale LC. Nicotine-Replacement Therapy with Use of a Transdermal Nicotine Patch - a Randomized Double-Blind Placebo-Controlled Trial. Mayo Clinic Proceedings. 1990;65(12):1529-37.

169. Hurt RD, Dale LC, Fredrickson PA, Caldwell CC, Lee GA, Offord KP, et al. Nicotine patch therapy for smoking cessation combined with physician advice and nurse follow-up. One-year outcome and percentage of nicotine replacement. JAMA. 1994;271(8):595-600.

170. Hurt RD, Sachs DP, Glover ED, Offord KP, Johnston JA, Dale LC, et al. A comparison of sustained-release bupropion and placebo for smoking cessation. N Engl J Med. 1997;337(17):1195-202.

171. Hurt RD, Krook JE, Croghan IT, Loprinzi CL, Sloan JA, Novotny PJ, et al. Nicotine patch therapy based on smoking rate followed by bupropion for prevention of relapse to smoking. J Clin Oncol. 2003;21(5):914-20.

172. Hurt RT, Ebbert JO, Croghan IT, Schroeder DR, Hurt RD, Hays JT. Varenicline for tobacco-dependence treatment in alcohol-dependent smokers: A randomized controlled trial. Drug Alcohol Depend. 2018;184:12-7.

173. Randomised trial of nicotine patches in general practice: results at one year. Imperial Cancer Research Fund General Practice Research Group. BMJ. 1994;308(6942):1476-7.

174. Ikonomidis I, Marinou M, Vlastos D, Kourea K, Andreadou I, Liarakos N, et al. Effects of varenicline and nicotine replacement therapy on arterial elasticity, endothelial glycocalyx and oxidative stress during a 3-month smoking cessation program. Atherosclerosis. 2017;262:123-30.

175. Jamrozik K, Fowler G, Vessey M, Wald N. Placebo controlled trial of nicotine chewing gum in general practice. Br Med J (Clin Res Ed). 1984;289(6448):794-7.

176. Jarvis MJ, Raw M, Russell MAH, Feyerabend C. Randomized Controlled Trial of Nicotine Chewing-Gum. British Medical Journal. 1982;285(6341):537-40.

177. Jensen EJ, Schmidt E, Pedersen B, Dahl R. The effect of nicotine, silver acetate, and placebo chewing gum on the cessation of smoking. The influence of smoking type and nicotine dependence. Int J Addict. 1991;26(11):1223-31.

178. Johns DA. Randomised controlled trial comparing nicotine replacement therapy (NRT) plus brief counselling and brief counselling alone on smoking cessation in patients prone to lung cancer using a breath carbon monoxide (CO) monitor. Annals of Oncology. 2016;27:ix177.

179. Johns D. Randomised controlled trial comparing nicotine replacement therapy (nrt) and counselling on smoking cessation in patients prone to lung cancer using bed font micro-smokerlyzer. Support Care Cancer. 2017;25 (2 Supplement 1):21-266.

180. Johns D. Randomised controlled trial comparing varenicline plus counselling and brief counselling alone on smoking cessation in patients prone to lung cancer using carbon monoxide moniter. Support Care Cancer. 2017;25 (2 Supplement 1):21-266.

181. Jorenby DE, Smith SS, Fiore MC, Hurt RD, Offord KP, Croghan IT, et al. Varying nicotine patch dose and type of smoking cessation counseling. JAMA. 1995;274(17):1347-52.

182. Jorenby DE, Leischow SJ, Nides MA, Rennard SI, Johnston JA, Hughes AR, et al. A controlled trial of sustained-release bupropion, a nicotine patch, or both for smoking cessation. N Engl J Med. 1999;340(9):685-91.

183. Jorenby DE, Hays JT, Rigotti NA, Azoulay S, Watsky EJ, Williams KE, et al. Efficacy of varenicline, an alpha4beta2 nicotinic acetylcholine receptor partial agonist, vs placebo or sustained-release bupropion for smoking cessation: a randomized controlled trial. JAMA. 2006;296(1):56-63.

184. Joseph AM, Norman SM, Ferry LH, Prochazka AV, Westman EC, Steele BG, et al. The safety of transdermal nicotine as an aid to smoking cessation in patients with cardiac disease. N Engl J Med. 1996;335(24):1792-8.

185. Joseph AM, Willenbring ML, Nugent SM, Nelson DB. A randomized trial of concurrent versus delayed smoking intervention for patients in alcohol dependence treatment. J Stud Alcohol. 2004;65(6):681-91.

186. Joseph AM, Fu SS, Lindgren B, Rothman AJ, Kodl M, Lando H, et al. Chronic disease management for tobacco dependence: a randomized, controlled trial. Arch Intern Med. 2011;171(21):1894-900.

187. Joyce GF, Niaura R, Maglione M, Mongoven J, Larson-Rotter C, Coan J, et al. The effectiveness of covering smoking cessation services for medicare beneficiaries. Health Serv Res. 2008;43(6):2106-23.

188. Kalman D, Kahler CW, Garvey AJ, Monti PM. High-dose nicotine patch therapy for smokers with a history of alcohol dependence: 36-week outcomes. J Subst Abuse Treat. 2006;30(3):213-7.

189. Kalman D, Herz L, Monti P, Kahler CW, Mooney M, Rodrigues S, et al. Incremental efficacy of adding bupropion to the nicotine patch for smoking cessation in smokers with a recent history of alcohol dependence: results from a randomized, double-blind, placebo-controlled study. Drug Alcohol Depend. 2011;118(2-3):111-8.

190. Killen JD, Maccoby N, Taylor CB. Nicotine Gum and Self-Regulation Training in Smoking Relapse Prevention. Behavior Therapy. 1984;15(3):234-48.

191. Killen JD, Fortmann SP, Newman B, Varady A. Evaluation of a treatment approach combining nicotine gum with self-guided behavioral treatments for smoking relapse prevention. J Consult Clin Psychol. 1990;58(1):85-92.

192. Killen JD, Fortmann SP, Davis L, Varady A. Nicotine patch and self-help video for cigarette smoking cessation. J Consult Clin Psychol. 1997;65(4):663-72.

193. Killen JD, Fortmann SP, Davis L, Strausberg L, Varady A. Do heavy smokers benefit from higher dose nicotine patch therapy? Experimental and Clinical Psychopharmacology. 1999;7(3):226-33.

194. Killen JD, Fortmann SP, Murphy GM, Jr., Hayward C, Arredondo C, Cromp D, et al. Extended treatment with bupropion SR for cigarette smoking cessation. J Consult Clin Psychol. 2006;74(2):286-94.

195. Klesges RC, Ebbert JO, Talcott GW, Thomas F, Richey PA, Womack C, et al. Efficacy of a Tobacco Quitline in Active Duty Military and TRICARE Beneficiaries: A Randomized Trial. Mil Med. 2015;180(8):917-25.

196. Koegelenberg CF, Noor F, Bateman ED, van Zyl-Smit RN, Bruning A, O'Brien JA, et al. Efficacy of varenicline combined with nicotine replacement therapy vs varenicline alone for smoking cessation: a randomized clinical trial. JAMA. 2014;312(2):155-61.

197. Kornitzer M, Kittel F, Dramaix M, Bourdoux P. A Double-Blind-Study of 2 Mg Versus 4 Mg Nicotine-Gum in an Industrial-Setting. Journal of Psychosomatic Research. 1987;31(2):171-6.

198. Kornitzer M, Boutsen M, Dramaix M, Thijs J, Gustavsson G. Combined use of nicotine patch and gum in smoking cessation: a placebo-controlled clinical trial. Prev Med. 1995;24(1):41-7.

199. Kralikova E, Kozak J, Rasmussen T, Cort N. The clinical benefits of NRT-supported smoking reduction. Nicotine Tob Res. 2002;4(243):20.

200. Krupski L, Cummings KM, Hyland A, Mahoney MC, Toll BA, Carpenter MJ, et al. Cost and Effectiveness of Combination Nicotine Replacement Therapy Among Heavy Smokers Contacting a Quitline. Journal of Smoking Cessation. 2016;11(1):50-9.

201. Lee SM, Landry J, Jones PM, Buhrmann O, Morley-Forster P. The effectiveness of a perioperative smoking cessation program: a randomized clinical trial. Anesth Analg. 2013;117(3):605-13.

202. Lee SM, Tenney R, Wallace AW, Arjomandi M. E-cigarettes versus nicotine patches for perioperative smoking cessation: a pilot randomized trial. PeerJ. 2018;6:e5609.

203. Leischow SJ, Nilsson F, Franzon M, Hill A, Otte P, Merikle EP. Efficacy of the nicotine inhaler as an adjunct to smoking cessation. American Journal of Health Behavior. 1996;20(5):364-71.

204. Leischow SJ, Muramoto ML, Cook GN, Merikle EP, Castellini SM, Otte PS. OTC nicotine patch: Effectiveness alone and with brief physician intervention. American Journal of Health Behavior. 1999;23(1):61-9.

205. Leischow SJ, Ranger-Moore J, Muramoto ML, Matthews E. Effectiveness of the nicotine inhaler for smoking cessation in an OTC setting. Am J Health Behav. 2004;28(4):291-301.

206. Lerman C, Kaufmann V, Rukstalis M, Patterson F, Perkins K, Audrain-McGovern J, et al. Individualizing nicotine replacement therapy for the treatment of tobacco dependence: a randomized trial. Ann Intern Med. 2004;140(6):426-33.

207. Lerman C, Schnoll RA, Hawk LW, Jr., Cinciripini P, George TP, Wileyto EP, et al. Use of the nicotine metabolite ratio as a genetically informed biomarker of response to nicotine patch or varenicline for smoking cessation: a randomised, double-blind placebo-controlled trial. Lancet Respir Med. 2015;3(2):131-8.

208. Levine MD, Perkins KA, Kalarchian MA, Cheng Y, Houck PR, Slane JD, et al. Bupropion and cognitive behavioral therapy for weight-concerned women smokers. Arch Intern Med. 2010;170(6):543-50.

209. Lewis SF, Piasecki TM, Fiore MC, Anderson JE, Baker TB. Transdermal nicotine replacement for hospitalized patients: a randomized clinical trial. Prev Med. 1998;27(2):296-303.

210. Littlewood RA, Claus ED, Wilcox CE, Mickey J, Arenella PB, Bryan AD, et al. Moderators of smoking cessation outcomes in a randomized-controlled trial of varenicline versus placebo. Psychopharmacology (Berl). 2017;234(23-24):3417-29.

211. Llivina TS, Tuya DM, Quintana JG, Torres CI, Barrera EC, Saez CM, et al. Smoking Cessation - Effectiveness of Nicotine Containing Chewing Gum - a Double-Blind Trial. Medicina Clinica. 1988;90(16):646-50.

212. Lloyd-Richardson EE, Stanton CA, Papandonatos GD, Shadel WG, Stein M, Tashima K, et al. Motivation and patch treatment for HIV+ smokers: a randomized controlled trial. Addiction. 2009;104(11):1891-900.

213. Malcolm RE, Sillett RW, Turner JA, Ball KP. The use of nicotine chewing gum as an aid to stopping smoking. Psychopharmacology (Berl). 1980;70(3):295-6.

214. Martin JE, Calfas KJ, Patten CA, Polarek M, Hofstetter CR, Noto J, et al. Prospective evaluation of three smoking interventions in 205 recovering alcoholics: one-year results of Project SCRAP-Tobacco. J Consult Clin Psychol. 1997;65(1):190-4.

215. McAfee TA, Bush T, Deprey TM, Mahoney LD, Zbikowski SM, Fellows JL, et al. Nicotine patches and uninsured quitline callers: a randomized trial of two versus eight weeks (Provisional abstract)2008; 35(2):[103-10 pp.]. Available from: http://onlinelibrary.wiley.com/o/cochrane/cleed/articles/NHSEED-22008101391/frame.html.

216. McCarthy DE, Piasecki TM, Lawrence DL, Jorenby DE, Shiffman S, Fiore MC, et al. A randomized controlled clinical trial of bupropion SR and individual smoking cessation counseling. Nicotine Tob Res. 2008;10(4):717-29.

217. Mcgovern PG, Lando HA. An Assessment of Nicotine Gum as an Adjunct to Freedom from Smoking Cessation Clinics. Addictive Behaviors. 1992;17(2):137-47.

218. Mercie P, Arsandaux J, Katlama C, Ferret S, Beuscart A, Spadone C, et al. Efficacy and safety of varenicline for smoking cessation in people living with HIV in France (ANRS 144 Inter-ACTIV): a randomised controlled phase 3 clinical trial. Lancet Hiv. 2018;5(3):E126-E35.

219. Molyneux A, Lewis S, Leivers U, Anderton A, Antoniak M, Brackenridge A, et al. Clinical trial comparing nicotine replacement therapy (NRT) plus brief counselling, brief counselling alone, and minimal intervention on smoking cessation in hospital inpatients. Thorax. 2003;58(6):484-8.

220. Mori T STYGNMHT. A clinical trial of nicotine chewing gum for smoking cessation [abstract 428]. 1992.

221. Myles PS, Leslie K, Angliss M, Mezzavia P, Lee L. Effectiveness of bupropion as an aid to stopping smoking before elective surgery: a randomised controlled trial. Anaesthesia. 2004;59(11):1053-8.

222. Myung SK, Seo HG, Park S, Kim Y, Kim DJ, Lee DH, et al. Sociodemographic and smoking behavioral predictors associated with smoking cessation according to follow-up periods: a randomized, double-blind, placebo-controlled trial of transdermal nicotine patches. J Korean Med Sci. 2007;22(6):1065-70.

223. Nahvi S, Ning Y, Segal KS, Richter KP, Arnsten JH. Varenicline efficacy and safety among methadone maintained smokers: a randomized placebo-controlled trial. Addiction. 2014;109(9):1554-63.

224. Nakamura M, J. S, A. O, M. M, A. M, S. E. Effect of nicotine chewing gun in smoking cessation classes. The Global War. 1990:665–7.

225. Nakamura M, Oshima A, Fujimoto Y, Maruyama N, Ishibashi T, Reeves KR. Efficacy and tolerability of varenicline, an alpha4beta2 nicotinic acetylcholine receptor partial agonist, in a 12-week, randomized, placebo-controlled, dose-response study with 40-week follow-up for smoking cessation in Japanese smokers. Clin Ther. 2007;29(6):1040-56.

226. Fox Chase Cancer Center, National Cancer Institute. Counseling and Nicotine Replacement Therapy in Helping Adult Smokers Quit Smoking. https://ClinicalTrials.gov/show/NCT00365508; 2006.

227. Pfizer. Long-term Varenicline Treatment for Smoking Cessation. University of Wisconsin, Madison: https://ClinicalTrials.gov/show/NCT00828113; 2009.

228. Nebot M, Cabezas C. Does nurse counseling or offer of nicotine gum improve the effectiveness of physician smoking-cessation advice? Fam Pract Res J. 1992;12(3):263-70.

229. Niaura R, Goldstein MG, Abrams DB. Matching high- and low-dependence smokers to self-help treatment with or without nicotine replacement. Prev Med. 1994;23(1):70-7.

230. Niaura R, Abrams DB, Shadel WG, Rohsenow DJ, Monti PM, Sirota AD. Cue exposure treatment for smoking relapse prevention: a controlled clinical trial. Addiction. 1999;94(5):685-95.

231. Niaura R, Hays JT, Jorenby DE, Leone FT, Pappas JE, Reeves KR, et al. The efficacy and safety of varenicline for smoking cessation using a flexible dosing strategy in adult smokers: a randomized controlled trial. Curr Med Res Opin. 2008;24(7):1931-41.

232. Nides M, Oncken C, Gonzales D, Rennard S, Watsky EJ, Anziano R, et al. Smoking cessation with varenicline, a selective alpha4beta2 nicotinic receptor partial agonist: results from a 7-week, randomized, placebo- and bupropion-controlled trial with 1-year follow-up. Arch Intern Med. 2006;166(15):1561-8.

233. Nides M, Danielsson T, Saunders F, Perfekt R, Kapikian R, Solla J, et al. Efficacy and safety of a nicotine mouth spray for smoking cessation; a randomized, multicenter, controlled study in a naturalistic setting. Nicotine Tob Res. 2018;18:18.

234. Ockene JK, Kristeller J, Goldberg R, Amick TL, Pekow PS, Hosmer D, et al. Increasing the efficacy of physician-delivered smoking interventions: a randomized clinical trial. J Gen Intern Med. 1991;6(1):1-8.

235. Okuyemi KS, James AS, Mayo MS, Nollen N, Catley D, Choi WS, et al. Pathways to health: a cluster randomized trial of nicotine gum and motivational interviewing for smoking cessation in low-income housing. Health Educ Behav. 2007;34(1):43-54.

236. Oncken C, Gonzales D, Nides M, Rennard S, Watsky E, Billing CB, et al. Efficacy and safety of the novel selective nicotinic acetylcholine receptor partial agonist, varenicline, for smoking cessation. Arch Intern Med. 2006;166(15):1571-7.

237. Oncken C, Cooney J, Feinn R, Lando H, Kranzler HR. Transdermal nicotine for smoking cessation in postmenopausal women. Addict Behav. 2007;32(2):296-309.

238. Ortega F, Vellisco A, Marquez E, Lopez-Campos JL, Rodriguez A, de los Angeles Sanchez M, et al. Effectiveness of a cognitive orientation program with and without nicotine replacement therapy in stopping smoking in hospitalised patients. Arch Bronconeumol. 2011;47(1):3-9.

239. Pack QR, Jorenby DE, Fiore MC, Jackson T, Weston P, Piper ME, et al. A comparison of the nicotine lozenge and nicotine gum: an effectiveness randomized controlled trial. WMJ. 2008;107(5):237-43.

240. Page AR, Walters DJ, Schlegel RP, Best JA. Smoking cessation in family practice: the effects of advice and nicotine chewing gum prescription. Addict Behav. 1986;11(4):443-6.

241. Paoletti P, Fornai E, Maggiorelli F, Puntoni R, Viegi G, Carrozzi L, et al. Importance of baseline cotinine plasma values in smoking cessation: results from a double-blind study with nicotine patch. Eur Respir J. 1996;9(4):643-51.

242. Perng RP, Hsieh WC, Chen YM, Lu CC, Chiang SJ. Randomized, double-blind, placebo-controlled study of transdermal nicotine patch for smoking cessation. J Formos Med Assoc. 1998;97(8):547-51.

243. Piper ME, Federman EB, McCarthy DE, Bolt DM, Smith SS, Fiore MC, et al. Efficacy of bupropion alone and in combination with nicotine gum. Nicotine Tob Res. 2007;9(9):947-54.

244. Piper ME, Smith SS, Schlam TR, Fiore MC, Jorenby DE, Fraser D, et al. A randomized placebo-controlled clinical trial of 5 smoking cessation pharmacotherapies. Arch Gen Psychiatry. 2009;66(11):1253-62.

245. Piper ME, Cook JW, Schlam TR, Jorenby DE, Smith SS, Collins LM, et al. A Randomized Controlled Trial of an Optimized Smoking Treatment Delivered in Primary Care. Ann Behav Med. 2018;52(10):854-64.

246. Pirie PL, McBride CM, Hellerstedt W, Jeffery RW, Hatsukami D, Allen S, et al. Smoking cessation in women concerned about weight. Am J Public Health. 1992;82(9):1238-43.

247. Planer D, Lev I, Elitzur Y, Sharon N, Ouzan E, Pugatsch T, et al. Bupropion for smoking cessation in patients with acute coronary syndrome. Arch Intern Med. 2011;171(12):1055-60.

248. Prapavessis H, Cameron L, Baldi JC, Robinson S, Borrie K, Harper T, et al. The effects of exercise and nicotine replacement therapy on smoking rates in women. Addict Behav. 2007;32(7):1416-32.

249. Puska P, Bjorkqvist S, Koskela K. Nicotine-containing chewing gum in smoking cessation: a double blind trial with half year follow-up. Addict Behav. 1979;4(2):141-6.

250. Puska P, Korhonen HJ, Vartiainen E, Urjanheimo EL, Gustavsson G, Westin A. Combined use of nicotine patch and gum compared with gum alone in smoking cessation - a clinical trial in North Karelia. Tobacco Control. 1995;4(3):231-5.

251. Quilez Garcia C, Hernando Arizaleta L, Rubio Diaz A, Granero Fernandez EJ, Vila Coll MA, Estruch Riba J. [Double-blind study of the efficacy of nicotine chewing gum for smoking cessation in the primary care setting]. Aten Primaria. 1989;6(10):719-26.

252. Ramon JM, Morchon S, Baena A, Masuet-Aumatell C. Combining varenicline and nicotine patches: a randomized controlled trial study in smoking cessation. BMC Med. 2014;12(172):172.

253. Ratner PA, Johnson JL, Richardson CG, Bottorff JL, Moffat B, Mackay M, et al. Efficacy of a smoking-cessation intervention for elective-surgical patients. Res Nurs Health. 2004;27(3):148-61.

254. Reid R, Pipe A, Higginson L, Johnson K, D'Angelo MS, Cooke D, et al. Stepped care approach to smoking cessation in patients hospitalized for coronary artery disease. J Cardiopulm Rehabil. 2003;23(3):176-82.

255. Reid MS, Fallon B, Sonne S, Flammino F, Nunes EV, Jiang H, et al. Smoking cessation treatment in community-based substance abuse rehabilitation programs. J Subst Abuse Treat. 2008;35(1):68-77.

256. Rennard SI, Glover ED, Leischow S, Daughton DM, Glover PN, Muramoto M, et al. Efficacy of the nicotine inhaler in smoking reduction: A double-blind, randomized trial. Nicotine Tob Res. 2006;8(4):555-64.

257. Rennard S, Hughes J, Cinciripini PM, Kralikova E, Raupach T, Arteaga C, et al. A randomized placebo-controlled trial of varenicline for smoking cessation allowing flexible quit dates. Nicotine Tob Res. 2012;14(3):343-50.

258. Richmond RL, Makinson RJ, Kehoe LA, Giugni AA, Webster IW. One-year evaluation of three smoking cessation interventions administered by general practitioners. Addict Behav. 1993;18(2):187-99.

259. Richmond RL, Harris K, Dealmeidaneto A. The Transdermal Nicotine Patch - Results of a Randomized Placebo-Controlled Trial. Medical Journal of Australia. 1994;161(2):130-&.

260. Rigotti NA, Thorndike AN, Regan S, McKool K, Pasternak RC, Chang Y, et al. Bupropion for smokers hospitalized with acute cardiovascular disease. Am J Med. 2006;119(12):1080-7.

261. Rigotti NA, Pipe AL, Benowitz NL, Arteaga C, Garza D, Tonstad S. Efficacy and safety of varenicline for smoking cessation in patients with cardiovascular disease: a randomized trial. Circulation. 2010;121(2):221-9.

262. Rodriguez-Artalejo F, Lafuente Urdinguio P, Guallar-Castillon P, Garteizaurrekoa Dublang P, Sainz Martinez O, Diez Azcarate JI, et al. One year effectiveness of an individualised smoking cessation intervention at the workplace: a randomised controlled trial. Occup Environ Med. 2003;60(5):358-63.

263. Rohsenow DJ, Tidey JW, Martin RA, Colby SM, Swift RM, Leggio L, et al. Varenicline versus nicotine patch with brief advice for smokers with substance use disorders with or without depression: effects on smoking, substance use and depressive symptoms. Addiction. 2017;112(10):1808-20.

264. Rose JE, Behm FM. Adapting smoking cessation treatment according to initial response to precessation nicotine patch. Am J Psychiatry. 2013;170(8):860-7.

265. Roto P OASKJKPR. Nicotine gum and withdrawal from smoking. Suomen Laakarllehtl. 1987;36:3445–8.

266. Rovina N, Nikoloutsou I, Katsani G, Dima E, Fransis K, Roussos C, et al. Effectiveness of pharmacotherapy and behavioral interventions for smoking cessation in actual clinical practice. Ther Adv Respir Dis. 2009;3(6):279-87.

267. Russell MAH, Merriman R, Stapleton J, Taylor W. Effect of Nicotine Chewing Gum as an Adjunct to General-Practitioners Advice against Smoking. British Medical Journal. 1983;287(6407):1782-5.

268. Sachs DPL, Sawe U, Leischow SJ. Effectiveness of a 16-Hour Transdermal Nicotine Patch in a Medical-Practice Setting, without Intensive Group-Counseling. Archives of Internal Medicine. 1993;153(16):1881-90.

269. Sadr Azodi O, Lindstrom D, Adami J, Tonnesen H, Nasell H, Gilljam H, et al. The efficacy of a smoking cessation programme in patients undergoing elective surgery: a randomised clinical trial. Anaesthesia. 2009;64(3):259-65.

270. Schauffler HH, McMenamin S, Olson K, Boyce-Smith G, Rideout JA, Kamil J. Variations in treatment benefits influence smoking cessation: results of a randomised controlled trial. Tob Control. 2001;10(2):175-80.

271. Schiller KR, Luo X, Anderson AJ, Jensen JA, Allen SS, Hatsukami DK. Comparing an immediate cessation versus reduction approach to smokeless tobacco cessation. Nicotine Tob Res. 2012;14(8):902-9.

272. Schmitz JM, Stotts AL, Mooney ME, Delaune KA, Moeller GF. Bupropion and cognitive-behavioral therapy for smoking cessation in women. Nicotine Tob Res. 2007;9(6):699-709.

273. Schneider NG, Jarvik ME, Forsythe AB, Read LL, Elliott ML, Schweiger A. Nicotine gum in smoking cessation: a placebo-controlled, double-blind trial. Addict Behav. 1983;8(3):253-61.

274. Schneider NG, Olmstead R, Mody FV, Doan K, Franzon M, Jarvik ME, et al. Efficacy of a nicotine nasal spray in smoking cessation: a placebo-controlled, double-blind trial. Addiction. 1995;90(12):1671-82.

275. Schneider NG, Olmstead R, Nilsson F, Mody FV, Franzon M, Doan K. Efficacy of a nicotine inhaler in smoking cessation: a double-blind, placebo-controlled trial. Addiction. 1996;91(9):1293-306.

276. Schnoll RA, Martinez E, Tatum KL, Weber DM, Kuzla N, Glass M, et al. A bupropion smoking cessation clinical trial for cancer patients. Cancer Causes Control. 2010;21(6):811-20.

277. Schnoll RA, Patterson F, Wileyto EP, Heitjan DF, Shields AE, Asch DA, et al. Effectiveness of extended-duration transdermal nicotine therapy: a randomized trial. Ann Intern Med. 2010;152(3):144-51.

278. Schnoll RA, Martinez E, Tatum KL, Glass M, Bernath A, Ferris D, et al. Nicotine patch vs. nicotine lozenge for smoking cessation: an effectiveness trial coordinated by the Community Clinical Oncology Program. Drug Alcohol Depend. 2010;107(2-3):237-43.

279. Schnoll RA, Goelz PM, Veluz-Wilkins A, Blazekovic S, Powers L, Leone FT, et al. Long-term nicotine replacement therapy: a randomized clinical trial. JAMA Intern Med. 2015;175(4):504-11.

280. Schnoll R, Leone F, Veluz-Wilkins A, Miele A, Hole A, Jao NC, et al. A randomized controlled trial of 24 weeks of varenicline for tobacco use among cancer patients: Efficacy, safety, and adherence. Psychooncology. 2019;28(3):561-9.

281. Segnan N, Ponti A, Battista RN, Senore C, Rosso S, Shapiro SH, et al. A randomized trial of smoking cessation interventions in general practice in Italy. Cancer Causes Control. 1991;2(4):239-46.

282. Selby P BBSN. Retreatment with ZYban SR: 52 week follow-up of a Canadian Multicentre trial (POS3-63). 2003.

283. Selma Bozkurt Z, Serkan Z, Esra K, Esra Aydin S. Comparison of the effectiveness of varenicline, extended-release bupropion and nicotine replacement therapy on the success and the maintenance of a smoking cessation program. Klinik Psikofarmakoloji Bulteni-Bulletin of Clinical Psychopharmacology. 2013;23(3):224-30.

284. Severson HH, Danaher BG, Ebbert JO, van Meter N, Lichtenstein E, Widdop C, et al. Randomized trial of nicotine lozenges and phone counseling for smokeless tobacco cessation. Nicotine Tob Res. 2015;17(3):309-15.

285. Sharifirad GR, Eslami AA, Charkazi A, Mostafavi F, Shahnazi H. The effect of individual counseling, line follow-up, and free nicotine replacement therapy on smoking cessation in the samples of Iranian smokers: Examination of transtheoretical model. J Res Med Sci. 2012;17(12):1128-36.

286. Sharma SK, Mohan A, Singh AD, Mishra H, Jhanjee S, Pandey RM, et al. Impact of nicotine replacement therapy as an adjunct to anti-tuberculosis treatment and behaviour change counselling in newly diagnosed pulmonary tuberculosis patients: an open-label, randomised controlled trial. Sci Rep. 2018;8(1):8828.

287. Sherman SE, Aldana I, Estrada M, York L. Comparing the tolerability and effectiveness of two treatment regimens in a smoking clinic. Mil Med. 2008;173(6):550-4.

288. Shiffman S, Dresler CM, Hajek P, Gilburt SJ, Targett DA, Strahs KR. Efficacy of a nicotine lozenge for smoking cessation. Arch Intern Med. 2002;162(11):1267-76.

289. Shiffman S, Ferguson SG, Strahs KR. Quitting by gradual smoking reduction using nicotine gum: a randomized controlled trial. Am J Prev Med. 2009;36(2):96-104 e1.

290. Siddiqi K, Khan A, Ahmad M, Dogar O, Kanaan M, Newell JN, et al. Action to stop smoking in suspected tuberculosis (ASSIST) in Pakistan: a cluster randomized, controlled trial. Ann Intern Med. 2013;158(9):667-75.

291. Simon JA, Solkowitz SN, Carmody TP, Browner WS. Smoking cessation after surgery. A randomized trial. Arch Intern Med. 1997;157(12):1371-6.

292. Simon JA, Duncan C, Carmody TP, Hudes ES. Bupropion for smoking cessation: a randomized trial. Arch Intern Med. 2004;164(16):1797-803.

293. Simon JA, Duncan C, Huggins J, Solkowitz S, Carmody TP. Sustained-release bupropion for hospital-based smoking cessation: a randomized trial. Nicotine Tob Res. 2009;11(6):663-9.

294. Smith SS, McCarthy DE, Japuntich SJ, Christiansen B, Piper ME, Jorenby DE, et al. Comparative effectiveness of 5 smoking cessation pharmacotherapies in primary care clinics. Arch Intern Med. 2009;169(22):2148-55.

295. SMK20001:A Multi-Center, Double-Blind, Double-Dummy, Placebo-Controlled, Randomized, Parallel Group, Dose Response Evaluation of a New Chemical Entity (NCE) and ZYBAN (bupropion hydrochloride) Sustained Release (300mg/day) versus Placebo As Aids to Smoking Cessation.

296. Solomon LJ, Scharoun GM, Flynn BS, Secker-Walker RH, Sepinwall D. Free nicotine patches plus proactive telephone peer support to help low-income women stop smoking. Prev Med. 2000;31(1):68-74.

297. Solomon LJ, Marcy TW, Howe KD, Skelly JM, Reinier K, Flynn BS. Does extended proactive telephone support increase smoking cessation among low-income women using nicotine patches? Prev Med. 2005;40(3):306-13.

298. Sonderskov J, Olsen J, Sabroe S, Meillier L, Overvad K. Nicotine patches in smoking cessation: A randomized trial among over-the-counter customers in Denmark. American Journal of Epidemiology. 1997;145(4):309-18.

299. Stapleton JA, Russell MA, Feyerabend C, Wiseman SM, Gustavsson G, Sawe U, et al. Dose effects and predictors of outcome in a randomized trial of transdermal nicotine patches in general practice. Addiction. 1995;90(1):31-42.

300. Stapleton J, West R, Hajek P, Wheeler J, Vangeli E, Abdi Z, et al. Randomized trial of nicotine replacement therapy (NRT), bupropion and NRT plus bupropion for smoking cessation: effectiveness in clinical practice. Addiction. 2013;108(12):2193-201.

301. Stein MD, Caviness CM, Kurth ME, Audet D, Olson J, Anderson BJ. Varenicline for smoking cessation among methadone-maintained smokers: a randomized clinical trial. Drug Alcohol Depend. 2013;133(2):486-93.

302. Steinberg MB, Greenhaus S, Schmelzer AC, Bover MT, Foulds J, Hoover DR, et al. Triple-combination pharmacotherapy for medically ill smokers: a randomized trial. Ann Intern Med. 2009;150(7):447-54.

303. Steinberg MB, Randall J, Greenhaus S, Schmelzer AC, Richardson DL, Carson JL. Tobacco dependence treatment for hospitalized smokers: a randomized, controlled, pilot trial using varenicline. Addict Behav. 2011;36(12):1127-32.

304. Stockings EA, Bowman JA, Baker AL, Terry M, Clancy R, Wye PM, et al. 2014 world congress abstracts, 3-6 december 2014, melbourne, australia. Asia Pac J Clin Oncol. 2014;10 Suppl 9:1-263.

305. Sutherland G, Stapleton JA, Russell MAH, Jarvis MJ, Hajek P, Belcher M, et al. Randomized Controlled Trial of Nasal Nicotine Spray in Smoking Cessation. Lancet. 1992;340(8815):324-9.

306. Sutton S, Hallett R. Randomized trial of brief individual treatment for smoking using nicotine chewing gum in a workplace setting. Am J Public Health. 1987;77(9):1210-1.

307. Sutton S, Hallett R. Smoking intervention in the workplace using videotapes and nicotine chewing gum. Prev Med. 1988;17(1):48-59.

308. Swan GE, McAfee T, Curry SJ, Jack LM, Javitz H, Dacey S, et al. Effectiveness of bupropion sustained release for smoking cessation in a health care setting: a randomized trial. Arch Intern Med. 2003;163(19):2337-44.

309. Swanson NA, Burroughs CC, Long MA, Lee RW. Controlled trial for smoking cessation in a navy shipboard population using nicotine patch, sustained-release buproprion, or both. Military Medicine. 2003;168(10):830-4.

310. Tashkin D, Kanner R, Bailey W, Buist S, Anderson P, Nides M, et al. Smoking cessation in patients with chronic obstructive pulmonary disease: a double-blind, placebo-controlled, randomised trial. Lancet. 2001;357(9268):1571-5.

311. Tashkin DP, Rennard S, Hays JT, Ma W, Lawrence D, Lee TC. Effects of varenicline on smoking cessation in patients with mild to moderate COPD: a randomized controlled trial. Chest. 2011;139(3):591-9.

312. Thomsen T, Tonnesen H, Okholm M, Kroman N, Maibom A, Sauerberg ML, et al. Brief smoking cessation intervention in relation to breast cancer surgery: a randomized controlled trial. Nicotine Tob Res. 2010;12(11):1118-24.

313. Tonnesen P, Fryd V, Hansen M, Helsted J, Gunnersen AB, Forchammer H, et al. Effect of nicotine chewing gum in combination with group counseling on the cessation of smoking. N Engl J Med. 1988;318(1):15-8.

314. Tonnesen P, Fryd V, Hansen M, Helsted J, Gunnersen AB, Forchammer H, et al. Two and four mg nicotine chewing gum and group counselling in smoking cessation: an open, randomized, controlled trial with a 22 month follow-up. Addict Behav. 1988;13(1):17-27.

315. Tonnesen P, Norregaard J, Simonsen K, Sawe U. A double-blind trial of a 16-hour transdermal nicotine patch in smoking cessation. N Engl J Med. 1991;325(5):311-5.

316. Tonnesen P, Norregaard J, Mikkelsen K, Jorgensen S, Nilsson F. A double-blind trial of a nicotine inhaler for smoking cessation. JAMA. 1993;269(10):1268-71.

317. Tonnesen P, Paoletti P, Gustavsson G, Russell MA, Saracci R, Gulsvik A, et al. Higher dosage nicotine patches increase one-year smoking cessation rates: results from the European CEASE trial. Collaborative European Anti-Smoking Evaluation. European Respiratory Society. Eur Respir J. 1999;13(2):238-46.

318. Tonnesen P, Mikkelsen KL. Smoking cessation with four nicotine replacement regimes in a lung clinic. Eur Respir J. 2000;16(4):717-22.

319. Tonnesen P, Tonstad S, Hjalmarson A, Lebargy F, Van Spiegel PI, Hider A, et al. A multicentre, randomized, double-blind, placebo-controlled, 1-year study of bupropion SR for smoking cessation. J Intern Med. 2003;254(2):184-92.

320. Tonnesen P, Mikkelsen K, Bremann L. Nurse-conducted smoking cessation in patients with COPD using nicotine sublingual tablets and behavioral support. Chest. 2006;130(2):334-42.

321. Tonnesen P, Lauri H, Perfekt R, Mann K, Batra A. Efficacy of a nicotine mouth spray in smoking cessation: a randomised, double-blind trial. Eur Respir J. 2012;40(3):548-54.

322. Tonstad S, Farsang C, Klaene G, Lewis K, Manolis A, Perruchoud AP, et al. Bupropion SR for smoking cessation in smokers with cardiovascular disease: a multicentre, randomised study. Eur Heart J. 2003;24(10):946-55.

323. Tonstad S, Tonnesen P, Hajek P, Williams KE, Billing CB, Reeves KR, et al. Effect of maintenance therapy with varenicline on smoking cessation: a randomized controlled trial. JAMA. 2006;296(1):64-71.

324. Tromeur C, Le Mao R, Couturand F. Effect of varenicline on smoking cessation in COPD patients recovering from exacerbation: a randomized trial. Fundamental & Clinical Pharmacology. 2018;32:32-.

325. Tsai ST, Cho HJ, Cheng HS, Kim CH, Hsueh KC, Billing CB, Jr., et al. A randomized, placebo-controlled trial of varenicline, a selective alpha4beta2 nicotinic acetylcholine receptor partial agonist, as a new therapy for smoking cessation in Asian smokers. Clin Ther. 2007;29(6):1027-39.

326. Tsukahara H, Noda K, Saku K. A randomized controlled open comparative trial of varenicline vs nicotine patch in adult smokers: efficacy, safety and withdrawal symptoms (the VN-SEESAW study). Circ J. 2010;74(4):771-8.

327. Tuisku A, Salmela M, Nieminen P, Toljamo T. Varenicline and Nicotine Patch Therapies in Young Adults Motivated to Quit Smoking: A Randomized, Placebo-controlled, Prospective Study. Basic Clin Pharmacol Toxicol. 2016;119(1):78-84.

328. Tulloch HE, Pipe AL, Els C, Clyde MJ, Reid RD. Flexible, dual-form nicotine replacement therapy or varenicline in comparison with nicotine patch for smoking cessation: a randomized controlled trial. BMC Med. 2016;14(80):80.

329. University of Michigan, National Heart LaBI. A Randomized Trial of Internet Access to Nicotine Patches. https://ClinicalTrials.gov/show/NCT00534404; 2011.

330. Urdapilleta-Herrera E, Pina-Rosales MF, Vargas-Rojas MI, Ramirez-Venegas A, Sansores-Martinez R. Bupropion together with cognitive-conductual therapy (CBT) is an alternative for a long-term abstinence of smoking. European Respiratory Journal. 2013;42(no pagination).

331. Uyar M, Filiz A, Bayram N, Elbek O, Herken H, Topcu A, et al. A randomized trial of smoking cessation. Medication versus motivation. Saudi Med J. 2007;28(6):922-6.

332. Vial RJ, Jones TE, Ruffin RE, Gilbert AL. Smoking cessation program using nicotine patches linking hospital to the community. Journal of Pharmacy Practice and Research. 2002;32(1):57-62.

333. Villa RS, Alvarez ABD, Hermida JRF. Effectiveness of a multicomponent program to quit smoking with and without nicotine chewing gum. Psicologia Conductual. 1999;7(1):107-18.

334. Wagena EJ, Knipschild PG, Huibers MJH, Wouters EFM, Schayck CPR. Efficacy of bupropion and nortriptyline for smoking cessation among people who are at risk for or have chronic obstructive pulmonary disease: Results from a randomized, placebo-controlled trial. Nicotine & Tobacco Research. 2005;7(4):683-4.

335. Wallstrom M, Nilsson F, Hirsch JM. A randomized, double-blind, placebo-controlled clinical evaluation of a nicotine sublingual tablet in smoking cessation. Addiction. 2000;95(8):1161-71.

336. Walsh MM, Hilton JF, Masouredis CM, Gee L, Chesney MA, Ernster VL. Smokeless tobacco cessation intervention for college athletes: results after 1 year. Am J Public Health. 1999;89(2):228-34.

337. Wang C, Xiao D, Chan KP, Pothirat C, Garza D, Davies S. Varenicline for smoking cessation: a placebo-controlled, randomized study. Respirology. 2009;14(3):384-92.

338. Ward KD, Asfar T, Al Ali R, Rastam S, Weg MW, Eissenberg T, et al. Randomized trial of the effectiveness of combined behavioral/pharmacological smoking cessation treatment in Syrian primary care clinics. Addiction. 2013;108(2):394-403.

339. Weaver K, Kaplan S, Urbanic J, Case D, Zbikowski S, Dakhil C, et al. Incorporating evidence-based smoking cessation into community oncology practices: Feasibility and preliminary efficacy of an enhanced quitline-based smoking cessation intervention for cancer survivors2015; 24:[32 p.]. Available from: http://onlinelibrary.wiley.com/o/cochrane/clcentral/articles/997/CN-01107997/frame.html.

340. Wennike P, Danielsson T, Landfeldt B, Westin A, Tonnesen P. Smoking reduction promotes smoking cessation: results from a double blind, randomized, placebo-controlled trial of nicotine gum with 2-year follow-up. Addiction. 2003;98(10):1395-402.

341. Westergaard CG, Porsbjerg C, Backer V. The effect of Varenicline on smoking cessation in a group of young asthma patients. Respir Med. 2015;109(11):1416-22.

342. Westman EC, Levin ED, Rose JE. The nicotine patch in smoking cessation. A randomized trial with telephone counseling. Arch Intern Med. 1993;153(16):1917-23.

343. Wewers ME, Neidig JL, Kihm KE. The feasibility of a nurse-managed, peer-led tobacco cessation intervention among HIV-positive smokers. J Assoc Nurses AIDS Care. 2000;11(6):37-44.

344. Wiggers LC, Smets EM, Oort FJ, Peters RJ, Storm-Versloot MN, Vermeulen H, et al. The effect of a minimal intervention strategy in addition to nicotine replacement therapy to support smoking cessation in cardiovascular outpatients: a randomized clinical trial. Eur J Cardiovasc Prev Rehabil. 2006;13(6):931-7.

345. Williams KE, Reeves KR, Billing CB, Jr., Pennington AM, Gong J. A double-blind study evaluating the long-term safety of varenicline for smoking cessation. Curr Med Res Opin. 2007;23(4):793-801.

346. Williams JM, Anthenelli RM, Morris CD, Treadow J, Thompson JR, Yunis C, et al. A randomized, double-blind, placebo-controlled study evaluating the safety and efficacy of varenicline for smoking cessation in patients with schizophrenia or schizoaffective disorder. J Clin Psychiatry. 2012;73(5):654-60.

347. Wilson JS, Fitzsimons D, Bradbury I, Stuart Elborn J. Does additional support by nurses enhance the effect of a brief smoking cessation intervention in people with moderate to severe chronic obstructive pulmonary disease? A randomised controlled trial. Int J Nurs Stud. 2008;45(4):508-17.

348. Winhusen TM, Brigham GS, Kropp F, Lindblad R, Gardin JG, 2nd, Penn P, et al. A randomized trial of concurrent smoking-cessation and substance use disorder treatment in stimulant-dependent smokers. J Clin Psychiatry. 2014;75(4):336-43.

349. Wiratmoko MR, Yunus F, Susanto AD, Ginting TT, Kekalih A. Efficacy of Varenicline, an Nicotinic Acetylcholine Receptor Partial Agonist, Vs Placebo for Smoking Cessation. A Randomized Controlled Trial. Respirology. 2013;18:66-.

350. Wittchen HU, Hoch E, Klotsche J, Muehlig S. Smoking cessation in primary care - a randomized controlled trial of bupropione, nicotine replacements, CBT and a minimal intervention. Int J Methods Psychiatr Res. 2011;20(1):28-39.

351. Wong GY, Wolter TD, Croghan GA, Croghan IT, Offord KP, Hurt RD. A randomized trial of naltrexone for smoking cessation. Addiction. 1999;94(8):1227-37.

352. Wong J, Abrishami A, Yang Y, Zaki A, Friedman Z, Selby P, et al. A perioperative smoking cessation intervention with varenicline: a double-blind, randomized, placebo-controlled trial. Anesthesiology. 2012;117(4):755-64.

353. Wong J, Abrishami A, Riazi S, Siddiqui N, You-Ten E, Korman J, et al. A Perioperative Smoking Cessation Intervention With Varenicline, Counseling, and Fax Referral to a Telephone Quitline Versus a Brief Intervention: A Randomized Controlled Trial. Anesth Analg. 2017;125(2):571-9.

354. Yang DX, Gu CJ, Ni L, Li N, Li QY, Zhou JP. Assessment of efficacy of medication combined with WeChat platform for quitting smoking in patients with chronic obstructive pulmonary disease. [Chinese]. Journal of Shanghai Jiaotong University (Medical Science). 2016;36(3):385-9.

355. Yuhongxia L. The Compliance of Vanrenicline Usage and the Smoking Abstinence Rate Via Mobile Phone Text Messaging Combine with Varenicline: A Single-Blind, Randomised Control Trial. Respirology. 2011;16:46-7.

356. Zellweger JP, Boelcskei PL, Carrozzi L, Sepper R, Sweet R, Hider AZ. Bupropion SR vs placebo for smoking cessation in health care professionals. Am J Health Behav. 2005;29(3):240-9.

357. Zelman DC, Brandon TH, Jorenby DE, Baker TB. Measures of affect and nicotine dependence predict differential response to smoking cessation treatments. J Consult Clin Psychol. 1992;60(6):943-52.

358. Zernig G, Wallner R, Grohs U, Kriechbaum N, Kemmler G, Saria A. A randomized trial of short psychotherapy versus sustained-release bupropion for smoking cessation. Addiction. 2008;103(12):2024-31.

359. ZYB 30011:A multicentre, randomised, double- blind, placebo controlled study to evaluate the efficacy and tolerability of bupropion hydrochloride (SR) sustained release (2 x 150mg per day) versus placebo as an aid to smoking cessation in smokers with at least one cardiovascular (CV) risk factor.

360. ZYB40005: The effect of sustained-release bupropion HCl vs. placebo as an aid to smoking reduction leading to cessation among smokers unwilling and unable to quit smoking.

361. ZYBAKIA402: A single center evaluation of Wellbutrin (bupropion hydrochloride) versus placebo as an aid to smoking cessation (study 402).

# Figure S.1 Risk of bias summary

# Table S.1 Comparison of different NMA models for effectiveness outcomes

|  | Residual deviance | DIC | SD_d_ (95% CrI) | SD_D_ (95% CrI) |
| --- | --- | --- | --- | --- |
| **Sustained abstinence (349 data points)** |  |  |  |  |
| Full interaction model, consistency | 340.0 | 2194 | 0.41 (0.34,0.49) | - |
| Random Class model, consistency | 338.3 | 2187 | 0.40 (0.34,0.48) | 0.12 (0.01,0.32) |
| Fixed Class model, consistency | 338.5 | 2186 | 0.41 (0.34,0.49) | - |
| Fixed Class model, inconsistency | 353.7 | 2209 | 0.32 (0.25,0.40) | - |
| **Prolonged abstinence (32 data points)** |  |  |  |  |
| Full interaction model, consistency | 30.0 | 182.8 | 0.18 (0.01,0.69) | - |
| Random Class model, consistency | 29.5 | 182.6 | 0.18 (0.01,0.7) | 1.33 (0.07,4.58) |
| Fixed Class model, consistency | 32.0 | 182.9 | 0.18 (0.01,0.71) | - |
| Fixed Class model, inconsistency | 32.2 | 186.7 | 0.30 (0.01,1.05) | - |
| **Any abstinence (431 data points)** |  |  |  |  |
| Full interaction model, consistency | 428.6 | 2720 | 0.42 (0.35,0.50) | - |
| Random Class model, consistency | 426.7 | 2711 | 0.14 (0.35,0.48) | 0.14 (0.01,0.31) |
| Fixed Class model, consistency | 426.1 | 2710 | 0.42 (0.36,0.50) | - |
| Fixed Class model, inconsistency | 443.9 | 2723 | 0.34 (0.27,0.41) | - |
| **Seven-day PPA (265 data points)** |  |  |  |  |
| Full interaction model, consistency | 272.3 | 1672 | 0.23 (0.14,0.33) | - |
| Random Class model, consistency | 275.0 | 1663 | 0.22 (0.13,0.31) | 0.12 (0.01,0.34) |
| Fixed Class model, consistency | 274.8 | 1662 | 0.23 (0.15,0.32) | - |
| Fixed Class model, inconsistency | 276.0 | 1671 | 0.21 (0.12,0.31) | - |

DIC: Deviance Information Criterion; SD_d_: standard deviation across treatment effect estimates; SD_D_: standard deviation across class effect estimates.

# Table S.2 Results for sustained abstinence: comparisons with placebo

|  | **Direct evidence, OR (95% CrI)** | **Indirect evidence, OR (95% CrI)** | **NMA, OR (95% CrI)** |
| --- | --- | --- | --- |
| No Drug Treatment | 0.70 (0.08 to 4.01) | 1.30 (1.02 to 1.64) | 1.28 (1.01 to 1.63) |
| Usual Care | 0.81 (0.38 to 1.72) | 0.41 (0.27 to 0.63) | 0.49 (0.33 to 0.71) |
| NRT not specified (ns) | 1.86 (1.55 to 2.25) | - | 1.86 (1.55 to 2.25) |
| NRT Low | - | 0.58 (0.10 to 2.36) | 0.58 (0.10 to 2.36) |
| NRT Std | 2.01 (1.68 to 2.41) | - | 2.01 (1.68 to 2.41) |
| NRT High | 2.32 (1.88 to 2.86) | - | 2.32 (1.88 to 2.86) |
| Bupropion ns | - | 0.17 (0.01 to 1.27) | 0.17 (0.01 to 1.27) |
| Bupropion Low | 0.96 (0.44 to 2.05) | 3.04 (1.46 to 6.33) | 1.75 (1.03 to 3.00) |
| Bupropion Std | 1.73 (1.43 to 2.10) | - | 1.73 (1.43 to 2.10) |
| Varenicline ns | 3.56 (1.22 to 10.7) | - | 3.56 (1.22 to 10.7) |
| Varenicline Low | 1.79 (1.07 to 2.97) | - | 1.79 (1.07 to 2.97) |
| Varenicline Std | 2.83 (2.34 to 3.39) | - | 2.83 (2.34 to 3.39) |
| E-Cigarette Low | 3.22 (0.97 to 12.6) | - | 3.22 (0.97 to 12.6) |
| E-Cigarette High | 2.46 (0.86 to 7.03) | 3.89 (1.63 to 9.28) | 3.22 (1.63 to 6.36) |
| Bupropion ns + NRT ns | - | 1.04 (0.22 to 4.10) | 1.04 (0.22 to 4.10) |
| Bupropion Std + NRT ns | - | 1.62 (0.73 to 3.56) | 1.62 (0.73 to 3.56) |
| Bupropion Std + NRT High | - | 1.99 (0.70 to 5.47) | 1.99 (0.70 to 5.47) |
| Varenicline Low + NRT Std | - | 5.70 (1.57 to 21.1) | 5.70 (1.57 to 21.1) |
| Varenicline Std + NRT Std | - | 5.75 (2.27 to 14.9) | 5.75 (2.27 to 14.9) |
| Varenicline Std + NRT High | - | 2.34 (1.12 to 4.90) | 2.34 (1.12 to 4.90) |
| Varenicline Std + Bupropion Std | 3.42 (1.39 to 8.67) | 1.88 (0.09 to 39.9) | 3.25 (1.35 to 7.92) |

# Table S.3 Results for sustained abstinence: pair-wise comparisons of interventions

|  | **Direct evidence, OR (95% CrI)** | **Indirect evidence, OR (95% CrI)** | **NMA, OR (95% CrI)** |
| --- | --- | --- | --- |
| Bupropion Std vs. NRT Std | 0.70 (0.17 to 2.83) | 0.87 (0.67 to 1.12) | 0.86 (0.67 to 1.11) |
| Varenicline Std vs. NRT Std | - | 1.40 (1.10 to 1.78) | 1.40 (1.10 to 1.78) |
| E-Cigarette Low vs. NRT Std | - | 1.60 (0.48 to 6.30) | 1.60 (0.48 to 6.30) |
| E-Cigarette High vs. NRT Std | - | 1.60 (0.80 to 3.20) | 1.60 (0.80 to 3.20) |
| Varenicline Std + NRT Std vs. NRT Std | - | 2.87 (1.11 to 7.49) | 2.87 (1.11 to 7.49) |
| Varenicline Std + Bupropion Std vs. NRT Std | - | 1.61 (0.66 to 3.98) | 1.61 (0.66 to 3.98) |
| Varenicline Std vs. Bupropion Std | - | 1.63 (1.27 to 2.07) | 1.63 (1.27 to 2.07) |
| E-Cigarette Low vs. Bupropion Std | - | 1.85 (0.55 to 7.29) | 1.85 (0.55 to 7.29) |
| E-Cigarette High vs. Bupropion Std | - | 1.86 (0.92 to 3.73) | 1.86 (0.92 to 3.73) |
| Varenicline Std + NRT Std vs. Bupropion Std | - | 3.34 (1.28 to 8.65) | 3.34 (1.28 to 8.65) |
| Varenicline Std + Bupropion Std vs. Bupropion Std | - | 1.87 (0.76 to 4.59) | 1.87 (0.76 to 4.59) |
| E-Cigarette Low vs. Varenicline Std | - | 1.14 (0.34 to 4.47) | 1.14 (0.34 to 4.47) |
| E-Cigarette High vs. Varenicline Std | - | 1.14 (0.57 to 2.30) | 1.14 (0.57 to 2.30) |
| Varenicline Std + NRT Std vs. Varenicline Std | 2.05 (0.82 to 5.17) | - | 2.05 (0.82 to 5.17) |
| Varenicline Std + Bupropion Std vs. Varenicline Std | - | 1.15 (0.48 to 2.76) | 1.15 (0.48 to 2.76) |
| E-Cigarette High vs. E-Cigarette Low | - | 1.00 (0.22 to 4.03) | 1.00 (0.22 to 4.03) |
| Varenicline Std + NRT Std vs. E-Cigarette Low | - | 1.80 (0.34 to 8.39) | 1.80 (0.34 to 8.39) |
| Varenicline Std + Bupropion Std vs. E-Cigarette Low | - | 1.01 (0.20 to 4.41) | 1.01 (0.20 to 4.41) |
| Varenicline Std + NRT Std vs. E-Cigarette High | - | 1.79 (0.57 to 5.71) | 1.79 (0.57 to 5.71) |
| Varenicline Std + Bupropion Std vs. E-Cigarette High | - | 1.00 (0.33 to 3.08) | 1.00 (0.33 to 3.08) |
| Varenicline Std + Bupropion Std vs. Varenicline Std + NRT Std | - | 0.56 (0.16 to 1.98) | 0.56 (0.16 to 1.98) |

# Results of threshold analysis for sustained abstinence

Threshold analysis, a form of sensitivity analysis, was performed to determine the robustness of our treatment recommendations for the primary effectiveness outcome to changes in the evidence provided by the individual studies.^6,7^ Threshold analysis determines how much the evidence could change - for any reason, such a bias or random error - before the treatment ranking changes and describes this using a set of thresholds. These thresholds can be compared with judgements of the plausible magnitude of potential biases and with estimates of uncertainty (e.g. confidence intervals). In this manner, we may have more confidence in conclusions that are shown to be robust, and can appropriately acknowledge where conclusions are shown to be sensitive to plausible biases or uncertainty in the evidence.

Varenicline Std + NRT Std had the highest estimated odds of sustained abstinence and was the first ranked treatment in our analyses. The *figure* shows the results of the threshold analysis for sustained abstinence, focusing on a selection of the eight treatment classes considered most relevant (and examined in the rankograms presented later). Threshold analysis determines how much the evidence could change before the first ranked treatment changes. Each row in the *figure* corresponds to a single study estimate, and displays the estimate (log odds ratio) and 95% Confidence Interval from that study, along with the invariant interval (shaded bar). Any changes to the study estimate that lie within the invariant interval will not affect the first-ranked treatment. Changes that pass the thresholds at either end of the invariant interval will result in a new first-ranked treatment, which are shown as numeric treatment codes at either side of the invariant interval.

The *figure* also shows the individual study risk of bias judgements. The smallest threshold is 0.44 (on the log odds scale) for the Cinciripini et al. 2018 ^206^ study estimate of Varenicline Std + Bupropion Std vs. Placebo; if the log OR of this estimate changes from 1.63 to 2.07 (= 1.63 + 0.44) or higher in favour of Varenicline Std + Bupropion Std, then Varenicline Std + Bupropion Std would become the first-ranked treatment. This study was rated as low risk of bias; however, the upper end of the 95% confidence interval for the study estimate crosses this threshold, meaning that the first-place ranking is sensitive to the level of uncertainty in this study estimate. The second smallest threshold is -0.47 (on the log odds scale) for the Kogelenberg 2014 study ^350^ estimate of Varenicline Std + NRT Std vs. Varenicline Std; if the log OR of this estimate changes from 0.71 to 0.24 (= 0.71 – 0.47) or lower, in favour of Varenicline Std, then E-Cigarette Low would become the first-ranked treatment. This study was rated as unclear risk of bias, and it may be judged whether any bias due to inadequate random sequence generation or allocation concealment would lead to such an overestimation of treatment effects. There is no threshold in the other direction for this study estimate, indicated by “NT” in the invariant interval; no amount of change to this estimate in favour of Varenicline Std + NRT would change the first-ranked treatment. Only one other study (Caponnetto et al. (2013), E-Cigarette Low vs. Placebo)^201^ has a threshold smaller than 0.7 log OR (equivalent to a factor of 2 on the OR scale); the threshold is 0.61 in the direction favouring E-Cigarette Low, at which point E-Cigarette Low would be the first-ranked treatment. The upper end of the 95% confidence interval for the study estimate crosses this threshold, meaning that the first-place ranking is sensitive to the level of uncertainty in this study estimate. Only another two studies (Hajek et al. (2019),^19^ Bullen et al. (2013)^191^ have thresholds smaller than 3 log OR (equivalent to a factor of 20 on the OR scale). Bullen et al. (2013)^191^ is rated at low risk of bias, but Hajek et al. (2019)^19^ is rated as high or unclear risk of bias for blinding. To change the first-ranked treatment (to E-Cigarette High), the Hajek et al. (2019)^19^ estimate of E-Cigarette High vs. NRT ns would have to underestimate the true odds ratio by a factor of 2.6. The remaining 212 study estimates have even larger thresholds, and it is unlikely that any potential biases could plausibly change these estimates by such an amount to affect the first-place ranking.

Overall, the first-place ranking of Varenicline Std + NRT Std appears relatively robust. However, there is some sensitivity to the level of uncertainty and potential biases in the evidence, which could lead to either Varenicline + Bupropion Std, E-Cigarette Low, or E-Cigarette High being ranked first for sustained abstinence.

6. Phillippo DM, Dias S, Ades AE, Didelez V, Welton NJ. Sensitivity of treatment recommendations to bias in network meta-analysis. J R Stat Soc Ser A Stat Soc. 2018;181(3):843-67.

7. Phillippo DM, Dias S, Welton NJ, Caldwell DM, Taske N, Ades AE. Threshold Analysis as an Alternative to GRADE for Assessing Confidence in Guideline Recommendations Based on Network Meta-analysesThreshold Analysis in Guideline Development. Annals of Internal Medicine. 2019;170(8):538-46.

Figure S.2 Threshold analysis results for sustained abstinence, sorted by size of threshold (smallest to largest).

Only studies with thresholds less than 10 log OR are shown, for brevity. Treatment codes are: (1) Placebo (2) No Drug Treatment (4) Usual Care (5) NRT ns (7) NRT Std (8) NRT High (11) Bupropion Std (14) Varenicline Std (17) E-Cigarette Low (18) E-Cigarette High (26) Varenicline Std + NRT Std (28) Varenicline + Bupropion Std. Bold study labels and red shaded invariant intervals show where a 95% confidence interval crosses the corresponding threshold, indicating sensitivity to the level of uncertainty in this estimate. NT = no threshold. RSG = random sequence generation; AC = allocation concealment; BPP = blinding of participants and personnel; BOA = blinding of outcome assessment; IOD = incomplete outcome data; SR = selective reporting; OB = other bias. “+” = low risk of bias; “?” = unclear risk of bias; “-“ = high risk of bias.

^19, 33, 69, 171, 172, 191, 197, 201, 206, 238, 251, 293, 305, 391, 453, 472, 475, 511^

# Table S.4 Results for prolonged abstinence: comparisons with placebo

|  | **Direct evidence, OR (95% CrI)** | **Indirect evidence, OR (95% CrI)** | **NMA, OR (95% CrI)** |
| --- | --- | --- | --- |
| NRT ns | 1.31 (0.53 to 3.29) | 1.18 (0.38 to 3.67) | 1.26 (0.64 to 2.53) |
| NRT High | 2.18 (0.43 to 11.0) | 2.12 (0.79 to 5.69) | 2.14 (1.01 to 5.42) |
| Bupropion Std | 2.39 (1.31 to 4.39) | 2.26 (1.03 to 4.95) | 2.34 (1.46 to 3.86) |
| Varenicline Std | 3.67 (1.93 to 7.17) | 3.56 (1.42 to 8.94) | 3.63 (2.23 to 6.36) |
| Bupropion Std + NRT High | - | 0.49 (0.02 to 8.08) | 0.49 (0.02 to 8.08) |
| Varenicline Std + Bupropion Std | 5.05 (1.75 to 24.1) | 4.64 (2.00 to 10.8) | 4.76 (2.48 to 10.1) |

# Table S.5 Results for prolonged abstinence: pair-wise comparisons of interventions

|  | **Direct evidence, OR (95% CrI)** | **Indirect evidence, OR (95% CrI)** | **NMA, OR (95% CrI)** |
| --- | --- | --- | --- |
| Varenicline Std vs. Bupropion Std | - | 1.57 (0.86 to 2.92) | 1.57 (0.86 to 2.92) |
| Varenicline Std + Bupropion Std vs. Bupropion Std | - | 2.03 (0.97 to 4.61) | 2.03 (0.97 to 4.61) |
| Varenicline Std + Bupropion Std vs. Varenicline Std | 1.39 (0.49 to 3.94) | 1.28 (0.71 to 2.33) | 1.31 (0.80 to 2.26) |

# Table S.6 Results for any abstinence: comparisons with placebo

|  | **Direct evidence, OR (95% CrI)** | **Indirect evidence, OR (95% CrI)** | **NMA, OR (95% CrI)** |
| --- | --- | --- | --- |
| No Drug Treatment | 1.01 (0.58 to 1.75) | 1.61 (1.24 to 2.09) | 1.48 (1.19 to 1.86) |
| Wait List | - | 1.09 (0.51 to 2.29) | 1.09 (0.51 to 2.29) |
| Usual Care | 0.93 (0.47 to 1.82) | 0.58 (0.37 to 0.90) | 0.66 (0.46 to 0.96) |
| NRT ns | 1.93 (1.60 to 2.36) | 1.57 (1.05 to 2.35) | 1.86 (1.57 to 2.20) |
| NRT Low | 1.51 (0.61 to 3.86) | 1.17 (0.26 to 5.20) | 1.40 (0.63 to 3.06) |
| NRT Std | 2.03 (1.72 to 2.44) | - | 2.03 (1.72 to 2.44) |
| NRT High | 2.39 (1.92 to 2.97) | 2.84 (1.77 to 4.54) | 2.46 (2.03 to 2.94) |
| Bupropion ns | - | 0.19 (0.01 to 1.43) | 0.19 (0.01 to 1.43) |
| Bupropion Low | 5.58 (0.15 to 3041) | 2.84 (1.31 to 6.15) | 2.89 (1.34 to 6.23) |
| Bupropion Std | 1.84 (1.57 to 2.16) | - | 1.84 (1.57 to 2.16) |
| Varenicline ns | 4.06 (1.40 to 11.9) | - | 4.06 (1.40 to 11.9) |
| Varenicline Low | 1.52 (0.84 to 2.75) | - | 1.52 (0.84 to 2.75) |
| Varenicline Std | 2.69 (2.27 to 3.19) | - | 2.69 (2.27 to 3.19) |
| E-Cigarette Low | 2.51 (0.78 to 9.12) | 11.1 (0.81 to 153) | 3.29 (1.13 to 10.8) |
| E-Cigarette High | 2.64 (0.88 to 7.85) | 3.79 (0.24 to 59.1) | 2.77 (1.01 to 7.69) |
| Bupropion ns + NRT ns | - | 1.12 (0.24 to 4.44) | 1.12 (0.24 to 4.44) |
| Bupropion Low + NRT High | - | 5.75 (1.79 to 19.1) | 5.75 (1.79 to 19.1) |
| Bupropion Std + NRT ns | 1.88 (0.87 to 4.06) | 2.36 (1.07 to 5.18) | 2.10 (1.22 to 3.60) |
| Bupropion Std + NRT Std | 1.48 (0.61 to 3.63) | 0.74 (0.01 to 51.7) | 1.43 (0.60 to 3.46) |
| Bupropion Std + NRT High | 2.48 (1.39 to 4.48) | 2.70 (1.23 to 5.91) | 2.56 (1.60 to 4.14) |
| Varenicline Std + NRT Std | - | 5.53 (2.12 to 14.4) | 5.53 (2.12 to 14.4) |
| Varenicline Std + NRT High | - | 2.36 (1.12 to 4.90) | 2.36 (1.12 to 4.90) |
| Varenicline Std + Bupropion Std | 3.32 (1.28 to 8.94) | 3.78 (1.52 to 9.38) | 3.56 (1.84 to6.89) |
| E-Cigarette High + NRT ns | - | 4.76 (0.62 to 47.8) | 4.76 (0.62 to 47.8) |

# Table S.7 Results for any abstinence: pair-wise comparisons of interventions

|  | **Direct evidence, OR (95% CrI)** | **Indirect evidence, OR (95% CrI)** | **NMA, OR (95% CrI)** |
| --- | --- | --- | --- |
| Bupropion Std vs. NRT Std | - | 0.90 (0.72 to 1.13) | 0.90 (0.72 to 1.13) |
| Varenicline Std vs. NRT Std | - | 1.32 (1.05 to 1.65) | 1.32 (1.05 to 1.65) |
| E-Cigarette Low vs. NRT Std | - | 1.60 (0.55 to 5.38) | 1.60 (0.55 to 5.38) |
| E-Cigarette High vs. NRT Std | - | 1.35 (0.49 to 3.76) | 1.35 (0.49 to 3.76) |
| Bupropion Std + NRT Std vs. NRT Std | - | 0.71 (0.29 to 1.72) | 0.71 (0.29 to 1.72) |
| Varenicline Std + NRT Std vs. NRT Std | - | 2.70 (1.02 to 7.13) | 2.70 (1.02 to 7.13) |
| Varenicline Std + Bupropion Std vs. NRT Std | - | 1.75 (0.88 to 3.45) | 1.75 (0.88 to 3.45) |
| Varenicline Std vs. Bupropion Std | - | 1.46 (1.18 to 1.81) | 1.46 (1.18 to 1.81) |
| E-Cigarette Low vs. Bupropion Std | - | 1.78 (0.61 to 5.95) | 1.78 (0.61 to 5.95) |
| E-Cigarette High vs. Bupropion Std | - | 1.50 (0.54 to 4.19) | 1.50 (0.54 to 4.19) |
| Bupropion Std + NRT Std vs. Bupropion Std | - | 0.78 (0.33 to 1.89) | 0.78 (0.33 to 1.89) |
| Varenicline Std + NRT Std vs. Bupropion Std | - | 2.99 (1.13 to 7.88) | 2.99 (1.13 to 7.88) |
| Varenicline Std + Bupropion Std vs. Bupropion Std | - | 1.93 (0.98 to 3.79) | 1.93 (0.98 to 3.79) |
| E-Cigarette Low vs. Varenicline Std | - | 1.22 (0.42 to 4.07) | 1.22 (0.42 to 4.07) |
| E-Cigarette High vs. Varenicline Std | - | 1.03 (0.37 to 2.86) | 1.03 (0.37 to 2.86) |
| Bupropion Std + NRT Std vs. Varenicline Std | - | 0.53 (0.22 to 1.30) | 0.53 (0.22 to 1.30) |
| Varenicline Std + NRT Std vs. Varenicline Std | 2.05 (0.80 to 5.25) | - | 2.05 (0.80 to 5.25) |
| Varenicline Std + Bupropion Std vs. Varenicline Std | 1.50 (0.70 to 3.27) | 0.95 (0.28 to 3.21) | 1.32 (0.69 to 2.52) |
| E-Cigarette High vs. E-Cigarette Low | - | 0.84 (0.18 to 3.69) | 0.84 (0.18 to 3.69) |
| Bupropion Std + NRT Std vs. E-Cigarette Low | - | 0.44 (0.10 to 1.74) | 0.44 (0.10 to 1.74) |
| Varenicline Std + NRT Std vs. E-Cigarette Low | - | 1.67 (0.37 to 6.99) | 1.67 (0.37 to 6.99) |
| Varenicline Std + Bupropion Std vs. E-Cigarette Low | - | 1.08 (0.28 to 3.82) | 1.08 (0.28 to 3.82) |
| Bupropion Std + NRT Std vs. E-Cigarette High | - | 0.52 (0.14 to 1.98) | 0.52 (0.14 to 1.98) |
| Varenicline Std + NRT Std vs. E-Cigarette High | - | 2.00 (0.49 to 8.02) | 2.00 (0.49 to 8.02) |
| Varenicline Std + Bupropion Std vs. E-Cigarette High | - | 1.29 (0.38 to 4.27) | 1.29 (0.38 to 4.27) |
| Varenicline Std + NRT Std vs. Bupropion Std + NRT Std | - | 3.83 (1.05 to 14.0) | 3.83 (1.05 to 14.0) |
| Varenicline Std + Bupropion Std vs. Bupropion Std + NRT Std | - | 2.48 (0.83 to 7.34) | 2.48 (0.83 to 7.34) |
| Varenicline Std + Bupropion Std vs. Varenicline Std + NRT Std | - | 0.65 (0.21 to 2.04) | 0.65 (0.21 to 2.04) |

# Table S.8 Results for PPA: comparisons with placebo

|  | **Direct evidence, OR (95% CrI)** | **Indirect evidence, OR (95% CrI)** | **NMA, OR (95% CrI)** |
| --- | --- | --- | --- |
| No Drug Treatment | 1.02 (0.64 to 1.62) | 1.18 (0.87 to 1.61) | 1.13 (0.87 to 1.45) |
| Wait List | - | 0.98 (0.53 to 1.79) | 0.98 (0.53 to 1.79) |
| Usual Care | 0.84 (0.15 to 3.32) | 1.10 (0.67 to 1.80) | 1.07 (0.66 to 1.72) |
| NRT ns | 1.60 (1.22 to 2.12) | 1.87 (1.48 to 2.35) | 1.75 (1.48 to 2.08) |
| NRT Low | - | 1.48 (0.70 to 3.13) | 1.48 (0.70 to 3.13) |
| NRT Std | 1.58 (1.21 to 2.12) | - | 1.58 (1.21 to 2.12) |
| NRT High | 2.20 (1.73 to 2.80) | 1.75 (1.34 to 2.29) | 1.99 (1.67 to 2.39) |
| Bupropion Low | 1.21 (0.78 to 1.90) | - | 1.21 (0.78 to 1.90) |
| Bupropion Std | 1.67 (1.48 to 1.88) | - | 1.67 (1.48 to 1.88) |
| Varenicline ns | 2.56 (1.21 to 5.42) | - | 2.56 (1.21 to 5.42) |
| Varenicline Low | 1.70 (0.90 to 3.19) | 2.18 (0.40 to 11.8) | 1.75 (0.97 to 3.13) |
| Varenicline Std | 2.14 (1.86 to 2.46) | - | 2.14 (1.86 to 2.46) |
| Bupropion Low + NRT High | - | 4.76 (1.82 to 12.7) | 4.76 (1.82 to 12.7) |
| Bupropion Std + NRT ns | 1.77 (1.01 to 3.13) | 1.87 (1.29 to 2.73) | 1.84 (1.35 to 2.53) |
| Bupropion Std + NRT High | 2.46 (1.57 to 3.90) | 1.91 (1.24 to 2.96) | 2.16 (1.57 to 2.97) |
| Varenicline Std + NRT Std | - | 4.01 (2.16 to 7.54) | 4.01 (2.16 to 7.54) |
| Varenicline Std + NRT High | - | 2.14 (0.91 to 4.85) | 2.14 (0.91 to 4.85) |
| Varenicline & Bupropion Std | 1.75 (0.90 to 3.53) | 2.79 (1.58 to 4.90) | 2.29 (1.48 to 3.56) |
| E-Cigarette High + NRT ns | - | 4.10 (0.63 to 37.7) | 4.10 (0.63 to 37.7) |

# Table S.9 Results for PPA: pair-wise comparisons of interventions

|  | **Direct evidence, OR (95% CrI)** | **Indirect evidence, OR (95% CrI)** | **NMA, OR (95% CrI)** |
| --- | --- | --- | --- |
| Bupropion Std vs. NRT Std | - | 1.05 (0.78 to 1.41) | 1.05 (0.78 to 1.41) |
| Varenicline Std vs. NRT Std | - | 1.35 (0.99 to 1.82) | 1.35 (0.99 to 1.82) |
| Varenicline Std + NRT Std vs. NRT Std | - | 2.54 (1.28 to 4.98) | 2.54 (1.28 to 4.98) |
| Varenicline & Bupropion Std vs. NRT Std | - | 1.44 (0.86 to 2.42) | 1.44 (0.86 to 2.42) |
| Varenicline Std vs. Bupropion Std | - | 1.28 (1.08 to 1.53) | 1.28 (1.08 to 1.53) |
| Varenicline Std + NRT Std vs. Bupropion Std | - | 2.42 (1.28 to 4.57) | 2.42 (1.28 to 4.57) |
| Varenicline & Bupropion Std vs. Bupropion Std | - | 1.38 (0.88 to 2.17) | 1.38 (0.88 to 2.17) |
| Varenicline Std + NRT Std vs. Varenicline Std | 1.88 (1.02 to 3.46) | - | 1.88 (1.02 to 3.46) |
| Varenicline & Bupropion Std vs. Varenicline Std | 1.40 (0.79 to 2.49) | 0.81 (0.44 to 1.46) | 1.07 (0.70 to 1.63) |
| Varenicline & Bupropion Std vs. Varenicline Std + NRT Std | - | 0.57 (0.27 to 1.20) | 0.57 (0.27 to 1.20) |


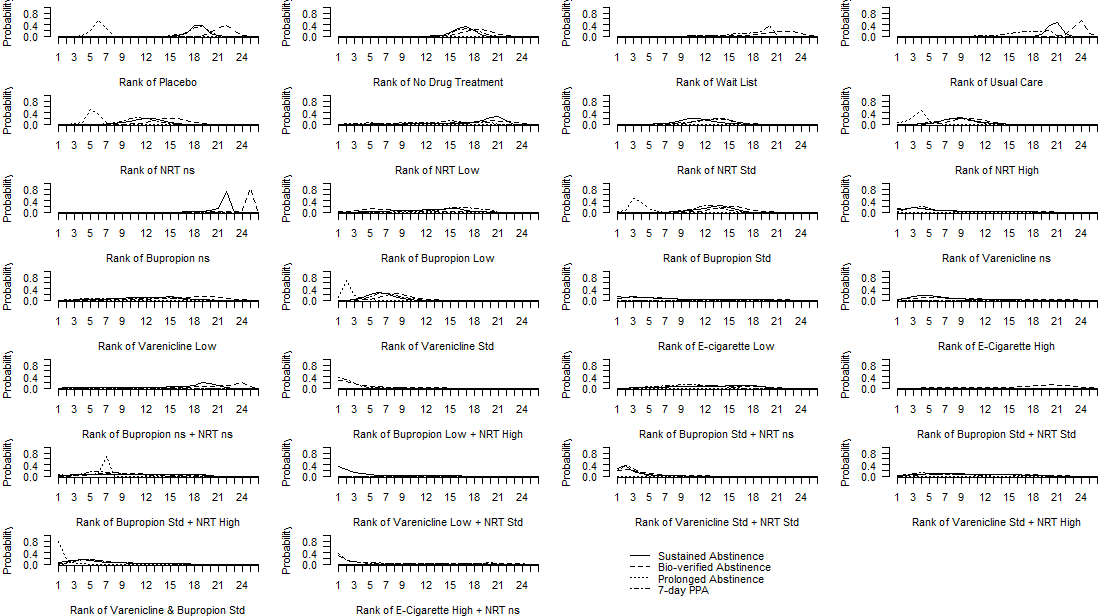


# Figure S.3 Rank-o-gram of intervention classes at all doses across effectiveness outcomes.

# Table S.10 Absolute probability of 1-year continuous cessation based on NRT Std taken from Taylor et al (2017), and odds ratios estimated from the NMA

| **1-year continuous abstinence probability** | **Mean** | **2.5%** | **97.5%** |
| --- | --- | --- | --- |
| Varenicline Low + NRT Std | 0.43 | 0.17 | 0.73 |
| Varenicline Std + NRT Std | 0.43 | 0.23 | 0.66 |
| E-Cigarette Low | 0.32 | 0.12 | 0.63 |
| Varenicline & Bupropion Std | 0.31 | 0.15 | 0.51 |
| E-Cigarette High | 0.30 | 0.18 | 0.45 |
| Varenicline Std | 0.27 | 0.23 | 0.32 |
| Varenicline Std + NRT High | 0.24 | 0.12 | 0.38 |
| NRT High | 0.23 | 0.19 | 0.27 |
| NRT Std | 0.21 | 0.21 | 0.21 |
| Varenicline Low | 0.19 | 0.12 | 0.28 |
| Bupropion Std + NRT High | 0.19 | 0.10 | 0.32 |
| Bupropion Low | 0.19 | 0.12 | 0.28 |
| Bupropion Std | 0.18 | 0.15 | 0.22 |
| NRT Low | 0.07 | 0.02 | 0.16 |

SAFETY

# Reference list of included studies

1. Abelin T, Buehler A, Muller P, Vesanen K, Imhof PR. Controlled trial of transdermal nicotine patch in tobacco withdrawal. Lancet. 1989;1(8628):7-10.

2. Ahluwalia JS, McNagny SE, Clark WS. Smoking cessation among inner-city African Americans using the nicotine transdermal patch. J Gen Intern Med. 1998;13(1):1-8.

3. Ahluwalia JS, Harris KJ, Catley D, Okuyemi KS, Mayo MS. Sustained-release bupropion for smoking cessation in African Americans: a randomized controlled trial. JAMA. 2002;288(4):468-74.

4. AKIA401: A single-center evaluation of Wellbutrin (bupropion hydrochloride) versus placebo as an aid to smoking cessation in heavy smokers (study 401).

5. Allen MH, Debanne M, Lazignac C, Adam E, Dickinson LM, Damsa C. Effect of nicotine replacement therapy on agitation in smokers with schizophrenia: a double-blind, randomized, placebo-controlled study. Am J Psychiatry. 2011;168(4):395-9.

6. Andrews JO, Mueller M, Dooley M, Newman SD, Magwood GS, Tingen MS. Effect of a smoking cessation intervention for women in subsidized neighborhoods: A randomized controlled trial. Prev Med. 2016;90:170-6.

7. Anthenelli RM, Morris C, Ramey TS, Dubrava SJ, Tsilkos K, Russ C, et al. Effects of varenicline on smoking cessation in adults with stably treated current or past major depression: a randomized trial. Ann Intern Med. 2013;159(6):390-400.

8. Anthenelli RM, Benowitz NL, West R, St Aubin L, McRae T, Lawrence D, et al. Neuropsychiatric safety and efficacy of varenicline, bupropion, and nicotine patch in smokers with and without psychiatric disorders (EAGLES): a double-blind, randomised, placebo-controlled clinical trial. Lancet. 2016;387(10037):2507-20.

9. Anthonisen NR, Connett JE, Kiley JP, Altose MD, Bailey WC, Buist AS, et al. Effects of smoking intervention and the use of an inhaled anticholinergic bronchodilator on the rate of decline of FEV1. The Lung Health Study. Jama. 1994;272(19):1497-505.

10. Areechon W, Punnotok J. Smoking Cessation through the Use of Nicotine Chewing Gum - a Double-Blind Trial in Thailand. Clinical Therapeutics. 1988;10(2):183-6.

11. Aubin HJ, Lebargy F, Berlin I, Bidaut-Mazel C, Chemali-Hudry J, Lagrue G. Efficacy of bupropion and predictors of successful outcome in a sample of French smokers: a randomized placebo-controlled trial. Addiction. 2004;99(9):1206-18.

12. Aubin HJ, Bobak A, Britton JR, Oncken C, Billing CB, Jr., Gong J, et al. Varenicline versus transdermal nicotine patch for smoking cessation: results from a randomised open-label trial. Thorax. 2008;63(8):717-24.

13. Austin AJ, Duka T, Rusted J, Jackson A. Effect of varenicline on aspects of inhibitory control in smokers. Psychopharmacology (Berl). 2014;231(18):3771-85.

14. Baker A, Richmond R, Haile M, Lewin TJ, Carr VJ, Taylor RL, et al. A randomized controlled trial of a smoking cessation intervention among people with a psychotic disorder. American Journal of Psychiatry. 2006;163(11):1934-42.

15. Baker TB, Piper ME, Stein JH, Smith SS, Bolt DM, Fraser DL, et al. Effects of Nicotine Patch vs Varenicline vs Combination Nicotine Replacement Therapy on Smoking Cessation at 26 Weeks: A Randomized Clinical Trial. JAMA. 2016;315(4):371-9.

16. Baldassarri SR, Bernstein SL, Chupp GL, Slade MD, Fucito LM, Toll BA. Electronic cigarettes for adults with tobacco dependence enrolled in a tobacco treatment program: A pilot study. Addict Behav. 2018;80:1-5.

17. Batra A, Klingler K, Landfeldt B, Friederich HM, Westin A, Danielsson T. Smoking reduction treatment with 4-mg nicotine gum: a double-blind, randomized, placebo-controlled study. Clin Pharmacol Ther. 2005;78(6):689-96.

18. Benowitz NL, Pipe A, West R, Hays JT, Tonstad S, McRae T, et al. Cardiovascular Safety of Varenicline, Bupropion, and Nicotine Patch in Smokers: A Randomized Clinical Trial. JAMA Intern Med. 2018;178(5):622-31.

19. Bernstein SL, Bijur P, Cooperman N, Jearld S, Arnsten JH, Moadel A, et al. A randomized trial of a multicomponent cessation strategy for emergency department smokers. Acad Emerg Med. 2011;18(6):575-83.

20. Bernstein SL, D'Onofrio G, Rosner J, O'Malley S, Makuch R, Busch S, et al. Successful Tobacco Dependence Treatment in Low-Income Emergency Department Patients: A Randomized Trial. Ann Emerg Med. 2015;66(2):140-7.

21. Bernstein SL, Rosner J, Toll B. A Multicomponent Intervention Including Texting to Promote Tobacco Abstinence in Emergency Department Smokers: A Pilot Study. Acad Emerg Med. 2016;23(7):803-8.

22. Binnie VI, McHugh S, Jenkins W, Borland W, Macpherson LM. A randomised controlled trial of a smoking cessation intervention delivered by dental hygienists: a feasibility study. BMC Oral Health. 2007;7:5.

23. Bloch B, Reshef A, Cohen T, Tafla A, Gathas S, Israel S, et al. Preliminary effects of bupropion and the promoter region (HTTLPR) serotonin transporter (SLC6A4) polymorphism on smoking behavior in schizophrenia. Psychiatry Res. 2010;175(1-2):38-42.

24. Blondal T, Gudmundsson LJ, Olafsdottir I, Gustavsson G, Westin A. Nicotine nasal spray with nicotine patch for smoking cessation: randomised trial with six year follow up. BMJ. 1999;318(7179):285-8.

25. Bohadana A, Nilsson F, Rasmussen T, Martinet Y. Nicotine inhaler and nicotine patch as a combination therapy for smoking cessation: a randomized, double-blind, placebo-controlled trial. Arch Intern Med. 2000;160(20):3128-34.

26. Bolliger CT, Zellweger JP, Danielsson T, van Biljon X, Robidou A, Westin A, et al. Smoking reduction with oral nicotine inhalers: double blind, randomised clinical trial of efficacy and safety. BMJ. 2000;321(7257):329-33.

27. Bolliger CT, Issa JS, Posadas-Valay R, Safwat T, Abreu P, Correia EA, et al. Effects of varenicline in adult smokers: a multinational, 24-week, randomized, double-blind, placebo-controlled study. Clin Ther. 2011;33(4):465-77.

28. Boyle R, Severson H, Lichtenstein E, et al. Smokeless tobacco cessation with nicotine reduction: A placebo controlled trial [abstract]. Paper presented at: 121st Annual Meeting Location: San Francisco, CA. 1992.

29. Brandon TH, Drobes DJ, Unrod M, Heckman BW, Oliver JA, Roetzheim RC, et al. Varenicline effects on craving, cue reactivity, and smoking reward. Psychopharmacology (Berl). 2011;218(2):391-403.

30. Brantmark B, Ohlin P, Westling H. Nicotine-containing chewing gum as an anti-smoking aid. Psychopharmacologia. 1973;31(3):191-200.

31. Buchkremer G, Bents H, Horstmann M, Opitz K, Tölle R. Combination of behavioral smoking cessation with transdermal nicotine substitution. Addictive Behaviors. 1989;14(2):229-38.

32. Bullen C, Howe C, Lin RB, Grigg M, Laugesen M, McRobbie H, et al. Pre-cessation nicotine replacement therapy: pragmatic randomized trial. Addiction. 2010;105(8):1474-83.

33. Bullen C, Howe C, Laugesen M, McRobbie H, Parag V, Williman J, et al. Electronic cigarettes for smoking cessation: A randomised controlled trial. The Lancet. 2013;382(9905):1629-37.

34. Burstein AH, Fullerton T, Clark DJ, Faessel HM. Pharmacokinetics, safety, and tolerability after single and multiple oral doses of varenicline in elderly smokers. J Clin Pharmacol. 2006;46(11):1234-40.

35. Caldwell BO, Adamson SJ, Crane J. Combination rapid-acting nicotine mouth spray and nicotine patch therapy in smoking cessation. Nicotine Tob Res. 2014;16(10):1356-64.

36. Caldwell BO, Crane J. Combination Nicotine Metered Dose Inhaler and Nicotine Patch for Smoking Cessation: A Randomized Controlled Trial. Nicotine Tob Res. 2016;18(10):1944-51.

37. Campbell IA. Comparison of 4 Methods of Smoking Withdrawal in Patients with Smoking Related Diseases. British Medical Journal. 1983;286(6365):595-7.

38. Campbell IA, Prescott RJ, Tjeder-Burton SM. Smoking cessation in hospital patients given repeated advice plus nicotine or placebo chewing gum. Respir Med. 1991;85(2):155-7.

39. Campbell IA, Prescott RJ, Tjeder-Burton SM. Transdermal nicotine plus support in patients attending hospital with smoking-related diseases: a placebo-controlled study. Respir Med. 1996;90(1):47-51.

40. Caponnetto P, Campagna D, Cibella F, Morjaria JB, Caruso M, Russo C, et al. EffiCiency and Safety of an eLectronic cigAreTte (ECLAT) as tobacco cigarettes substitute: a prospective 12-month randomized control design study. PLoS One. 2013;8(6):e66317.

41. Carpenter MJ, Hughes JR, Gray KM, Wahlquist AE, Saladin ME, Alberg AJ. Nicotine therapy sampling to induce quit attempts among smokers unmotivated to quit: a randomized clinical trial. Arch Intern Med. 2011;171(21):1901-7.

42. Carpenter MJ, Heckman BW, Wahlquist AE, Wagener TL, Goniewicz ML, Gray KM, et al. A Naturalistic, Randomized Pilot Trial of E-Cigarettes: Uptake, Exposure, and Behavioral Effects. Cancer Epidemiol Biomarkers Prev. 2017;26(12):1795-803.

43. Carson KV, Smith BJ, Brinn MP, Peters MJ, Fitridge R, Koblar SA, et al. Safety of varenicline tartrate and counseling versus counseling alone for smoking cessation: a randomized controlled trial for inpatients (STOP study). Nicotine Tob Res. 2014;16(11):1495-502.

44. Chen HK, Lan TH, Wu BJ. A double-blind randomized clinical trial of different doses of transdermal nicotine patch for smoking reduction and cessation in long-term hospitalized schizophrenic patients. Eur Arch Psychiatry Clin Neurosci. 2013;263(1):75-82.

45. Chengappa KN, Perkins KA, Brar JS, Schlicht PJ, Turkin SR, Hetrick ML, et al. Varenicline for smoking cessation in bipolar disorder: a randomized, double-blind, placebo-controlled study. J Clin Psychiatry. 2014;75(7):765-72.

46. Christen AG, McDonald JL, Jr., Olson BL, Drook CA, Stookey GK. Efficacy of nicotine chewing gum in facilitating smoking cessation. J Am Dent Assoc. 1984;108(4):594-7.

47. Cinciripini PM, Cinciripini LG, Wallfisch A, Haque W, Van Vunakis H. Behavior therapy and the transdermal nicotine patch: effects on cessation outcome, affect, and coping. J Consult Clin Psychol. 1996;64(2):314-23.

48. Cinciripini PM, Robinson JD, Karam-Hage M, Minnix JA, Lam C, Versace F, et al. Effects of varenicline and bupropion sustained-release use plus intensive smoking cessation counseling on prolonged abstinence from smoking and on depression, negative affect, and other symptoms of nicotine withdrawal. JAMA Psychiatry. 2013;70(5):522-33.

49. Cinciripini PM, Minnix JA, Green CE, Robinson JD, Engelmann JM, Versace F, et al. An RCT with the combination of varenicline and bupropion for smoking cessation: clinical implications for front line use. Addiction. 2018.

50. Cooney NL, Cooney JL, Perry BL, Carbone M, Cohen EH, Steinberg HR, et al. Smoking cessation during alcohol treatment: a randomized trial of combination nicotine patch plus nicotine gum. Addiction. 2009;104(9):1588-96.

51. Cosci F, Ibrahim HM, Nannini A, Schruers K. Experimental study on the effects of anxiety sensitivity and somatosensory amplification on the response to the 35% CO(2) challenge in abstinent smokers. Exp Clin Psychopharmacol. 2015;23(6):464-76.

52. Covey LS, Glassman AH, Jiang H, Fried J, Masmela J, LoDuca C, et al. A randomized trial of bupropion and/or nicotine gum as maintenance treatment for preventing smoking relapse. Addiction. 2007;102(8):1292-302.

53. Cox LS, Nollen NL, Mayo MS, Choi WS, Faseru B, Benowitz NL, et al. Bupropion for smoking cessation in African American light smokers: a randomized controlled trial. J Natl Cancer Inst. 2012;104(4):290-8.

54. Cravo AS, Bush J, Sharma G, Savioz R, Martin C, Craige S, et al. A randomised, parallel group study to evaluate the safety profile of an electronic vapour product over 12 weeks. Regul Toxicol Pharmacol. 2016;81 Suppl 1:S1-S14.

55. Croghan GA, Sloan JA, Croghan IT, Novotny P, Hurt RD, DeKrey WL, et al. Comparison of nicotine patch alone versus nicotine nasal spray alone versus a combination for treating smokers: a minimal intervention, randomized multicenter trial in a nonspecialized setting. Nicotine Tob Res. 2003;5(2):181-7.

56. Cummings KM, Hyland A, Carlin-Menter S, Mahoney MC, Willett J, Juster HR. Costs of giving out free nicotine patches through a telephone quit line. J Public Health Manag Pract. 2011;17(3):E16-23.

57. Cummins SE, Gamst AC, Brandstein K, Seymann GB, Klonoff-Cohen H, Kirby CA, et al. Helping Hospitalized Smokers: A Factorial RCT of Nicotine Patches and Counseling. Am J Prev Med. 2016;51(4):578-86.

58. Dale LC, Hurt RD, Offord KP, Lawson GM, Croghan IT, Schroeder DR. High-dose nicotine patch therapy. Percentage of replacement and smoking cessation. JAMA. 1995;274(17):1353-8.

59. Dale LC, Ebbert JO, Schroeder DR, Croghan IT, Rasmussen DF, Trautman JA, et al. Bupropion for the treatment of nicotine dependence in spit tobacco users: a pilot study. Nicotine Tob Res. 2002;4(3):267-74.

60. Dale LC, Ebbert JO, Glover ED, Croghan IT, Schroeder DR, Severson HH, et al. Bupropion SR for the treatment of smokeless tobacco use. Drug Alcohol Depend. 2007;90(1):56-63.

61. Dalsgareth OJ, Hansen NC, Soes-Petersen U, Evald T, Hoegholm A, Barber J, et al. A multicenter, randomized, double-blind, placebo-controlled, 6-month trial of bupropion hydrochloride sustained-release tablets as an aid to smoking cessation in hospital employees. Nicotine Tob Res. 2004;6(1):55-61.

62. Daughton DM, Heatley SA, Prendergast JJ, Causey D, Knowles M, Rolf CN, et al. Effect of Transdermal Nicotine Delivery as an Adjunct to Low-Intervention Smoking Cessation Therapy - a Randomized, Placebo-Controlled, Double-Blind-Study. Archives of Internal Medicine. 1991;151(4):749-52.

63. Daughton DM, Fortmann SP, Glover ED, Hatsukami DK, Heatley SA, Lichtenstein E, et al. The smoking cessation efficacy of varying doses of nicotine patch delivery systems 4 to 5 years post-quit day. Prev Med. 1999;28(2):113-8.

64. Dautzenberg B, Ruff F, Vaucher M, Maillon P, Jacob N, Kienzler JL, et al. First demonstration of the good efficacy/safety ratio of Nicotinell® 1mg Lozenge (NL 1mg), a new form of nicotine substitution, by randomised clinical trial. European Respiratory Journal. 2001;18 Suppl 33:12s.

65. Davidson M, Epstein M, Burt R, Schaefer C, Whitworth G, McDonald A. Efficacy and safety of an over-the-counter transdermal nicotine patch as an aid for smoking cessation. Archives of Family Medicine. 1998;7(6):569-74.

66. de Jong B, Schuppers AS, Kruisdijk-Gerritsen A, Arbouw MEL, van den Oever HLA, van Zanten ARH. The safety and efficacy of nicotine replacement therapy in the intensive care unit: a randomised controlled pilot study. Ann Intensive Care. 2018;8(1):70.

67. Dogar O, Zahid R, Mansoor S, Kanaan M, Ahluwalia JS, Jawad M, et al. Varenicline versus placebo for waterpipe smoking cessation: a double-blind randomized controlled trial. Addiction. 2018;113(12):2290-9.

68. Du D, Borders J, Selmani A, Waverczak W. A Pilot Study to Investigate the Efficacy of Nicotine Oral Soluble Film, Lozenge and Gum in Relief of Acute Smoking Cue-provoked Craving for Cigarette in Low Dependence Smokers. Journal of Smoking Cessation. 2015;10(2):87-95.

69. Ebbert JO, Dale LC, Patten CA, Croghan IT, Schroeder DR, Moyer TP, et al. Effect of high-dose nicotine patch therapy on tobacco withdrawal symptoms among smokeless tobacco users. Nicotine Tob Res. 2007;9(1):43-52.

70. Ebbert JO, Severson HH, Croghan IT, Danaher BG, Schroeder DR. A randomized clinical trial of nicotine lozenge for smokeless tobacco use. Nicotine Tob Res. 2009;11(12):1415-23.

71. Ebbert JO, Severson HH, Croghan IT, Danaher BG, Schroeder DR. A pilot study of mailed nicotine lozenges with assisted self-help for the treatment of smokeless tobacco users. Addict Behav. 2010;35(5):522-5.

72. Ebbert JO, Croghan IT, Severson HH, Schroeder DR, Hays JT. A pilot study of the efficacy of varenicline for the treatment of smokeless tobacco users in Midwestern United States. Nicotine Tob Res. 2011;13(9):820-6.

73. Ebbert JO, Croghan IT, Schroeder DR, Hurt RD. A randomized phase II clinical trial of high-dose nicotine patch therapy for smokeless tobacco users. Nicotine Tob Res. 2013;15(12):2037-44.

74. Ebbert JO, Hatsukami DK, Croghan IT, Schroeder DR, Allen SS, Hays JT, et al. Combination varenicline and bupropion SR for tobacco-dependence treatment in cigarette smokers: a randomized trial. JAMA. 2014;311(2):155-63.

75. Ebbert JO, Hughes JR, West RJ, Rennard SI, Russ C, McRae TD, et al. Effect of varenicline on smoking cessation through smoking reduction: a randomized clinical trial. JAMA. 2015;313(7):687-94.

76. Ebbert J, Croghan I, Hurt R, Schroeder D, Hays J. Varenicline for smoking cessation in light smokers2017; 18(10):[2031-5 pp.]. Available from: http://onlinelibrary.wiley.com/o/cochrane/clcentral/articles/505/CN-01245505/frame.html.

77. Ehrsam RE, Buhler A, Muller P, Mauli D, Schumacher PM, Howald H, et al. [Weaning of young smokers using a transdermal nicotine patch]. Schweiz Rundsch Med Prax. 1991;80(7):145-50.

78. Eisenberg MJ, Grandi SM, Gervais A, O'Loughlin J, Paradis G, Rinfret S, et al. Bupropion for smoking cessation in patients hospitalized with acute myocardial infarction: a randomized, placebo-controlled trial. J Am Coll Cardiol. 2013;61(5):524-32.

79. Eisenberg MJ, Windle SB, Roy N, Old W, Grondin FR, Bata I, et al. Varenicline for Smoking Cessation in Hospitalized Patients With Acute Coronary Syndrome. Circulation. 2016;133(1):21-30.

80. Ellerbeck EF, Nollen N, Hutcheson TD, Phadnis M, Fitzgerald SA, Vacek J, et al. Effect of Long-term Nicotine Replacement Therapy vs Standard Smoking Cessation for Smokers With Chronic Lung Disease: A Randomized Clinical Trial. JAMA Netw Open. 2018;1(5):e181843.

81. Etter JF, Laszlo E, Zellweger JP, Perrot C, Perneger TV. Nicotine replacement to reduce cigarette consumption in smokers who are unwilling to quit: a randomized trial. J Clin Psychopharmacol. 2002;22(5):487-95.

82. Etter JF, Huguelet P, Perneger TV, Cornuz J. Nicotine Gum Treatment Before Smoking Cessation A Randomized Trial. Archives of Internal Medicine. 2009;169(11):1028-34.

83. Evins AE, Mays VK, Rigotti NA, Tisdale T, Cather C, Goff DC. A pilot trial of bupropion added to cognitive behavioral therapy for smoking cessation in schizophrenia. Nicotine Tob Res. 2001;3(4):397-403.

84. Evins AE, Cather C, Deckersbach T, Freudenreich O, Culhane MA, Olm-Shipman CM, et al. A double-blind placebo-controlled trial of bupropion sustained-release for smoking cessation in schizophrenia. J Clin Psychopharmacol. 2005;25(3):218-25.

85. Evins AE, Cather C, Culhane MA, Birnbaum A, Horowitz J, Hsieh E, et al. A 12-week double-blind, placebo-controlled study of bupropion sr added to high-dose dual nicotine replacement therapy for smoking cessation or reduction in schizophrenia. J Clin Psychopharmacol. 2007;27(4):380-6.

86. Evins AE, Cather C, Pratt SA, Pachas GN, Hoeppner SS, Goff DC, et al. Maintenance treatment with varenicline for smoking cessation in patients with schizophrenia and bipolar disorder: a randomized clinical trial. JAMA. 2014;311(2):145-54.

87. Fagerstrom K, Gilljam H, Metcalfe M, Tonstad S, Messig M. Stopping smokeless tobacco with varenicline: randomised double blind placebo controlled trial. BMJ. 2010;341:c6549.

88. Fatemi SH, Yousefi MK, Kneeland RE, Liesch SB, Folsom TD, Thuras PD. Antismoking and potential antipsychotic effects of varenicline in subjects with schizophrenia or schizoaffective disorder: a double-blind placebo and bupropion-controlled study. Schizophr Res. 2013;146(1-3):376-8.

89. Fee WM, Stewart MJ. A controlled trial of nicotine chewing gum in a smoking withdrawal clinic. Practitioner. 1982;226(1363):148-51.

90. Ferry LH, Robbins AS, Scariati PD, Masterson A, Abbey DE, Burchette RJ. Enhancement of smoking cessation using the antidepressant bupropion hydrochloride [abstract 2670]. Circulation. 1992;86(4 Suppl 1):I–671.

91. Ferry LH, Burchette RJ. Efficacy of Bupropion for Smoking Cessation in Non Depressed Smokers. Journal of Addictive Diseases. 1994;13(4):249-.

92. Fiore MC, Kenford SL, Jorenby DE, Wetter DW, Smith SS, Baker TB. Two studies of the clinical effectiveness of the nicotine patch with different counseling treatments. Chest. 1994;105(2):524-33.

93. Fortmann SP, Killen JD. Nicotine Gum and Self-Help Behavioral Treatment for Smoking Relapse Prevention - Results from a Trial Using Population-Based Recruitment. Journal of Consulting and Clinical Psychology. 1995;63(3):460-8.

94. Fossati R, Apolone G, Negri E, Compagnoni A, La Vecchia C, Mangano S, et al. A double-blind, placebo-controlled, randomized trial of bupropion for smoking cessation in primary care. Arch Intern Med. 2007;167(16):1791-7.

95. Foulds J, Stapleton J, Hayward M, Russell MA, Feyerabend C, Fleming T, et al. Transdermal nicotine patches with low-intensity support to aid smoking cessation in outpatients in a general hospital. A placebo-controlled trial. Arch Fam Med. 1993;2(4):417-23.

96. Fouz-Roson N, Montemayor-Rubio T, Almadana-Pacheco V, Montserrat-Garcia S, Gomez-Bastero AP, Romero-Munoz C, et al. Effect of 0.5 mg versus 1 mg varenicline for smoking cessation: a randomized controlled trial. Addiction. 2017;112(9):1610-9.

97. Fucito LM, Toll BA, Wu R, Romano DM, Tek E, O'Malley SS. A preliminary investigation of varenicline for heavy drinking smokers. Psychopharmacology (Berl). 2011;215(4):655-63.

98. Gariti P, Lynch K, Alterman A, Kampman K, Xie H, Varillo K. Comparing smoking treatment programs for lighter smokers with and without a history of heavier smoking. J Subst Abuse Treat. 2009;37(3):247-55.

99. Garvey AJ, Kinnunen T, Nordstrom BL, Utman CH, Doherty K, Rosner B, et al. Effects of nicotine gum dose by level of nicotine dependence. Nicotine Tob Res. 2000;2(1):53-63.

100. Garza D, Murphy M, Tseng LJ, Riordan HJ, Chatterjee A. A double-blind randomized placebo-controlled pilot study of neuropsychiatric adverse events in abstinent smokers treated with varenicline or placebo. Biol Psychiatry. 2011;69(11):1075-82.

101. George TP, Vessicchio JC, Termine A, Bregartner TA, Feingold A, Rounsaville BJ, et al. A placebo controlled trial of bupropion for smoking cessation in schizophrenia. Biol Psychiatry. 2002;52(1):53-61.

102. George TP, Vessicchio JC, Sacco KA, Weinberger AH, Dudas MM, Allen TM, et al. A placebo-controlled trial of bupropion combined with nicotine patch for smoking cessation in schizophrenia. Biol Psychiatry. 2008;63(11):1092-6.

103. Ginsberg D, Hall SM, Rosinski M. Partner support, psychological treatment, and nicotine gum in smoking treatment: an incremental study. Int J Addict. 1992;27(5):503-14.

104. Glavas D, Rumboldt M, Rumboldt Z. Smoking cessation with nicotine replacement therapy among health care workers: Randomized double-blind study. Croatian Medical Journal. 2003;44(2):219-24.

105. Glavas D, Rumboldt Z. [Smoking cessation using the transdermal nicotine system]. Lijec Vjesn. 2003;125(1-2):8-12.

106. GlaxoSmithKline. Efficacy and Safety Study of Nicotine Mint Lozenge (2mg and 4mg) in Smoking Cessation. https://ClinicalTrials.gov/show/NCT00985985; 2009.

107. GlaxoSmithKline. Provoked Craving Relief Study by NRT. https://ClinicalTrials.gov/show/NCT01476202; 2011.

108. Glover ED, Glover PN, Franzon M, Sullivan CR, Cerullo CC, Howell RM, et al. A comparison of a nicotine sublingual tablet and placebo for smoking cessation. Nicotine Tob Res. 2002;4(4):441-50.

109. Glover ED, Glover PN, Sullivan CR, Cerullo CL, Hobbs G. A comparison of sustained-release bupropion and placebo for smokeless tobacco cessation. Am J Health Behav. 2002;26(5):386-93.

110. Gonzales DH, Nides MA, Ferry LH, Kustra RP, Jamerson BD, Segall N, et al. Bupropion SR as an aid to smoking cessation in smokers treated previously with bupropion: a randomized placebo-controlled study. Clin Pharmacol Ther. 2001;69(6):438-44.

111. Gonzales D, Rennard SI, Nides M, Oncken C, Azoulay S, Billing CB, et al. Varenicline, an alpha4beta2 nicotinic acetylcholine receptor partial agonist, vs sustained-release bupropion and placebo for smoking cessation: a randomized controlled trial. JAMA. 2006;296(1):47-55.

112. Gonzales D, Hajek P, Pliamm L, Nackaerts K, Tseng LJ, McRae TD, et al. Retreatment with varenicline for smoking cessation in smokers who have previously taken varenicline: a randomized, placebo-controlled trial. Clin Pharmacol Ther. 2014;96(3):390-6.

113. Gourlay SG, Forbes A, Marriner T, Pethica D, McNeil JJ. Double blind trial of repeated treatment with transdermal nicotine for relapsed smokers. BMJ. 1995;311(7001):363-6.

114. Graham AL, Papandonatos GD, Cha S, Erar B, Amato MS, Cobb NK, et al. Improving Adherence to Smoking Cessation Treatment: Intervention Effects in a Web-Based Randomized Trial. Nicotine Tob Res. 2017;19(3):324-32.

115. Grant KM, Kelley SS, Smith LM, Agrawal S, Meyer JR, Romberger DJ. Bupropion and nicotine patch as smoking cessation aids in alcoholics. Alcohol. 2007;41(5):381-91.

116. Gray KM, McClure EA, Baker NL, Hartwell KJ, Carpenter MJ, Saladin ME. An exploratory short-term double-blind randomized trial of varenicline versus nicotine patch for smoking cessation in women. Addiction. 2015;110(6):1027-34.

117. Haarmann H, Gossler A, Herrmann P, Bonev S, Nguyen XP, Hasenfuss G, et al. Effects of varenicline on sympatho-vagal balance and cue reactivity during smoking withdrawal: a randomised placebo-controlled trial. Tob Induc Dis. 2016;14:26.

118. Haggstram FM, Chatkin JM, Sussenbach-Vaz E, Cesari DH, Fam CF, Fritscher CC. A controlled trial of nortriptyline, sustained-release bupropion and placebo for smoking cessation: preliminary results. Pulm Pharmacol Ther. 2006;19(3):205-9.

119. Hajek P, McRobbie HJ, Myers KE, Stapleton J, Dhanji AR. Use of varenicline for 4 weeks before quitting smoking: decrease in ad lib smoking and increase in smoking cessation rates. Arch Intern Med. 2011;171(8):770-7.

120. Hajek P, Smith KM, Dhanji AR, McRobbie H. Is a combination of varenicline and nicotine patch more effective in helping smokers quit than varenicline alone? A randomised controlled trial. BMC Med. 2013;11:140.

121. Hajek P, McRobbie H, Myers Smith K, Phillips A, Cornwall D, Dhanji AR. Increasing varenicline dose in smokers who do not respond to the standard dosage: a randomized clinical trial. JAMA Intern Med. 2015;175(2):266-71.

122. Hajek P, Phillips-Waller A, Przulj D, Pesola F, Myers Smith K, Bisal N, et al. A Randomized Trial of E-Cigarettes versus Nicotine-Replacement Therapy. N Engl J Med. 2019;380(7):629-37.

123. Hall SM, Humfleet GL, Munoz RF, Reus VI, Robbins JA, Prochaska JJ. Extended treatment of older cigarette smokers. Addiction. 2009;104(6):1043-52.

124. Hall SM, Humfleet GL, Munoz RF, Reus VI, Prochaska JJ, Robbins JA. Using extended cognitive behavioral treatment and medication to treat dependent smokers. Am J Public Health. 2011;101(12):2349-56.

125. Hand S, Edwards S, Campbell IA, Cannings R. Controlled trial of three weeks nicotine replacement treatment in hospital patients also given advice and support. Thorax. 2002;57(8):715-8.

126. Hanioka T, Ojima M, Tanaka H, Naito M, Hamajima N, Matsuse R. Intensive smoking-cessation intervention in the dental setting. J Dent Res. 2010;89(1):66-70.

127. Harackiewicz JM, Blair LW, Sansone C, Epstein JA, Stuchell RN. Nicotine gum and self-help manuals in smoking cessation: an evaluation in a medical context. Addict Behav. 1988;13(4):319-30.

128. Hatsukami DK, Rennard S, Patel MK, Kotlyar M, Malcolm R, Nides MA, et al. Effects of sustained-release bupropion among persons interested in reducing but not quitting smoking. Am J Med. 2004;116(3):151-7.

129. Hawk LW, Jr., Ashare RL, Lohnes SF, Schlienz NJ, Rhodes JD, Tiffany ST, et al. The effects of extended pre-quit varenicline treatment on smoking behavior and short-term abstinence: a randomized clinical trial. Clin Pharmacol Ther. 2012;91(2):172-80.

130. Hays JT, Croghan IT, Schroeder DR, Offord KP, Hurt RD, Wolter TD, et al. Over-the-counter nicotine patch therapy for smoking cessation: results from randomized, double-blind, placebo-controlled, and open label trials. Am J Public Health. 1999;89(11):1701-7.

131. Hays JT, Hurt RD, Rigotti NA, Niaura R, Gonzales D, Durcan MJ, et al. Sustained-release bupropion for pharmacologic relapse prevention after smoking cessation. a randomized, controlled trial. Ann Intern Med. 2001;135(6):423-33.

132. Hays JT, Hurt RD, Decker PA, Croghan IT, Offord KP, Patten CA. A randomized, controlled trial of bupropion sustained-release for preventing tobacco relapse in recovering alcoholics. Nicotine Tob Res. 2009;11(7):859-67.

133. Herrmann ES, Cooper ZD, Bedi G, Ramesh D, Reed SC, Comer SD, et al. Varenicline and nabilone in tobacco and cannabis co-users: effects on tobacco abstinence, withdrawal and a laboratory model of cannabis relapse. Addict Biol. 2019;24(4):765-76.

134. Hertzberg MA, Moore SD, Feldman ME, Beckham JC. A preliminary study of bupropion sustained-release for smoking cessation in patients with chronic posttraumatic stress disorder. J Clin Psychopharmacol. 2001;21(1):94-8.

135. Heydari G, Talischi F, Tafti SF, Masjedi MR. Quitting smoking with varenicline: parallel, randomised efficacy trial in Iran. Int J Tuberc Lung Dis. 2012;16(2):268-72.

136. Hilberink SR, Jacobs JE, Breteler MH, de Vries H, Grol RP. General practice counseling for patients with chronic obstructive pulmonary disease to quit smoking: impact after 1 year of two complex interventions. Patient Educ Couns. 2011;83(1):120-4.

137. Hilleman DE, Mohiuddin SM, Delcore MG. Comparison of fixed-dose transdermal nicotine, tapered-dose transdermal nicotine, and buspirone in smoking cessation. J Clin Pharmacol. 1994;34(3):222-4.

138. Hjalmarson AI. Effect of nicotine chewing gum in smoking cessation. A randomized, placebo-controlled, double-blind study. JAMA. 1984;252(20):2835-8.

139. Hjalmarson A, Franzon M, Westin A, Wiklund O. Effect of nicotine nasal spray on smoking cessation. A randomized, placebo-controlled, double-blind study. Arch Intern Med. 1994;154(22):2567-72.

140. Hjalmarson A, Nilsson F, Sjostrom L, Wiklund O. The nicotine inhaler in smoking cessation. Arch Intern Med. 1997;157(15):1721-8.

141. Holt S, Timu-Parata C, Ryder-Lewis S, Weatherall M, Beasley R. Efficacy of bupropion in the indigenous Maori population in New Zealand. Thorax. 2005;60(2):120-3.

142. Hong LE, Thaker GK, McMahon RP, Summerfelt A, Rachbeisel J, Fuller RL, et al. Effects of moderate-dose treatment with varenicline on neurobiological and cognitive biomarkers in smokers and nonsmokers with schizophrenia or schizoaffective disorder. Arch Gen Psychiatry. 2011;68(12):1195-206.

143. Howard-Pitney B, Killen JD, Fortmann SP. Quitting chew: results from a randomized trial using nicotine patches. Exp Clin Psychopharmacol. 1999;7(4):362-71.

144. Hughes JR, Gust SW, Keenan RM, Fenwick JW, Healey ML. Nicotine vs placebo gum in general medical practice. JAMA. 1989;261(9):1300-5.

145. Hughes JR, Lesmes GR, Hatsukami DK, Richmond RL, Lichtenstein E, Jorenby DE, et al. Are higher doses of nicotine replacement more effective for smoking cessation? Nicotine Tob Res. 1999;1(2):169-74.

146. Hughes JR, Novy P, Hatsukami DK, Jensen J, Callas PW. Efficacy of nicotine patch in smokers with a history of alcoholism. Alcohol Clin Exp Res. 2003;27(6):946-54.

147. Hughes JR, Solomon LJ, Livingston AE, Callas PW, Peters EN. A randomized, controlled trial of NRT-aided gradual vs. abrupt cessation in smokers actively trying to quit. Drug Alcohol Depend. 2010;111(1-2):105-13.

148. Hughes JR, Rennard SI, Fingar JR, Talbot SK, Callas PW, Fagerstrom KO. Efficacy of varenicline to prompt quit attempts in smokers not currently trying to quit: a randomized placebo-controlled trial. Nicotine Tob Res. 2011;13(10):955-64.

149. Hurt RD, Lauger GG, Offord KP, Kottke TE, Dale LC. Nicotine-Replacement Therapy with Use of a Transdermal Nicotine Patch - a Randomized Double-Blind Placebo-Controlled Trial. Mayo Clinic Proceedings. 1990;65(12):1529-37.

150. Hurt RD, Dale LC, Fredrickson PA, Caldwell CC, Lee GA, Offord KP, et al. Nicotine patch therapy for smoking cessation combined with physician advice and nurse follow-up. One-year outcome and percentage of nicotine replacement. JAMA. 1994;271(8):595-600.

151. Hurt RD, Sachs DP, Glover ED, Offord KP, Johnston JA, Dale LC, et al. A comparison of sustained-release bupropion and placebo for smoking cessation. N Engl J Med. 1997;337(17):1195-202.

152. Hurt RD, Krook JE, Croghan IT, Loprinzi CL, Sloan JA, Novotny PJ, et al. Nicotine patch therapy based on smoking rate followed by bupropion for prevention of relapse to smoking. J Clin Oncol. 2003;21(5):914-20.

153. Hurt RT, Ebbert JO, Croghan IT, Schroeder DR, Hurt RD, Hays JT. Varenicline for tobacco-dependence treatment in alcohol-dependent smokers: A randomized controlled trial. Drug Alcohol Depend. 2018;184:12-7.

154. Randomised trial of nicotine patches in general practice: results at one year. Imperial Cancer Research Fund General Practice Research Group. BMJ. 1994;308(6942):1476-7.

155. Ikonomidis I, Marinou M, Vlastos D, Kourea K, Andreadou I, Liarakos N, et al. Effects of varenicline and nicotine replacement therapy on arterial elasticity, endothelial glycocalyx and oxidative stress during a 3-month smoking cessation program. Atherosclerosis. 2017;262:123-30.

156. Jain R, Jhanjee S, Jain V, Gupta T, Mittal S, Goelz P, et al. A double-blind placebo-controlled randomized trial of varenicline for smokeless tobacco dependence in India. Nicotine Tob Res. 2014;16(1):50-7.

157. Jamrozik K, Fowler G, Vessey M, Wald N. Placebo controlled trial of nicotine chewing gum in general practice. Br Med J (Clin Res Ed). 1984;289(6448):794-7.

158. Jarvis MJ, Raw M, Russell MAH, Feyerabend C. Randomized Controlled Trial of Nicotine Chewing-Gum. British Medical Journal. 1982;285(6341):537-40.

159. Jennings C, Kotseva K, De Bacquer D, Hoes A, de Velasco J, Brusaferro S, et al. Effectiveness of a preventive cardiology programme for high CVD risk persistent smokers: the EUROACTION PLUS varenicline trial. Eur Heart J. 2014;35(21):1411-20.

160. Jorenby DE, Smith SS, Fiore MC, Hurt RD, Offord KP, Croghan IT, et al. Varying nicotine patch dose and type of smoking cessation counseling. JAMA. 1995;274(17):1347-52.

161. Jorenby DE, Leischow SJ, Nides MA, Rennard SI, Johnston JA, Hughes AR, et al. A controlled trial of sustained-release bupropion, a nicotine patch, or both for smoking cessation. N Engl J Med. 1999;340(9):685-91.

162. Jorenby DE, Hays JT, Rigotti NA, Azoulay S, Watsky EJ, Williams KE, et al. Efficacy of varenicline, an alpha4beta2 nicotinic acetylcholine receptor partial agonist, vs placebo or sustained-release bupropion for smoking cessation: a randomized controlled trial. JAMA. 2006;296(1):56-63.

163. Joseph AM, Norman SM, Ferry LH, Prochazka AV, Westman EC, Steele BG, et al. The safety of transdermal nicotine as an aid to smoking cessation in patients with cardiac disease. N Engl J Med. 1996;335(24):1792-8.

164. Joseph AM, Fu SS, Lindgren B, Rothman AJ, Kodl M, Lando H, et al. Chronic disease management for tobacco dependence: a randomized, controlled trial. Arch Intern Med. 2011;171(21):1894-900.

165. Kalman D, Kahler CW, Garvey AJ, Monti PM. High-dose nicotine patch therapy for smokers with a history of alcohol dependence: 36-week outcomes. J Subst Abuse Treat. 2006;30(3):213-7.

166. Kalman D, Herz L, Monti P, Kahler CW, Mooney M, Rodrigues S, et al. Incremental efficacy of adding bupropion to the nicotine patch for smoking cessation in smokers with a recent history of alcohol dependence: results from a randomized, double-blind, placebo-controlled study. Drug Alcohol Depend. 2011;118(2-3):111-8.

167. Karam-Hage M, Strobbe S, Robinson JD, Brower KJ. Bupropion-SR for smoking cessation in early recovery from alcohol dependence: a placebo-controlled, double-blind pilot study. Am J Drug Alcohol Abuse. 2011;37(6):487-90.

168. Killen JD, Maccoby N, Taylor CB. Nicotine Gum and Self-Regulation Training in Smoking Relapse Prevention. Behavior Therapy. 1984;15(3):234-48.

169. Killen JD, Fortmann SP, Davis L, Strausberg L, Varady A. Do heavy smokers benefit from higher dose nicotine patch therapy? Experimental and Clinical Psychopharmacology. 1999;7(3):226-33.

170. Killen JD, Fortmann SP, Murphy GM, Jr., Hayward C, Arredondo C, Cromp D, et al. Extended treatment with bupropion SR for cigarette smoking cessation. J Consult Clin Psychol. 2006;74(2):286-94.

171. Klesges RC, Ebbert JO, Talcott GW, Thomas F, Richey PA, Womack C, et al. Efficacy of a Tobacco Quitline in Active Duty Military and TRICARE Beneficiaries: A Randomized Trial. Mil Med. 2015;180(8):917-25.

172. Koegelenberg CF, Noor F, Bateman ED, van Zyl-Smit RN, Bruning A, O'Brien JA, et al. Efficacy of varenicline combined with nicotine replacement therapy vs varenicline alone for smoking cessation: a randomized clinical trial. JAMA. 2014;312(2):155-61.

173. Kornitzer M, Boutsen M, Dramaix M, Thijs J, Gustavsson G. Combined use of nicotine patch and gum in smoking cessation: a placebo-controlled clinical trial. Prev Med. 1995;24(1):41-7.

174. Kralikova E, Kozak J, Rasmussen T, Cort N. The clinical benefits of NRT-supported smoking reduction. Nicotine Tob Res. 2002;4(243):20.

175. Kupecz D, Prochazka A. A comparison of nicotine delivery systems in a multimodality smoking cessation program. Nurse Pract. 1996;21(2):73, 7-8, 81 passim.

176. Lee SM, Landry J, Jones PM, Buhrmann O, Morley-Forster P. The effectiveness of a perioperative smoking cessation program: a randomized clinical trial. Anesth Analg. 2013;117(3):605-13.

177. Lee SM, Tenney R, Wallace AW, Arjomandi M. E-cigarettes versus nicotine patches for perioperative smoking cessation: a pilot randomized trial. PeerJ. 2018;6:e5609.

178. Leischow SJ, Nilsson F, Franzon M, Hill A, Otte P, Merikle EP. Efficacy of the nicotine inhaler as an adjunct to smoking cessation. American Journal of Health Behavior. 1996;20(5):364-71.

179. Lerman C, Kaufmann V, Rukstalis M, Patterson F, Perkins K, Audrain-McGovern J, et al. Individualizing nicotine replacement therapy for the treatment of tobacco dependence: a randomized trial. Ann Intern Med. 2004;140(6):426-33.

180. Lerman C, Schnoll RA, Hawk LW, Jr., Cinciripini P, George TP, Wileyto EP, et al. Use of the nicotine metabolite ratio as a genetically informed biomarker of response to nicotine patch or varenicline for smoking cessation: a randomised, double-blind placebo-controlled trial. Lancet Respir Med. 2015;3(2):131-8.

181. Levin ED, Westman EC, Stein RM, Carnahan E, Sanchez M, Herman S, et al. Nicotine skin patch treatment increases abstinence, decreases withdrawal symptoms, and attenuates rewarding effects of smoking. J Clin Psychopharmacol. 1994;14(1):41-9.

182. Levine MD, Perkins KA, Kalarchian MA, Cheng Y, Houck PR, Slane JD, et al. Bupropion and cognitive behavioral therapy for weight-concerned women smokers. Arch Intern Med. 2010;170(6):543-50.

183. Lewis SF, Piasecki TM, Fiore MC, Anderson JE, Baker TB. Transdermal nicotine replacement for hospitalized patients: a randomized clinical trial. Prev Med. 1998;27(2):296-303.

184. Li J, Zhang T-l, Wang B, Li X-w. An efficacy analysis of bupropion for smoking cessation in schizophrenia. Zhongguo Xinyao yu Linchuang Zazhi. 2009;28(3):231-4.

185. Lindson-Hawley N, Coleman T, Docherty G, Hajek P, Lewis S, Lycett D, et al. Nicotine patch preloading for smoking cessation (the preloading trial): study protocol for a randomized controlled trial. Trials. 2014;15(296):296.

186. Litten RZ, Ryan ML, Fertig JB, Falk DE, Johnson B, Dunn KE, et al. A double-blind, placebo-controlled trial assessing the efficacy of varenicline tartrate for alcohol dependence. J Addict Med. 2013;7(4):277-86.

187. Littlewood RA, Claus ED, Wilcox CE, Mickey J, Arenella PB, Bryan AD, et al. Moderators of smoking cessation outcomes in a randomized-controlled trial of varenicline versus placebo. Psychopharmacology (Berl). 2017;234(23-24):3417-29.

188. Llivina TS, Tuya DM, Quintana JG, Torres CI, Barrera EC, Saez CM, et al. Smoking Cessation - Effectiveness of Nicotine Containing Chewing Gum - a Double-Blind Trial. Medicina Clinica. 1988;90(16):646-50.

189. Marsh HS, Dresler CM, Choi JH, Targett DA, Gamble ML, Strahs KR. Safety profile of a nicotine lozenge compared with that of nicotine gum in adult smokers with underlying medical conditions: a 12-week, randomized, open-label study. Clin Ther. 2005;27(10):1571-87.

190. Masiero M, Lucchiari C, Mazzocco K, Veronesi G, Maisonneuve P, Jemos C, et al. E-cigarettes May Support Smokers With High Smoking-Related Risk Awareness to Stop Smoking in the Short Run: Preliminary Results by Randomized Controlled Trial. Nicotine Tob Res. 2019;21(1):119-26.

191. McCarthy DE, Piasecki TM, Lawrence DL, Jorenby DE, Shiffman S, Fiore MC, et al. A randomized controlled clinical trial of bupropion SR and individual smoking cessation counseling. Nicotine Tob Res. 2008;10(4):717-29.

192. McClure EA, Vandrey RG, Johnson MW, Stitzer ML. Effects of varenicline on abstinence and smoking reward following a programmed lapse. Nicotine Tob Res. 2013;15(1):139-48.

193. McKee SA, Harrison EL, O'Malley SS, Krishnan-Sarin S, Shi J, Tetrault JM, et al. Varenicline reduces alcohol self-administration in heavy-drinking smokers. Biol Psychiatry. 2009;66(2):185-90.

194. Mercie P, Arsandaux J, Katlama C, Ferret S, Beuscart A, Spadone C, et al. Efficacy and safety of varenicline for smoking cessation in people living with HIV in France (ANRS 144 Inter-ACTIV): a randomised controlled phase 3 clinical trial. Lancet Hiv. 2018;5(3):E126-E35.

195. Merz PG, Kellerstanislawski B, Huber T, Woodcock BG, Rietbrock N. Transdermal Nicotine in Smoking Cessation and Involvement of Nonspecific Influences. International Journal of Clinical Pharmacology and Therapeutics. 1993;31(10):476-82.

196. Meszaros ZS, Abdul-Malak Y, Dimmock JA, Wang D, Ajagbe TO, Batki SL. Varenicline treatment of concurrent alcohol and nicotine dependence in schizophrenia: a randomized, placebo-controlled pilot trial. J Clin Psychopharmacol. 2013;33(2):243-7.

197. Mitchell JM, Teague CH, Kayser AS, Bartlett SE, Fields HL. Varenicline decreases alcohol consumption in heavy-drinking smokers. Psychopharmacology (Berl). 2012;223(3):299-306.

198. Moller AM, Villebro N, Pedersen T, Tonnesen H. Effect of preoperative smoking intervention on postoperative complications: a randomised clinical trial. Lancet. 2002;359(9301):114-7.

199. Molyneux A, Lewis S, Leivers U, Anderton A, Antoniak M, Brackenridge A, et al. Clinical trial comparing nicotine replacement therapy (NRT) plus brief counselling, brief counselling alone, and minimal intervention on smoking cessation in hospital inpatients. Thorax. 2003;58(6):484-8.

200. Mori T STYGNMHT. A clinical trial of nicotine chewing gum for smoking cessation [abstract 428]. 1992.

201. Mulligan SC, Masterson JG, Devane JG, Kelly JG. Clinical and pharmacokinetic properties of a transdermal nicotine patch. Clin Pharmacol Ther. 1990;47(3):331-7.

202. Myles PS, Leslie K, Angliss M, Mezzavia P, Lee L. Effectiveness of bupropion as an aid to stopping smoking before elective surgery: a randomised controlled trial. Anaesthesia. 2004;59(11):1053-8.

203. Myung SK, Seo HG, Park S, Kim Y, Kim DJ, Lee DH, et al. Sociodemographic and smoking behavioral predictors associated with smoking cessation according to follow-up periods: a randomized, double-blind, placebo-controlled trial of transdermal nicotine patches. J Korean Med Sci. 2007;22(6):1065-70.

204. Nahvi S, Ning Y, Segal KS, Richter KP, Arnsten JH. Varenicline efficacy and safety among methadone maintained smokers: a randomized placebo-controlled trial. Addiction. 2014;109(9):1554-63.

205. Nakamura M, Oshima A, Fujimoto Y, Maruyama N, Ishibashi T, Reeves KR. Efficacy and tolerability of varenicline, an alpha4beta2 nicotinic acetylcholine receptor partial agonist, in a 12-week, randomized, placebo-controlled, dose-response study with 40-week follow-up for smoking cessation in Japanese smokers. Clin Ther. 2007;29(6):1040-56.

206. Fox Chase Cancer Center, National Cancer Institute. Counseling and Nicotine Replacement Therapy in Helping Adult Smokers Quit Smoking. https://ClinicalTrials.gov/show/NCT00365508; 2006.

207. Pfizer. Long-term Varenicline Treatment for Smoking Cessation. University of Wisconsin, Madison: https://ClinicalTrials.gov/show/NCT00828113; 2009.

208. Trial of Nicotine Nasal Spray as an Aid for Smoking Cessation in Schizophrenia. https://ClinicalTrials.gov/show/NCT01010477; 2009.

209. Duke University. Supplemental Nicotine Administration for Smoking Cessation in Posttraumatic Stress Disorder (PTSD). 2009.

210. Niaura R, Hays JT, Jorenby DE, Leone FT, Pappas JE, Reeves KR, et al. The efficacy and safety of varenicline for smoking cessation using a flexible dosing strategy in adult smokers: a randomized controlled trial. Curr Med Res Opin. 2008;24(7):1931-41.

211. Nides M, Oncken C, Gonzales D, Rennard S, Watsky EJ, Anziano R, et al. Smoking cessation with varenicline, a selective alpha4beta2 nicotinic receptor partial agonist: results from a 7-week, randomized, placebo- and bupropion-controlled trial with 1-year follow-up. Arch Intern Med. 2006;166(15):1561-8.

212. Nides M, Danielsson T, Saunders F, Perfekt R, Kapikian R, Solla J, et al. Efficacy and safety of a nicotine mouth spray for smoking cessation; a randomized, multicenter, controlled study in a naturalistic setting. Nicotine Tob Res. 2018;18:18.

213. Nides M, Shanga GM, Bishop A, Becker WD. Nicotine Lozenges in the Relief of Behaviorally Provoked Craving. Am J Health Behav. 2018;42(3):69-80.

214. Olson LC, Hong D, Conell-Price JS, Cheng S, Flood P. A transdermal nicotine patch is not effective for postoperative pain management in smokers: a pilot dose-ranging study. Anesth Analg. 2009;109(6):1987-91.

215. O'Malley S, Zweben A, Fucito L, Wu R, Piepmeier M, Ockert D, et al. Effect of Varenicline Combined With Medical Management on Alcohol Use Disorder With Comorbid Cigarette Smoking: a Randomized Clinical Trial2017; 75(2):[129-38 pp.]. Available from: http://cochranelibrary-wiley.com/o/cochrane/clcentral/articles/299/CN-01457299/frame.html.

216. Oncken C, Gonzales D, Nides M, Rennard S, Watsky E, Billing CB, et al. Efficacy and safety of the novel selective nicotinic acetylcholine receptor partial agonist, varenicline, for smoking cessation. Arch Intern Med. 2006;166(15):1571-7.

217. Oncken C, Cooney J, Feinn R, Lando H, Kranzler HR. Transdermal nicotine for smoking cessation in postmenopausal women. Addict Behav. 2007;32(2):296-309.

218. Pack QR, Jorenby DE, Fiore MC, Jackson T, Weston P, Piper ME, et al. A comparison of the nicotine lozenge and nicotine gum: an effectiveness randomized controlled trial. WMJ. 2008;107(5):237-43.

219. Paoletti P, Fornai E, Maggiorelli F, Puntoni R, Viegi G, Carrozzi L, et al. Importance of baseline cotinine plasma values in smoking cessation: results from a double-blind study with nicotine patch. Eur Respir J. 1996;9(4):643-51.

220. Pei H, Zhang LJ, Zeng LM, Yu F. Effect of preoperative smoking intervention on postoperative complications of total hip replacement. [Chinese]. Chinese Journal of Evidence-Based Medicine. 2014;14(4):399-403.

221. Perng RP, Hsieh WC, Chen YM, Lu CC, Chiang SJ. Randomized, double-blind, placebo-controlled study of transdermal nicotine patch for smoking cessation. J Formos Med Assoc. 1998;97(8):547-51.

222. Piper ME, Smith SS, Schlam TR, Fiore MC, Jorenby DE, Fraser D, et al. A randomized placebo-controlled clinical trial of 5 smoking cessation pharmacotherapies. Arch Gen Psychiatry. 2009;66(11):1253-62.

223. Piper ME, Cook JW, Schlam TR, Jorenby DE, Smith SS, Collins LM, et al. A Randomized Controlled Trial of an Optimized Smoking Treatment Delivered in Primary Care. Ann Behav Med. 2018;52(10):854-64.

224. Planer D, Lev I, Elitzur Y, Sharon N, Ouzan E, Pugatsch T, et al. Bupropion for smoking cessation in patients with acute coronary syndrome. Arch Intern Med. 2011;171(12):1055-60.

225. Plebani JG, Lynch KG, Rennert L, Pettinati HM, O'Brien CP, Kampman KM. Results from a pilot clinical trial of varenicline for the treatment of alcohol dependence. Drug Alcohol Depend. 2013;133(2):754-8.

226. Poling J, Rounsaville B, Gonsai K, Severino K, Sofuoglu M. The safety and efficacy of varenicline in cocaine using smokers maintained on methadone: a pilot study. Am J Addict. 2010;19(5):401-8.

227. Ponciano-Rodriguez G, Paez-Martinez N, Villa-Romero A, Gutierrez-Grobe Y, Mendez-Sanchez N. Early changes in the components of the metabolic syndrome in a group of smokers after tobacco cessation. Metab Syndr Relat Disord. 2014;12(4):242-50.

228. Puska P, Bjorkqvist S, Koskela K. Nicotine-containing chewing gum in smoking cessation: a double blind trial with half year follow-up. Addict Behav. 1979;4(2):141-6.

229. Puska P, Korhonen HJ, Vartiainen E, Urjanheimo EL, Gustavsson G, Westin A. Combined use of nicotine patch and gum compared with gum alone in smoking cessation - a clinical trial in North Karelia. Tobacco Control. 1995;4(3):231-5.

230. Ramon JM, Morchon S, Baena A, Masuet-Aumatell C. Combining varenicline and nicotine patches: a randomized controlled trial study in smoking cessation. BMC Med. 2014;12(172):172.

231. Ratner PA, Johnson JL, Richardson CG, Bottorff JL, Moffat B, Mackay M, et al. Efficacy of a smoking-cessation intervention for elective-surgical patients. Res Nurs Health. 2004;27(3):148-61.

232. Ray LA, Courtney KE, Ghahremani DG, Miotto K, Brody A, London ED. Varenicline, low dose naltrexone, and their combination for heavy-drinking smokers: human laboratory findings. Psychopharmacology (Berl). 2014;231(19):3843-53.

233. Reid R, Pipe A, Higginson L, Johnson K, D'Angelo MS, Cooke D, et al. Stepped care approach to smoking cessation in patients hospitalized for coronary artery disease. J Cardiopulm Rehabil. 2003;23(3):176-82.

234. Rennard S, Daughton D, Cheney R, Thompson A, Miles R, Windle J, et al. Nicotine Replacement Therapy for Patients with Coronary-Artery Disease. Archives of Internal Medicine. 1994;154(9):989-95.

235. Rennard SI, Glover ED, Leischow S, Daughton DM, Glover PN, Muramoto M, et al. Efficacy of the nicotine inhaler in smoking reduction: A double-blind, randomized trial. Nicotine Tob Res. 2006;8(4):555-64.

236. Rennard S, Hughes J, Cinciripini PM, Kralikova E, Raupach T, Arteaga C, et al. A randomized placebo-controlled trial of varenicline for smoking cessation allowing flexible quit dates. Nicotine Tob Res. 2012;14(3):343-50.

237. Richmond RL, Harris K, Dealmeidaneto A. The Transdermal Nicotine Patch - Results of a Randomized Placebo-Controlled Trial. Medical Journal of Australia. 1994;161(2):130-&.

238. Rigotti NA, Thorndike AN, Regan S, McKool K, Pasternak RC, Chang Y, et al. Bupropion for smokers hospitalized with acute cardiovascular disease. Am J Med. 2006;119(12):1080-7.

239. Rigotti NA, Pipe AL, Benowitz NL, Arteaga C, Garza D, Tonstad S. Efficacy and safety of varenicline for smoking cessation in patients with cardiovascular disease: a randomized trial. Circulation. 2010;121(2):221-9.

240. Rodriguez-Artalejo F, Lafuente Urdinguio P, Guallar-Castillon P, Garteizaurrekoa Dublang P, Sainz Martinez O, Diez Azcarate JI, et al. One year effectiveness of an individualised smoking cessation intervention at the workplace: a randomised controlled trial. Occup Environ Med. 2003;60(5):358-63.

241. Rohsenow DJ, Tidey JW, Martin RA, Colby SM, Swift RM, Leggio L, et al. Varenicline versus nicotine patch with brief advice for smokers with substance use disorders with or without depression: effects on smoking, substance use and depressive symptoms. Addiction. 2017;112(10):1808-20.

242. Rose JE, Levin ED, Behm FM, Adivi C, Schur C. Transdermal nicotine facilitates smoking cessation. Clin Pharmacol Ther. 1990;47(3):323-30.

243. Rose JE, Herskovic JE, Behm FM, Westman EC. Precessation treatment with nicotine patch significantly increases abstinence rates relative to conventional treatment. Nicotine Tob Res. 2009;11(9):1067-75.

244. Rose JE, Behm FM. Adapting smoking cessation treatment according to initial response to precessation nicotine patch. Am J Psychiatry. 2013;170(8):860-7.

245. Rose JE, Behm FM. Combination treatment with varenicline and bupropion in an adaptive smoking cessation paradigm. Am J Psychiatry. 2014;171(11):1199-205.

246. Rose JE, Behm FM. Combination Varenicline/Bupropion Treatment Benefits Highly Dependent Smokers in an Adaptive Smoking Cessation Paradigm. Nicotine Tob Res. 2017;19(8):999-1002.

247. Rovina N, Nikoloutsou I, Katsani G, Dima E, Fransis K, Roussos C, et al. Effectiveness of pharmacotherapy and behavioral interventions for smoking cessation in actual clinical practice. Ther Adv Respir Dis. 2009;3(6):279-87.

248. Rungruanghiranya S, Ekpanyaskul C, Hattapornsawan Y, Tundulawessa Y. Effect of nicotine polyestex gum on smoking cessation and quality of life. J Med Assoc Thai. 2008;91(11):1656-62.

249. Russell MAH, Merriman R, Stapleton J, Taylor W. Effect of Nicotine Chewing Gum as an Adjunct to General-Practitioners Advice against Smoking. British Medical Journal. 1983;287(6407):1782-5.

250. Rutgers TSUoNJ, Pfizer. Varenicline-Aided Cigarette Reduction in Smokers Not Ready to Quit. https://ClinicalTrials.gov/show/NCT01308736; 2011.

251. Sachs DPL, Sawe U, Leischow SJ. Effectiveness of a 16-Hour Transdermal Nicotine Patch in a Medical-Practice Setting, without Intensive Group-Counseling. Archives of Internal Medicine. 1993;153(16):1881-90.

252. Sadr Azodi O, Lindstrom D, Adami J, Tonnesen H, Nasell H, Gilljam H, et al. The efficacy of a smoking cessation programme in patients undergoing elective surgery: a randomised clinical trial. Anaesthesia. 2009;64(3):259-65.

253. Schacht JP, Anton RF, Randall PK, Li X, Henderson S, Myrick H. Varenicline effects on drinking, craving and neural reward processing among non-treatment-seeking alcohol-dependent individuals. Psychopharmacology (Berl). 2014;231(18):3799-807.

254. Schmitz JM, Stotts AL, Mooney ME, Delaune KA, Moeller GF. Bupropion and cognitive-behavioral therapy for smoking cessation in women. Nicotine Tob Res. 2007;9(6):699-709.

255. Schneider NG, Jarvik ME, Forsythe AB, Read LL, Elliott ML, Schweiger A. Nicotine gum in smoking cessation: a placebo-controlled, double-blind trial. Addict Behav. 1983;8(3):253-61.

256. Schneider NG, Olmstead R, Mody FV, Doan K, Franzon M, Jarvik ME, et al. Efficacy of a nicotine nasal spray in smoking cessation: a placebo-controlled, double-blind trial. Addiction. 1995;90(12):1671-82.

257. Schneider NG, Olmstead R, Nilsson F, Mody FV, Franzon M, Doan K. Efficacy of a nicotine inhaler in smoking cessation: a double-blind, placebo-controlled trial. Addiction. 1996;91(9):1293-306.

258. Schnoll RA, Patterson F, Wileyto EP, Heitjan DF, Shields AE, Asch DA, et al. Effectiveness of extended-duration transdermal nicotine therapy: a randomized trial. Ann Intern Med. 2010;152(3):144-51.

259. Schnoll RA, Martinez E, Tatum KL, Glass M, Bernath A, Ferris D, et al. Nicotine patch vs. nicotine lozenge for smoking cessation: an effectiveness trial coordinated by the Community Clinical Oncology Program. Drug Alcohol Depend. 2010;107(2-3):237-43.

260. Schnoll RA, Wileyto EP, Leone FT, Tyndale RF, Benowitz NL. High dose transdermal nicotine for fast metabolizers of nicotine: a proof of concept placebo-controlled trial. Nicotine Tob Res. 2013;15(2):348-54.

261. Schnoll RA, Goelz PM, Veluz-Wilkins A, Blazekovic S, Powers L, Leone FT, et al. Long-term nicotine replacement therapy: a randomized clinical trial. JAMA Intern Med. 2015;175(4):504-11.

262. Schnoll R, Leone F, Veluz-Wilkins A, Miele A, Hole A, Jao NC, et al. A randomized controlled trial of 24 weeks of varenicline for tobacco use among cancer patients: Efficacy, safety, and adherence. Psychooncology. 2019;28(3):561-9.

263. Schuurmans MM, Diacon AH, van Biljon X, Bolliger CT. Effect of pre-treatment with nicotine patch on withdrawal symptoms and abstinence rates in smokers subsequently quitting with the nicotine patch: a randomized controlled trial. Addiction. 2004;99(5):634-40.

264. Selby P BBSN. Retreatment with ZYban SR: 52 week follow-up of a Canadian Multicentre trial (POS3-63). 2003.

265. Selma Bozkurt Z, Serkan Z, Esra K, Esra Aydin S. Comparison of the effectiveness of varenicline, extended-release bupropion and nicotine replacement therapy on the success and the maintenance of a smoking cessation program. Klinik Psikofarmakoloji Bulteni-Bulletin of Clinical Psychopharmacology. 2013;23(3):224-30.

266. Severson HH, Danaher BG, Ebbert JO, van Meter N, Lichtenstein E, Widdop C, et al. Randomized trial of nicotine lozenges and phone counseling for smokeless tobacco cessation. Nicotine Tob Res. 2015;17(3):309-15.

267. Sharma SK, Mohan A, Singh AD, Mishra H, Jhanjee S, Pandey RM, et al. Impact of nicotine replacement therapy as an adjunct to anti-tuberculosis treatment and behaviour change counselling in newly diagnosed pulmonary tuberculosis patients: an open-label, randomised controlled trial. Sci Rep. 2018;8(1):8828.

268. Sheng LX, Tang YL, Jiang ZN, Yao CH, Gao JY, Xu GZ, et al. Sustained-release bupropion for smoking cessation in a Chinese sample: a double-blind, placebo-controlled, randomized trial. Nicotine Tob Res. 2013;15(2):320-5.

269. Sherman SE, Aldana I, Estrada M, York L. Comparing the tolerability and effectiveness of two treatment regimens in a smoking clinic. Mil Med. 2008;173(6):550-4.

270. Shiffman S, Dresler CM, Hajek P, Gilburt SJ, Targett DA, Strahs KR. Efficacy of a nicotine lozenge for smoking cessation. Arch Intern Med. 2002;162(11):1267-76.

271. Shiffman S, Scharf DM, Shadel WG, Gwaltney CJ, Dang Q, Paton SM, et al. Analyzing milestones in smoking cessation: illustration in a nicotine patch trial in adult smokers. J Consult Clin Psychol. 2006;74(2):276-85.

272. Shiffman S, Ferguson SG, Strahs KR. Quitting by gradual smoking reduction using nicotine gum: a randomized controlled trial. Am J Prev Med. 2009;36(2):96-104 e1.

273. Shim JC, Jung DU, Jung SS, Seo YS, Cho DM, Lee JH, et al. Adjunctive varenicline treatment with antipsychotic medications for cognitive impairments in people with schizophrenia: a randomized double-blind placebo-controlled trial. Neuropsychopharmacology. 2012;37(3):660-8.

274. Shoptaw S, Heinzerling KG, Rotheram-Fuller E, Steward T, Wang J, Swanson AN, et al. Randomized, placebo-controlled trial of bupropion for the treatment of methamphetamine dependence. Drug Alcohol Depend. 2008;96(3):222-32.

275. Siddiqi K, Khan A, Ahmad M, Dogar O, Kanaan M, Newell JN, et al. Action to stop smoking in suspected tuberculosis (ASSIST) in Pakistan: a cluster randomized, controlled trial. Ann Intern Med. 2013;158(9):667-75.

276. Simon JA, Solkowitz SN, Carmody TP, Browner WS. Smoking cessation after surgery. A randomized trial. Arch Intern Med. 1997;157(12):1371-6.

277. Simon JA, Duncan C, Carmody TP, Hudes ES. Bupropion for smoking cessation: a randomized trial. Arch Intern Med. 2004;164(16):1797-803.

278. Simon JA, Duncan C, Huggins J, Solkowitz S, Carmody TP. Sustained-release bupropion for hospital-based smoking cessation: a randomized trial. Nicotine Tob Res. 2009;11(6):663-9.

279. Singh P, Kumar R. Assessment of the effectiveness of sustained release Bupropion and intensive physician advice in smoking cessation. Lung India. 2010;27(1):11-8.

280. Smith SS, McCarthy DE, Japuntich SJ, Christiansen B, Piper ME, Jorenby DE, et al. Comparative effectiveness of 5 smoking cessation pharmacotherapies in primary care clinics. Arch Intern Med. 2009;169(22):2148-55.

281. Smith RC, Amiaz R, Si TM, Maayan L, Jin H, Boules S, et al. Varenicline Effects on Smoking, Cognition, and Psychiatric Symptoms in Schizophrenia: A Double-Blind Randomized Trial. PLoS One. 2016;11(1):e0143490.

282. ZYB40005: The effect of sustained-release bupropion HCl vs. placebo as an aid to smoking reduction leading to cessation among smokers unwilling and unable to quit smoking.

283. Sonderskov J, Olsen J, Sabroe S, Meillier L, Overvad K. Nicotine patches in smoking cessation: A randomized trial among over-the-counter customers in Denmark. American Journal of Epidemiology. 1997;145(4):309-18.

284. Sorensen LT, Jorgensen T. Short-term pre-operative smoking cessation intervention does not affect postoperative complications in colorectal surgery: a randomized clinical trial. Colorectal Dis. 2003;5(4):347-52.

285. Southern Illinois University Carbondale, National Institute on Drug Abuse. Nicotine Replacement Therapy (NRT) and Bupropion Mechanisms of Effectiveness in Smokers. https://ClinicalTrials.gov/show/NCT01048944; 2005.

286. Stanford University, Pfizer. Varenicline In-Patient Study. https://ClinicalTrials.gov/show/NCT01413516; 2011.

287. Stapleton JA, Russell MA, Feyerabend C, Wiseman SM, Gustavsson G, Sawe U, et al. Dose effects and predictors of outcome in a randomized trial of transdermal nicotine patches in general practice. Addiction. 1995;90(1):31-42.

288. Stapleton JA, Sutherland G. Treating heavy smokers in primary care with the nicotine nasal spray: randomized placebo-controlled trial. Addiction. 2011;106(4):824-32.

289. Stapleton J, West R, Hajek P, Wheeler J, Vangeli E, Abdi Z, et al. Randomized trial of nicotine replacement therapy (NRT), bupropion and NRT plus bupropion for smoking cessation: effectiveness in clinical practice. Addiction. 2013;108(12):2193-201.

290. Stein MD, Caviness CM, Kurth ME, Audet D, Olson J, Anderson BJ. Varenicline for smoking cessation among methadone-maintained smokers: a randomized clinical trial. Drug Alcohol Depend. 2013;133(2):486-93.

291. Steinberg MB, Greenhaus S, Schmelzer AC, Bover MT, Foulds J, Hoover DR, et al. Triple-combination pharmacotherapy for medically ill smokers: a randomized trial. Ann Intern Med. 2009;150(7):447-54.

292. Steinberg MB, Randall J, Greenhaus S, Schmelzer AC, Richardson DL, Carson JL. Tobacco dependence treatment for hospitalized smokers: a randomized, controlled, pilot trial using varenicline. Addict Behav. 2011;36(12):1127-32.

293. Steinberg ML, Lu SE, Williams JM. Varenicline for smoking reduction in smokers not yet ready to quit: A double-blind, proof-of-concept randomized clinical trial. Addict Behav. 2018;84:20-6.

294. Sutherland G, Stapleton JA, Russell MAH, Jarvis MJ, Hajek P, Belcher M, et al. Randomized Controlled Trial of Nasal Nicotine Spray in Smoking Cessation. Lancet. 1992;340(8815):324-9.

295. Swan GE, McAfee T, Curry SJ, Jack LM, Javitz H, Dacey S, et al. Effectiveness of bupropion sustained release for smoking cessation in a health care setting: a randomized trial. Arch Intern Med. 2003;163(19):2337-44.

296. Tashkin D, Kanner R, Bailey W, Buist S, Anderson P, Nides M, et al. Smoking cessation in patients with chronic obstructive pulmonary disease: a double-blind, placebo-controlled, randomised trial. Lancet. 2001;357(9268):1571-5.

297. Tashkin DP, Rennard S, Hays JT, Ma W, Lawrence D, Lee TC. Effects of varenicline on smoking cessation in patients with mild to moderate COPD: a randomized controlled trial. Chest. 2011;139(3):591-9.

298. Thomsen T, Tonnesen H, Okholm M, Kroman N, Maibom A, Sauerberg ML, et al. Brief smoking cessation intervention in relation to breast cancer surgery: a randomized controlled trial. Nicotine Tob Res. 2010;12(11):1118-24.

299. Thorsteinsson HS, Gillin JC, Patten CA, Golshan S, Sutton LD, Drummond S, et al. The effects of transdermal nicotine therapy for smoking cessation on depressive symptoms in patients with major depression. Neuropsychopharmacology. 2001;24(4):350-8.

300. Tidey JW, Rohsenow DJ, Kaplan GB, Swift RM, Reid N. Effects of contingency management and bupropion on cigarette smoking in smokers with schizophrenia. Psychopharmacology (Berl). 2011;217(2):279-87.

301. Tonnesen P, Fryd V, Hansen M, Helsted J, Gunnersen AB, Forchammer H, et al. Effect of nicotine chewing gum in combination with group counseling on the cessation of smoking. N Engl J Med. 1988;318(1):15-8.

302. Tonnesen P, Fryd V, Hansen M, Helsted J, Gunnersen AB, Forchammer H, et al. Two and four mg nicotine chewing gum and group counselling in smoking cessation: an open, randomized, controlled trial with a 22 month follow-up. Addict Behav. 1988;13(1):17-27.

303. Tonnesen P, Norregaard J, Simonsen K, Sawe U. A double-blind trial of a 16-hour transdermal nicotine patch in smoking cessation. N Engl J Med. 1991;325(5):311-5.

304. Tonnesen P, Norregaard J, Mikkelsen K, Jorgensen S, Nilsson F. A double-blind trial of a nicotine inhaler for smoking cessation. JAMA. 1993;269(10):1268-71.

305. Tonnesen P, Paoletti P, Gustavsson G, Russell MA, Saracci R, Gulsvik A, et al. Higher dosage nicotine patches increase one-year smoking cessation rates: results from the European CEASE trial. Collaborative European Anti-Smoking Evaluation. European Respiratory Society. Eur Respir J. 1999;13(2):238-46.

306. Tonnesen P, Mikkelsen KL. Smoking cessation with four nicotine replacement regimes in a lung clinic. Eur Respir J. 2000;16(4):717-22.

307. Tonnesen P, Tonstad S, Hjalmarson A, Lebargy F, Van Spiegel PI, Hider A, et al. A multicentre, randomized, double-blind, placebo-controlled, 1-year study of bupropion SR for smoking cessation. J Intern Med. 2003;254(2):184-92.

308. Tonnesen P, Mikkelsen K, Bremann L. Nurse-conducted smoking cessation in patients with COPD using nicotine sublingual tablets and behavioral support. Chest. 2006;130(2):334-42.

309. Tonnesen P, Lauri H, Perfekt R, Mann K, Batra A. Efficacy of a nicotine mouth spray in smoking cessation: a randomised, double-blind trial. Eur Respir J. 2012;40(3):548-54.

310. Tonstad S, Farsang C, Klaene G, Lewis K, Manolis A, Perruchoud AP, et al. Bupropion SR for smoking cessation in smokers with cardiovascular disease: a multicentre, randomised study. Eur Heart J. 2003;24(10):946-55.

311. Tonstad S, Tonnesen P, Hajek P, Williams KE, Billing CB, Reeves KR, et al. Effect of maintenance therapy with varenicline on smoking cessation: a randomized controlled trial. JAMA. 2006;296(1):64-71.

312. Tsai ST, Cho HJ, Cheng HS, Kim CH, Hsueh KC, Billing CB, Jr., et al. A randomized, placebo-controlled trial of varenicline, a selective alpha4beta2 nicotinic acetylcholine receptor partial agonist, as a new therapy for smoking cessation in Asian smokers. Clin Ther. 2007;29(6):1027-39.

313. Tseng TY, Ostroff JS, Campo A, Gerard M, Kirchner T, Rotrosen J, et al. A Randomized Trial Comparing the Effect of Nicotine Versus Placebo Electronic Cigarettes on Smoking Reduction Among Young Adult Smokers. Nicotine Tob Res. 2016;18(10):1937-43.

314. Tsukahara H, Noda K, Saku K. A randomized controlled open comparative trial of varenicline vs nicotine patch in adult smokers: efficacy, safety and withdrawal symptoms (the VN-SEESAW study). Circ J. 2010;74(4):771-8.

315. Tuisku A, Salmela M, Nieminen P, Toljamo T. Varenicline and Nicotine Patch Therapies in Young Adults Motivated to Quit Smoking: A Randomized, Placebo-controlled, Prospective Study. Basic Clin Pharmacol Toxicol. 2016;119(1):78-84.

316. Tulloch HE, Pipe AL, Els C, Clyde MJ, Reid RD. Flexible, dual-form nicotine replacement therapy or varenicline in comparison with nicotine patch for smoking cessation: a randomized controlled trial. BMC Med. 2016;14(80):80.

317. Tzivoni D, Keren A, Meyler S, Khoury Z, Lerer T, Brunel P. Cardiovascular safety of transdermal nicotine patches in patients with coronary artery disease who try to quit smoking. Cardiovasc Drugs Ther. 1998;12(3):239-44.

318. University of Michigan, National Heart LaBI. A Randomized Trial of Internet Access to Nicotine Patches. https://ClinicalTrials.gov/show/NCT00534404; 2011.

319. Uyar M, Filiz A, Bayram N, Elbek O, Herken H, Topcu A, et al. A randomized trial of smoking cessation. Medication versus motivation. Saudi Med J. 2007;28(6):922-6.

320. Velicer WF, Friedman RH, Fava JL, Gulliver SB, Keller S, Sun X, et al. Evaluating nicotine replacement therapy and stage-based therapies in a population-based effectiveness trial. J Consult Clin Psychol. 2006;74(6):1162-72.

321. Verplaetse TL, Pittman BP, Shi JM, Tetrault JM, Coppola S, McKee SA. Effect of Lowering the Dose of Varenicline on Alcohol Self-administration in Drinkers With Alcohol Use Disorders. J Addict Med. 2016;10(3):166-73.

322. Wagena EJ, Knipschild PG, Huibers MJH, Wouters EFM, Schayck CPR. Efficacy of bupropion and nortriptyline for smoking cessation among people who are at risk for or have chronic obstructive pulmonary disease: Results from a randomized, placebo-controlled trial. Nicotine & Tobacco Research. 2005;7(4):683-4.

323. Wallstrom M, Nilsson F, Hirsch JM. A randomized, double-blind, placebo-controlled clinical evaluation of a nicotine sublingual tablet in smoking cessation. Addiction. 2000;95(8):1161-71.

324. Wang C, Xiao D, Chan KP, Pothirat C, Garza D, Davies S. Varenicline for smoking cessation: a placebo-controlled, randomized study. Respirology. 2009;14(3):384-92.

325. Ward KD, Asfar T, Al Ali R, Rastam S, Weg MW, Eissenberg T, et al. Randomized trial of the effectiveness of combined behavioral/pharmacological smoking cessation treatment in Syrian primary care clinics. Addiction. 2013;108(2):394-403.

326. Warner DO, Patten CA, Ames SC, Offord KP, Schroeder DR. Effect of nicotine replacement therapy on stress and smoking behavior in surgical patients. Anesthesiology. 2005;102(6):1138-46.

327. Weaver K, Kaplan S, Urbanic J, Case D, Zbikowski S, Dakhil C, et al. Incorporating evidence-based smoking cessation into community oncology practices: Feasibility and preliminary efficacy of an enhanced quitline-based smoking cessation intervention for cancer survivors2015; 24:[32 p.]. Available from: http://onlinelibrary.wiley.com/o/cochrane/clcentral/articles/997/CN-01107997/frame.html.

328. Weinberger AH, Vessicchio JC, Sacco KA, Creeden CL, Chengappa KN, George TP. A preliminary study of sustained-release bupropion for smoking cessation in bipolar disorder. J Clin Psychopharmacol. 2008;28(5):584-7.

329. Weiner E, Buchholz A, Coffay A, Liu F, McMahon RP, Buchanan RW, et al. Varenicline for smoking cessation in people with schizophrenia: a double blind randomized pilot study. Schizophr Res. 2011;129(1):94-5.

330. Weiner E, Ball MP, Buchholz AS, Gold JM, Evins AE, McMahon RP, et al. Bupropion sustained release added to group support for smoking cessation in schizophrenia: a new randomized trial and a meta-analysis. J Clin Psychiatry. 2012;73(1):95-102.

331. Wennike P, Danielsson T, Landfeldt B, Westin A, Tonnesen P. Smoking reduction promotes smoking cessation: results from a double blind, randomized, placebo-controlled trial of nicotine gum with 2-year follow-up. Addiction. 2003;98(10):1395-402.

332. Westergaard CG, Porsbjerg C, Backer V. The effect of Varenicline on smoking cessation in a group of young asthma patients. Respir Med. 2015;109(11):1416-22.

333. Westman EC, Levin ED, Rose JE. The nicotine patch in smoking cessation. A randomized trial with telephone counseling. Arch Intern Med. 1993;153(16):1917-23.

334. Whelton H, Kingston R, O'Mullane D, Nilsson F. Randomized controlled trial to evaluate tooth stain reduction with nicotine replacement gum during a smoking cessation program. BMC Oral Health. 2012;12(13):13.

335. Wiggers LC, Smets EM, Oort FJ, Peters RJ, Storm-Versloot MN, Vermeulen H, et al. The effect of a minimal intervention strategy in addition to nicotine replacement therapy to support smoking cessation in cardiovascular outpatients: a randomized clinical trial. Eur J Cardiovasc Prev Rehabil. 2006;13(6):931-7.

336. Williams KE, Reeves KR, Billing CB, Jr., Pennington AM, Gong J. A double-blind study evaluating the long-term safety of varenicline for smoking cessation. Curr Med Res Opin. 2007;23(4):793-801.

337. Williams JM, Anthenelli RM, Morris CD, Treadow J, Thompson JR, Yunis C, et al. A randomized, double-blind, placebo-controlled study evaluating the safety and efficacy of varenicline for smoking cessation in patients with schizophrenia or schizoaffective disorder. J Clin Psychiatry. 2012;73(5):654-60.

338. Wilson JS, Fitzsimons D, Bradbury I, Stuart Elborn J. Does additional support by nurses enhance the effect of a brief smoking cessation intervention in people with moderate to severe chronic obstructive pulmonary disease? A randomised controlled trial. Int J Nurs Stud. 2008;45(4):508-17.

339. Winhusen TM, Brigham GS, Kropp F, Lindblad R, Gardin JG, 2nd, Penn P, et al. A randomized trial of concurrent smoking-cessation and substance use disorder treatment in stimulant-dependent smokers. J Clin Psychiatry. 2014;75(4):336-43.

340. Wiratmoko MR, Yunus F, Susanto AD, Ginting TT, Kekalih A. Efficacy of Varenicline, an Nicotinic Acetylcholine Receptor Partial Agonist, Vs Placebo for Smoking Cessation. A Randomized Controlled Trial. Respirology. 2013;18:66-.

341. Wittchen HU, Hoch E, Klotsche J, Muehlig S. Smoking cessation in primary care - a randomized controlled trial of bupropione, nicotine replacements, CBT and a minimal intervention. Int J Methods Psychiatr Res. 2011;20(1):28-39.

342. Wolfenden L, Wiggers J, Knight J, Campbell E, Rissel C, Kerridge R, et al. A programme for reducing smoking in pre-operative surgical patients: randomised controlled trial. Anaesthesia. 2005;60(2):172-9.

343. Wong GY, Wolter TD, Croghan GA, Croghan IT, Offord KP, Hurt RD. A randomized trial of naltrexone for smoking cessation. Addiction. 1999;94(8):1227-37.

344. Wong J, Abrishami A, Yang Y, Zaki A, Friedman Z, Selby P, et al. A perioperative smoking cessation intervention with varenicline: a double-blind, randomized, placebo-controlled trial. Anesthesiology. 2012;117(4):755-64.

345. Wong J, Abrishami A, Riazi S, Siddiqui N, You-Ten E, Korman J, et al. A Perioperative Smoking Cessation Intervention With Varenicline, Counseling, and Fax Referral to a Telephone Quitline Versus a Brief Intervention: A Randomized Controlled Trial. Anesth Analg. 2017;125(2):571-9.

346. Yale University, National Institute on Alcohol Abuse and Alcoholism. The Effect of Varenicline (Chantix) and Bupropion (Zyban) on Smoking Lapse Behavior. https://ClinicalTrials.gov/show/NCT00580853; 2007.

347. Zellweger JP, Boelcskei PL, Carrozzi L, Sepper R, Sweet R, Hider AZ. Bupropion SR vs placebo for smoking cessation in health care professionals. Am J Health Behav. 2005;29(3):240-9.

348. Zernig G, Wallner R, Grohs U, Kriechbaum N, Kemmler G, Saria A. A randomized trial of short psychotherapy versus sustained-release bupropion for smoking cessation. Addiction. 2008;103(12):2024-31.

349. Zhu J. The effectiveness of bupropion SR on depressive symptoms in smokers: Self-reports, EEG, and individual differences. Dissertation Abstracts International: Section B: The Sciences and Engineering. 2016;77(3-B(E)):No Pagination Specified.

350. ZYB40030: a multi-centre, randomised, double-blind, placebo controlled study to evaluate the efficacy and tolerability of bupropion hydrochloride (SR) sustained release versus placebo as an aid to smoking cessation in a population of smokers with chronic obstructive pulmonary disease.

351. ZYB 30011:A multicentre, randomised, double- blind, placebo controlled study to evaluate the efficacy and tolerability of bupropion hydrochloride (SR) sustained release (2 x 150mg per day) versus placebo as an aid to smoking cessation in smokers with at least one cardiovascular (CV) risk factor.

352. SMK20001:A Multi-Center, Double-Blind, Double-Dummy, Placebo-Controlled, Randomized, Parallel Group, Dose Response Evaluation of a New Chemical Entity (NCE) and ZYBAN (bupropion hydrochloride) Sustained Release (300mg/day) versus Placebo As Aids to Smoking Cessation

# Figure S.4 Risk of bias summary figure

# Table S.11 Comparison of different NMA models for safety outcomes

|  | Residual deviance | DIC | SD_d_ (95% CrI) | SD_D_ (95% CrI) |
| --- | --- | --- | --- | --- |
| **Serious adverse events (219 data points)** |  |  |  |  |
| Full interaction model, consistency | 207.1 | 1021 | 0.09 (0.01,0.29) | - |
| Random Class model, consistency | 205.6 | 1014 | 0.07  (0,0.28) | 0.2 (0.01,0.68) |
| Fixed Class model, consistency | 205.8 | 1012 | 0.09  (0,0.28) | - |
| Fixed Class model, inconsistency | 211.1 | 1027 | 0.09 (0.01,0.29) | - |
| **Major adverse cardiovascular events (91 data points)** | | | | |
| Full interaction model, consistency | 82.49 | 341.1 | 0.26 (0.01,0.82) | - |
| Random Class model, consistency | 80.3 | 366.7 | 0.26 (0.02,0.79) | 0.54 (0.02,2.75) |
| Fixed Class model, consistency | 79.51 | 334 | 0.23 (0.01,0.73) | - |
| Fixed Class model, inconsistency | 80.61 | 338.3 | 0.22 (0.01,0.72) | - |
| **Major adverse neuropsychiatric events (158 data points)** | | | | |
| Full interaction model, consistency | 154.2 | 717.5 | 0.15 (0.01,0.44) | - |
| Random Class model, consistency | 153.9 | 717.1 | 0.17 (0.01,0.45) | 0.92 (0.18,2.32) |
| Fixed Class model, consistency | 154.4 | 717.5 | 0.33 (0.05,0.60) | - |
| Fixed Class model, inconsistency | 158.2 | 722.5 | 0.18 (0.02,0.47) | - |

DIC: Deviance Information Criterion; SD_d_: standard deviation across treatment effect estimates; SD_D_: standard deviation across class effect estimates.

# Table S.12 Results for serious adverse events: comparisons with placebo

|  | **Direct evidence, OR (95% CrI)** | **Indirect evidence, OR (95% CrI)** | **NMA, OR (95% CrI)** |
| --- | --- | --- | --- |
| No Drug Treatment | - | 1.11 (0.56 to 2.29) | 1.11 (0.56 to 2.29) |
| Usual Care | - | 0.28 (0.02 to 2.20) | 0.28 (0.02 to 2.20) |
| NRT ns | 1.19 (0.86 to 1.62) | 0.72 (0.31 to 1.68) | 1.12 (0.79 to 1.49) |
| NRT Std | 1.11 (0.74 to 1.68) | 1.37 (0.38 to 4.96) | 1.13 (0.76 to 1.67) |
| NRT High | 1.11 (0.77 to 1.58) | 1.40 (0.87 to 2.27) | 1.21 (0.89 to 1.63) |
| Bupropion Low | 0.26 (0.03 to 1.52) | 0.37 (0.01 to 9.36) | 0.21 (0.01 to 1.57) |
| Bupropion Std | 1.27 (1.04 to 1.58) | - | 1.27 (1.04 to 1.58) |
| Varenicline Low | 1.12 (0.54 to 2.29) | 1.62 (0.15 to 17.6) | 1.07 (0.40 to 2.29) |
| Varenicline Std | 1.09 (0.91 to 1.34) | - | 1.09 (0.91 to 1.34) |
| E-Cigarette Low | - | 7.24 (0.46 to 3262) | 7.24 (0.46 to 3262) |
| E-Cigarette High | 1.99 (0.90 to 4.44) | 1.44 (0.61 to 3.42) | 1.72 (0.77 to 2.89) |
| Bupropion Low + NRT High | - | 1.19 (0.24 to 7.46) | 1.19 (0.24 to 7.46) |
| Bupropion Std + NRT ns | - | 0.49 (0.03 to 3.35) | 0.49 (0.03 to 3.35) |
| Bupropion Std + NRT High | 0.73 (0.05 to 4.18) | 1.57 (0.47 to 5.28) | 1.31 (0.49 to 3.67) |
| Varenicline Low + NRT Std | - | 6.62 (0.09 to 4964) | 6.62 (0.09 to 4964) |
| Varenicline Std + NRT Std | - | 1.38 (0.33 to 9.78) | 1.38 (0.33 to 9.78) |
| Varenicline Std + NRT High | - | 1.11 (0.29 to 3.49) | 1.11 (0.29 to 3.49) |
| Varenicline Std + Bupropion Std | 2.51 (0.73 to 9.49) | 1.48 (0.57 to 3.84) | 1.79 (0.86 to 4.06) |

# Table S.13 Results for serious adverse events: pair-wise comparisons of interventions

|  | **Direct evidence, OR (95% CrI)** | **Indirect evidence, OR (95% CrI)** | **NMA, OR (95% CrI)** |
| --- | --- | --- | --- |
| Bupropion Std vs. NRT Std |  | 1.12 (0.72 to 1.80) | 1.12 (0.72 to 1.80) |
| Varenicline Std vs. NRT Std |  | 0.98 (0.62 to 1.50) | 0.98 (0.62 to 1.50) |
| E-Cigarette Low vs. NRT Std |  | 6.48 (0.39 to 3087) | 6.48 (0.39 to 3087) |
| E-Cigarette High vs. NRT Std |  | 1.54 (0.58 to 2.90) | 1.54 (0.58 to 2.90) |
| Varenicline Std + NRT Std vs. NRT Std |  | 1.23 (0.28 to 8.39) | 1.23 (0.28 to 8.39) |
| Varenicline Std + Bupropion Std vs. NRT Std |  | 1.60 (0.69 to 3.82) | 1.60 (0.69 to 3.82) |
| Varenicline Std vs. Bupropion Std |  | 0.86 (0.67 to 1.11) | 0.86 (0.67 to 1.11) |
| E-Cigarette Low vs. Bupropion Std |  | 5.75 (0.37 to 2419) | 5.75 (0.37 to 2419) |
| E-Cigarette High vs. Bupropion Std |  | 1.34 (0.69 to 2.28) | 1.34 (0.69 to 2.28) |
| Varenicline Std + NRT Std vs. Bupropion Std |  | 1.10 (0.25 to 8.09) | 1.10 (0.25 to 8.09) |
| Varenicline Std + Bupropion Std vs. Bupropion Std |  | 1.39 (0.67 to 3.20) | 1.39 (0.67 to 3.20) |
| E-Cigarette Low vs. Varenicline Std |  | 6.36 (0.42 to 2879) | 6.36 (0.42 to 2879) |
| E-Cigarette High vs. Varenicline Std |  | 1.56 (0.74 to 2.67) | 1.56 (0.74 to 2.67) |
| Varenicline Std + NRT Std vs. Varenicline Std | 1.28 (0.30 to 7.77) | - | 1.28 (0.30 to 7.77) |
| Varenicline Std + Bupropion Std vs. Varenicline Std | 1.24 (0.49 to 3.34) | 2.55 (0.78 to 8.31) | 1.64 (0.80 to 3.59) |
| E-Cigarette High vs. E-Cigarette Low |  | 0.23 (0.00 to 3.47) | 0.23 (0.00 to 3.47) |
| Varenicline Std + NRT Std vs. E-Cigarette Low |  | 0.21 (0.00 to 5.40) | 0.21 (0.00 to 5.40) |
| Varenicline Std + Bupropion Std vs. E-Cigarette Low |  | 0.27 (0.00 to 4.28) | 0.27 (0.00 to 4.28) |
| Varenicline Std + NRT Std vs. E-Cigarette High |  | 0.83 (0.17 to 5.52) | 0.83 (0.17 to 5.52) |
| Varenicline Std + Bupropion Std vs. E-Cigarette High |  | 1.07 (0.42 to 3.31) | 1.07 (0.42 to 3.31) |
| Varenicline Std + Bupropion Std vs. Varenicline Std + NRT Std |  | 1.33 (0.19 to 6.31) | 1.33 (0.19 to 6.31) |

# Results of threshold analysis for serious adverse events

Threshold analysis, a form of sensitivity analysis, was performed to determine the robustness of our treatment recommendations for the primary safety outcome to changes in the evidence provided by the individual studies.^6,7^ Threshold analysis determines how much the evidence could change - for any reason, such a bias or random error - before the treatment ranking changes and describes this using a set of thresholds. These thresholds can be compared with judgements of the plausible magnitude of potential biases and with estimates of uncertainty (e.g. confidence intervals). In this manner, we may have more confidence in conclusions that are shown to be robust, and can appropriately acknowledge where conclusions are shown to be sensitive to plausible biases or uncertainty in the evidence.

Results of threshold analyses for the first- and last-ranked treatments for serious adverse events are shown in *the first and second Figure*, respectively. The first-ranked treatment is Placebo, and the last-ranked is E-Cigarette Low. Both figures also include the risk of bias judgements from *the appendix pp 74–84*.

The first-place ranking (of Placebo) is very sensitive to the level of uncertainty in the evidence, as shown by the number of study estimates with 95% Confidence Intervals that cross the corresponding threshold. At each of these thresholds, the new first-ranked treatment is either NRT Std, Varenicline Std + NRT Std, E-Cigarette High, E-Cigarette Low, or Varenicline + Bupropion Std. Five studies (Nides et al. 2018,^386^ Koegelenberg et al. 2014,^351^ Shiffman et al. 2002,^444^ Tonnesen et al. 2012,^468^ and Rennard et al. 2006)^415^ have thresholds smaller than 0.7 log OR (equivalent to a factor of 2 on the odds ratio scale), and of these only Tonnesen et al. 2012 is rated at low risk of bias.

The last-place ranking (of E-Cigarette Low) is only sensitive to the level of uncertainty from one study, Cravo et al. 2016 (E-Cigarette Low vs. No Drug Treatment).^526^ The first-place ranking is also sensitive to the level of uncertainty in this study estimate. If the estimate were to change by -0.80 log OR from 1.32 to 0.52 in favour of E-Cigarette Low then E-Cigarette Low would no longer be ranked last (replaced by Varenicline + Bupropion Std), and if the estimate were to change only a little more to 0.24 then E-Cigarette Low would be ranked first for serious adverse events. Due to the high level of uncertainty in this study estimate and the resulting wide confidence interval, both of these are plausible due to sampling error. Cravo et al. 2016 has the smallest threshold for the last-place ranking (-0.80 log OR, equivalent to a factor of 2.2 on the OR scale) and was judged at high risk of bias due to inadequate blinding and other bias. The remaining thresholds for the other 122 study estimates are equivalent to a factor of 8 or greater on the OR scale, which may be larger than any plausible biases.

Overall, the first- and last-place rankings for serious adverse events are not very robust: both are sensitive to the level of uncertainty in the data, and there may be plausible biases in studies at high or unclear risk of bias that could result in a change of first- or last-place ranking. The Cravo et al. 2016^54^ study is notably influential for both first- and last-place rankings, displays high levels of uncertainty, and is at high risk of bias. The last-place ranking is likely robust to plausible biases in all other studies.

6. Phillippo DM, Dias S, Ades AE, Didelez V, Welton NJ. Sensitivity of treatment recommendations to bias in network meta-analysis. J R Stat Soc Ser A Stat Soc. 2018;181(3):843-67.

7. Phillippo DM, Dias S, Welton NJ, Caldwell DM, Taske N, Ades AE. Threshold Analysis as an Alternative to GRADE for Assessing Confidence in Guideline Recommendations Based on Network Meta-analysesThreshold Analysis in Guideline Development. Annals of Internal Medicine. 2019;170(8):538-46.


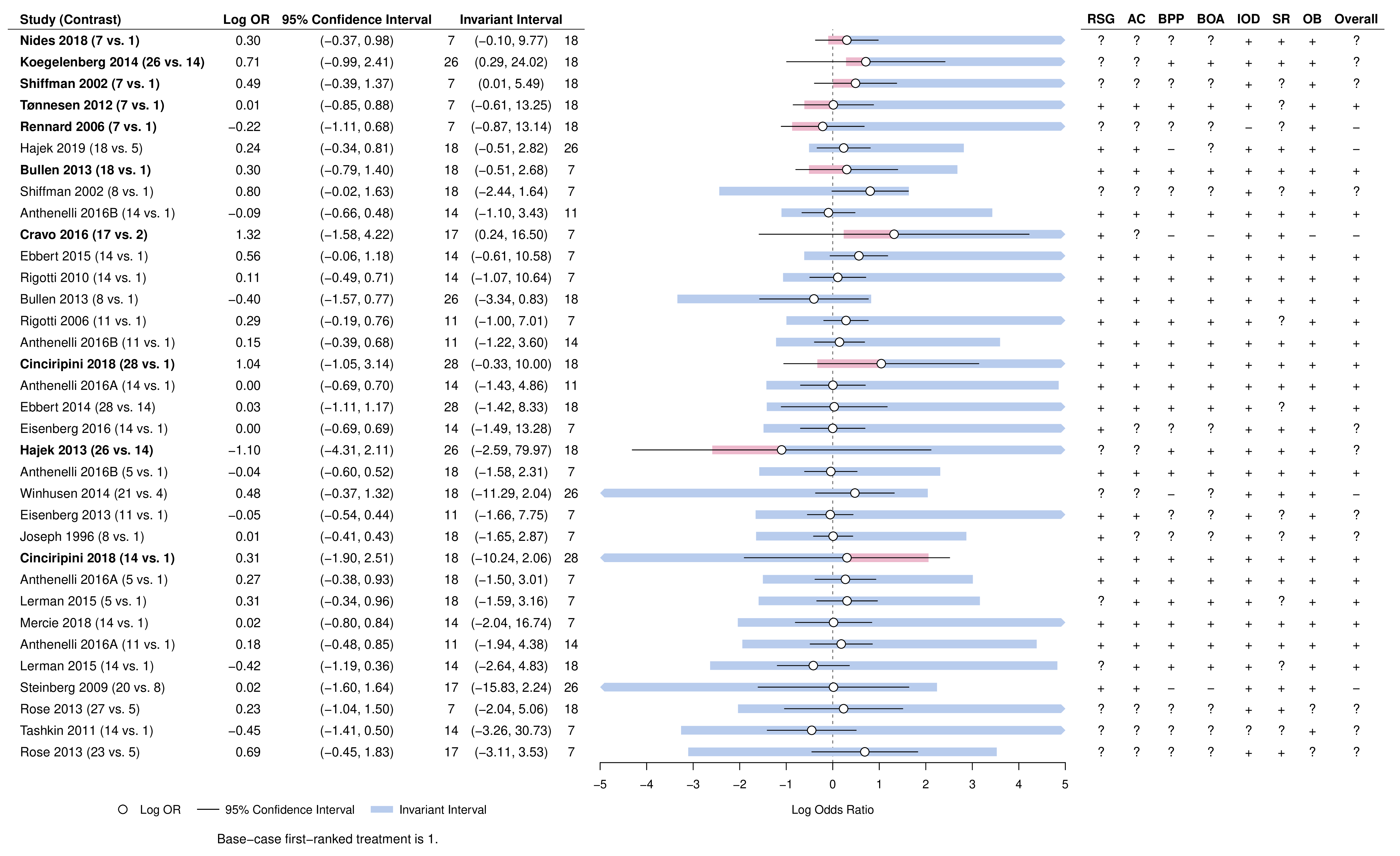


Figure S.5 Threshold analysis results for serious adverse events (first-ranked treatment), sorted by size of threshold (smallest to largest)

Only studies with thresholds less than 3 log OR are shown, for brevity. Treatment codes are: (1) Placebo (2) No Drug Treatment (4) Usual Care (5) NRT ns (7) NRT Std (8) NRT High (11) Bupropion Std (14) Varenicline Std (17) E-Cigarette Low (18) E-Cigarette High (20) Bupropion Low + NRT High (21) Bupropion Std + NRT ns (23) Bupropion Std + NRT High (26) Varenicline Std + NRT Std (27) Varenicline Std + NRT High (28) Varenicline + Bupropion Std. Bold study labels and red shaded invariant intervals show where a 95% confidence interval crosses the corresponding threshold, indicating sensitivity to the level of uncertainty in this estimate. NT = no threshold. RSG = random sequence generation; AC = allocation concealment; BPP = blinding of participants and personnel; BOA = blinding of outcome assessment; IOD = incomplete outcome data; SR = selective reporting; OB = other bias. “+” = low risk of bias; “-“ = high risk of bias; “?” = unclear risk of bias.

^19, 33, 192, 207, 238, 239, 243, 244, 340, 362, 373, 386, 415, 418, 419, 422, 444, 457, 465, 468, 501, 512, 526, 539^


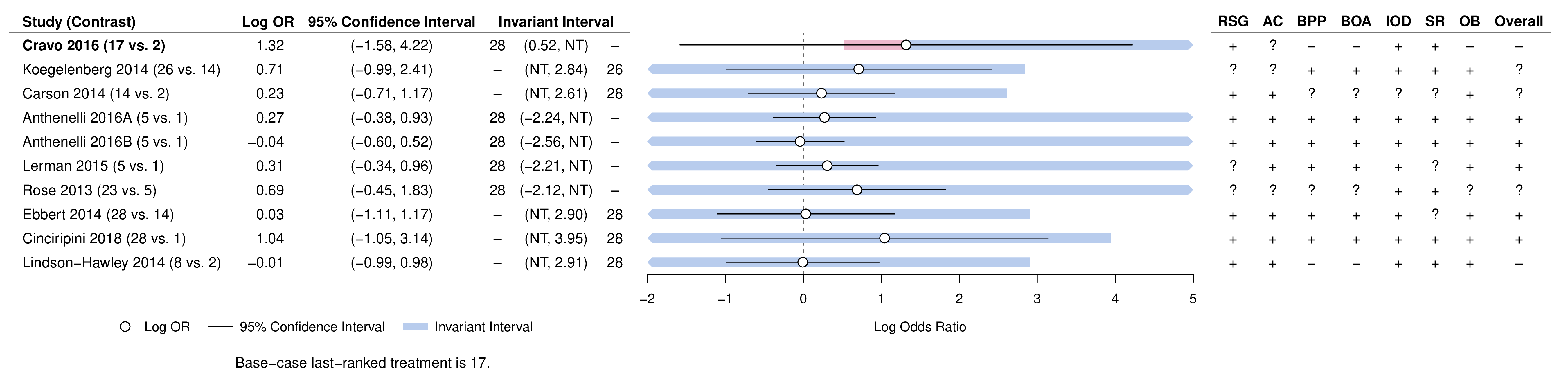


Figure S.6 Threshold analysis results for serious adverse events (last-ranked treatment), sorted by size of threshold (smallest to largest)

Only studies with thresholds less than 3 log OR are shown, for brevity. Treatment codes are: (1) Placebo (2) No Drug Treatment (5) Usual Care (7) NRT Std (8) NRT High (11) Bupropion Std (14) Varenicline Std (17) E-Cigarette Low (18) E-Cigarette High (23) Bupropion Std + NRT High (26) Varenicline Std + NRT Std (28) Varenicline + Bupropion Std. Bold study labels and red shaded invariant intervals show where a 95% confidence interval crosses the corresponding threshold, indicating sensitivity to the level of uncertainty in this estimate. NT = no threshold. RSG = random sequence generation; AC = allocation concealment; BPP = blinding of participants and personnel; BOA = blinding of outcome assessment; IOD = incomplete outcome data; SR = selective reporting; OB = other bias. “+” = low risk of bias; “-“ = high risk of bias; “?” = unclear risk of bias.

^33, 203, 207, 238, 362, 422, 512, 526, 550^

# Table S.14 Results for major adverse cardiovascular events: comparisons with placebo

|  | **Direct evidence, OR (95% CrI)** | **Indirect evidence, OR (95% CrI)** | **NMA, OR (95% CrI)** |
| --- | --- | --- | --- |
| No Drug Treatment | - | 1.08 (0.24 to 5.26) | 1.08 (0.24 to 5.26) |
| NRT ns | 0.82 (0.30 to 2.03) | 0.61 (0.15 to 2.52) | 0.75 (0.31 to 1.57) |
| NRT Std | 1.28 (0.21 to 8.67) | 0.57 (0.03 to 9.47) | 1.01 (0.22 to 4.66) |
| NRT High | 0.44 (0.11 to 1.75) | 2.98 (0.71 to 12.5) | 1.12 (0.42 to 3.03) |
| Bupropion Low | 0.15 (0.00 to 3.25) | - | 0.15 (0.00 to 3.25) |
| Bupropion Std | 1.31 (0.68 to 2.48) | 0.95 (0.08 to 11.0) | 1.28 (0.71 to 2.36) |
| Varenicline Std | 0.76 (0.41 to 1.25) | - | 0.76 (0.41 to 1.25) |
| E-Cigarette High | 2.61 (0.44 to 28.22) | 26.8 (0.39 to 1860) | 4.01 (0.79 to 29.7) |
| Bupropion Std + NRT Std | 0.33 (0.00 to 7.24) | - | 0.33 (0.00 to 7.24) |
| Bupropion Std + NRT High | - | 0.08 (0.00 to 1.73) | 0.08 (0.00 to 1.73) |
| Varenicline Std + NRT High | - | 0.47 (0.04 to 3.82) | 0.47 (0.04 to 3.82) |
| Varenicline Std + Bupropion Std | 0.08 (0.00 to 1.34) | - | 0.08 (0.00 to 1.34) |

# Table S.15 Results for major adverse cardiovascular events: pair-wise comparisons of interventions

|  | **Direct evidence, OR (95% CrI)** | **Indirect evidence, OR (95% CrI)** | **NMA, OR (95% CrI)** |
| --- | --- | --- | --- |
| Bupropion Std vs. NRT Std | - | 1.26 (0.26 to 6.15) | 1.26 (0.26 to 6.15) |
| Varenicline Std vs. NRT Std | 0.99 (0.03 to 49.3) | 0.68 (0.12 to 3.84) | 0.73 (0.15 to 3.39) |
| E-Cigarette High vs. NRT Std | - | 4.35 (0.42 to 43.1) | 4.35 (0.42 to 43.1) |
| Bupropion Std + NRT Std vs. NRT Std | - | 0.33 (0.00 to 9.83) | 0.33 (0.00 to 9.83) |
| Varenicline Std + Bupropion Std vs. NRT Std | - | 0.07 (0.00 to 1.96) | 0.07 (0.00 to 1.96) |
| Varenicline Std vs. Bupropion Std | - | 0.58 (0.27 to 1.19) | 0.58 (0.27 to 1.19) |
| E-Cigarette High vs. Bupropion Std | - | 3.17 (0.57 to 25.3) | 3.17 (0.57 to 25.3) |
| Bupropion Std + NRT Std vs. Bupropion Std | - | 0.26 (0.00 to 5.50) | 0.26 (0.00 to 5.50) |
| Varenicline Std + Bupropion Std vs. Bupropion Std | - | 0.06 (0.00 to 1.14) | 0.06 (0.00 to 1.14) |
| E-Cigarette High vs. Varenicline Std | - | 5.51 (1.05 to 40.1) | 5.51 (1.05 to 40.1) |
| Bupropion Std + NRT Std vs. Varenicline Std | - | 0.44 (0.00 to 10.4) | 0.44 (0.00 to 10.4) |
| Varenicline Std + Bupropion Std vs. Varenicline Std | - | 0.11 (0.00 to 1.88) | 0.11 (0.00 to 1.88) |
| Bupropion Std + NRT Std vs. E-Cigarette High | - | 0.07 (0.00 to 2.97) | 0.07 (0.00 to 2.97) |
| Varenicline Std + Bupropion Std vs. E-Cigarette High | - | 0.02 (0.00 to 0.55) | 0.02 (0.00 to 0.55) |
| Varenicline Std + Bupropion Std vs. Bupropion Std + NRT Std | - | 0.20 (0.00 to 567) | 0.20 (0.00 to 567) |

# Table S.16 Results for major adverse neuropsychiatric events: comparisons with placebo

|  | **Direct evidence, OR (95% CrI)** | **Indirect evidence, OR (95% CrI)** | **NMA, OR (95% CrI)** |
| --- | --- | --- | --- |
| No Drug Treatment | - | 0.27 (0.05 to 1.05) | 0.27 (0.05 to 1.05) |
| Wait List | 0.03 (0.00 to 0.42) | - | 0.03 (0.00 to 0.42) |
| Usual Care | - | 0.48 (0.04 to 4.18) | 0.48 (0.04 to 4.18) |
| NRT ns | 0.73 (0.49 to 1.09) | 5.16 (1.43 to 18.7) | 0.60 (0.38 to 0.89) |
| NRT Std | 0.12 (0.00 to 1.93) | 0.04 (0.00 to 5506) | 0.10 (0.00 to 2.20) |
| NRT High | 0.73 (0.38 to 1.38) | 0.80 (0.14 to 4.70) | 0.74 (0.40 to 1.35) |
| Bupropion Std | 0.62 (0.44 to 0.82) | 0.47 (0.25 to 0.87) | 0.67 (0.47 to 0.91) |
| Varenicline ns | 1.60 (0.21 to 15.2) | - | 1.60 (0.21 to 15.2) |
| Varenicline Low | 0.99 (0.06 to 11.1) | - | 0.99 (0.06 to 11.1) |
| Varenicline Std | 0.96 (0.76 to 1.21) | - | 0.96 (0.76 to 1.21) |
| Bupropion Std + NRT ns | 2.39 (0.17 to 22.7) | 0.94 (0.31 to 2.83) | 1.11 (0.39 to 2.97) |
| Bupropion Std + NRT High | - | 0.21 (0.03 to 0.92) | 0.21 (0.03 to 0.92) |
| Varenicline Std + NRT Std | - | 1.72 (0.34 to 10.9) | 1.72 (0.34 to 10.9) |
| Varenicline Std + NRT High | - | 0.64 (0.17 to 2.10) | 0.64 (0.17 to 2.10) |
| Varenicline Std + Bupropion Std | 0.06 (0.00 to 1.05) | 0.23 (0.01 to 5.97) | 0.15 (0.01 to 1.12) |

# Table S.17 Results for major adverse neuropsychiatric events: pair-wise comparisons of interventions

|  | **Direct evidence, OR (95% CrI)** | **Indirect evidence, OR (95% CrI)** | **NMA, OR (95% CrI)** |
| --- | --- | --- | --- |
| Bupropion Std vs. NRT Std | - | 6.55 (0.29 to 3523) | 6.55 (0.29 to 3523) |
| Varenicline Std vs. NRT Std | - | 9.43 (0.43 to 4989) | 9.43 (0.43 to 4989) |
| Varenicline Std + NRT Std vs. NRT Std | - | 18.52 (0.49 to 10312) | 18.52 (0.49 to 10312) |
| Varenicline Std + Bupropion Std vs. NRT Std | - | 1.45 (0.02 to 950) | 1.45 (0.02 to 950) |
| Varenicline Std vs. Bupropion Std | - | 1.43 (1.02 to 2.09) | 1.43 (1.02 to 2.09) |
| Varenicline Std + NRT Std vs. Bupropion Std | - | 2.58 (0.49 to 16.6) | 2.58 (0.49 to 16.6) |
| Varenicline Std + Bupropion Std vs. Bupropion Std | - | 0.22 (0.01 to 1.73) | 0.22 (0.01 to 1.73) |
| Varenicline Std + NRT Std vs. Varenicline Std | 1.80 (0.35 to 11.2) | - | 1.80 (0.35 to 11.2) |
| Varenicline Std + Bupropion Std vs. Varenicline Std | 0.22 (0.00 to 5.43) | 0.12 (0.00 to 3.20) | 0.15 (0.01 to 1.16) |
| Varenicline Std + Bupropion Std vs. Varenicline Std + NRT Std | - | 0.08 (0.00 to 1.19) | 0.08 (0.00 to 1.19) |

# Table S.18 Average proportion of patients with an event in the placebo arm across trials for each safety outcome

| **Outcome** | **Average proportion of patients with event** |
| --- | --- |
| SAEs | 0.026 |
| MACEs | 0.005 |
| MANEs | 0.044 |

# 
